# Identification of a Series Containing a Pentafluorophenyl Moiety That Targets Pks13 to Inhibit Growth of *Mycobacterium tuberculosis*

**DOI:** 10.1021/acsinfecdis.4c00808

**Published:** 2025-02-27

**Authors:** Simon R. Green, Justin R. Harrison, Stephen Thompson, Dinakaran Murugesan, M. Daben J. Libardo, Curtis A. Engelhart, Jaclynn Meshanni, Daniel Fletcher, Paul Scullion, Darren Edwards, Ola Epemolu, Nicole Mutter, Yoko Shishikura, Jennifer Riley, Thomas R. Ioerger, Jose Juan Roca Guillén, Laura Guijarro López, Kevin D. Read, Clifton E. Barry, Dirk Schnappinger, Paul G. Wyatt, Helena I. M. Boshoff, Laura A. T. Cleghorn

**Affiliations:** † Drug Discovery Unit, Division of Biological Chemistry and Drug Discovery, School of Life Sciences, University of Dundee, Dundee DD1 5EH, U.K.; ‡ Tuberculosis Research Section, Laboratory of Clinical Immunology and Microbiology, NIAID, NIH, Rockville Pike, Bethesda, Maryland 9000, United States; § Department of Microbiology and Immunology, Weill Cornell Medical College, New York, New York 10065, United States; ∥ Department of Computer Science and Engineering, Texas A&M University, College Station, Texas 77843, United States; ⊥ Global Health Medicines R&D, GlaxoSmithKline, Severo Ochoa 2, Tres Cantos, 28760 Madrid, Spain

**Keywords:** tuberculosis, Pks13, pentafluorophenyl, metabolic instability

## Abstract

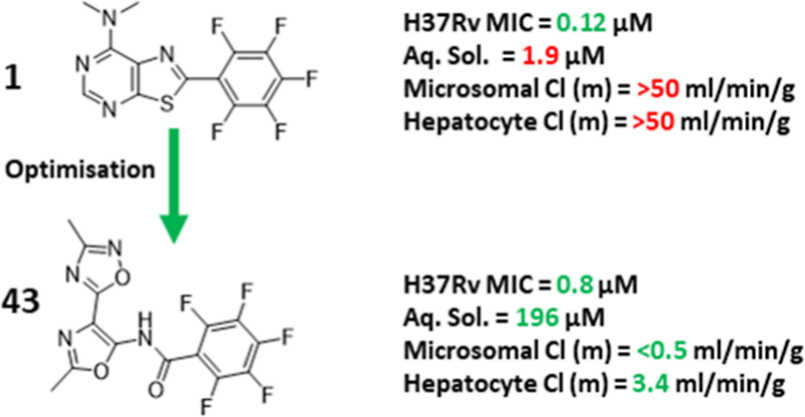

Although not currently in the infectious disease spotlight, there is still a pressing need for new agents to treat tuberculosis caused by *Mycobacterium tuberculosis*. As there is an ever-increasing amount of clinical resistance to the current drugs, ideally new drugs would be found against novel targets to circumvent pre-existing resistance. A phenotypic growth screen identified a novel singleton, **1**, as an inhibitor of *M. tuberculosis* growth. Mechanism-of-action studies determined that **1** targeted Pks13, an essential enzyme in cell wall biosynthesis that, as of yet, has not been targeted by agents in the clinic. The reactive nature of the pentafluorophenyl warhead meant that the molecule was inherently metabolically unstable. A medicinal chemistry optimization program is described that resulted in the identification of a compound that was reactive enough to still inhibit Pks13 and *M. tuberculosis* growth while being metabolically stable enough to explore in vivo.

It is estimated that 25% of the world’s population is infected with *Mycobacterium tuberculosis*, of which between 5 and 10% will go on to develop tuberculosis (TB) during their lifetime. In 2022, there were approximately 11 million new cases of TB worldwide, with Southeast Asia (46%), Africa (23%), and the Western Pacific (18%) particularly affected.^1^ It is one of the leading causes of death worldwide and prior to the COVID-19 pandemic was the main cause of death from an infectious agent, with 1.5 million deaths in 2020.^2^ The COVID-19 pandemic has reversed years of progress in providing essential TB services and controlling TB disease burden primarily due to reduced access to diagnosis and treatment.^2^ COVID-19 is estimated to have resulted in almost half a million excess deaths from TB between 2020 and 2022, compared to prepandemic trends.^1^ As such, progress toward reducing the TB disease burden is far from achieving the original WHO End TB Strategy goals (to reduce TB deaths by 95% and to cut new cases by 90% between 2015 and 2035).^1,3^

The standard frontline treatment for drug-sensitive TB lasts for 6 months and uses 4 drugs, all identified over 50 years ago; the unusually long course of treatment gives rise to noncompliance which in turn leads to resistance and increased transmission.^4−7^ Multi-, extensively-, and totally drug-resistant TB (MDR-TB, XDR-TB, TDR-TB) all require the use of a more complicated cocktail of drugs that requires a treatment period of up to 20 months.^8^ Increased investment in TB drug discovery has led to the identification of several new compounds entering clinical development (https://www.newtbdrugs.org/), although none of them have entered frontline treatment regimens to date. Given the complex pathogenicity of the disease and the requirement for treatment with multidrug cocktails, there is still an urgent need to identify drugs with novel mechanisms of action.

From a phenotypic growth screen of a large structurally diverse compound library, **1** was identified as a singleton which showed potent *M. tuberculosis* growth inhibition. Herein we describe the identification of the mechanism of action (MoA) of the initial hit and the subsequent optimization program to evaluate whether a suitable lead molecule could be obtained.

## Results and Discussion

**Figure 1:**
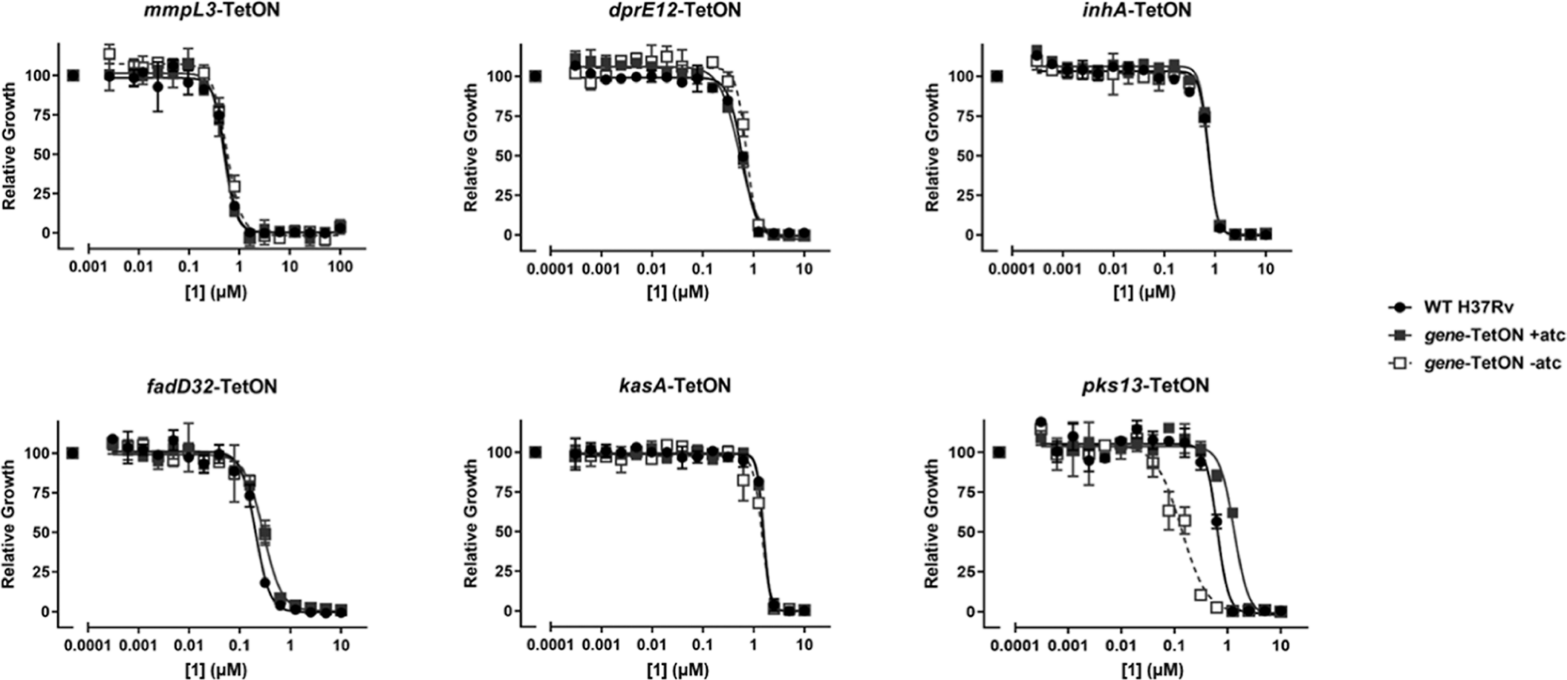
Decreased Pks13 expression results in hypersensitivity to **1**. Removal of anhydrotetracycline (atc) results in transcriptional repression of the indicated cell wall pathway gene (*mmpL3, dprE1, inhA, kasA, kasB,* and *pks13*) Growth in the presence of **1** relative to a DMSO control is shown for H37Rv and each hypomorph strain ±atc. Data are representative of two independent experiments. Relative growth indicates normalization to the growth of each strain in the absence of inhibitors.

Through a collaboration with the NIAID, several multithousand compound libraries have been screened phenotypically against *M. tuberculosis*. One such screening campaign, involving a library of ∼225,000 compounds, yielded a very potent hit **1** that also had excellent selectivity toward human HepG2 cells (Table 1). To assess the activity of this series against *M. tuberculosis*, the minimal inhibitory concentration (MIC) was determined across four media: two based on dipalmitoylphosphatidylcholine (DPPC) and two based on glucose, each pair being ±BSA. The two carbon sources were used to evaluate the activity of the compounds under different growth conditions. The MIC for the DPPC media is shown in the following tables, while the glucose medium data are included in the Data and PAINS summary in the Supporting Information. The growth inhibitory effects were similar in both carbon sources, the most striking potency differences being seen when BSA was present (Tables 1–5). This is a common difference seen across multiple series that we have evaluated, likely driven by differences in protein binding impacting the concentration of unbound compounds in the BSA-containing media. In order to better understand the MoA, **1** was assessed in several early biological profiling assays. One of these profiled the compound against a P*iniB*-LUX strain, in which the bacterial luciferase *luxCDABE* cassette is present downstream of the *iniBAC* promoter, which gives a bioluminescent readout for compounds that disrupt cell wall biosynthesis.^9,10^ A strong luminescent signal was seen following 24 h of exposure to **1**, suggesting that it was active against *M. tuberculosis* by targeting a cell wall biosynthetic process (Figure S1). Many potent hits that are identified by phenotypic screening against *M. tuberculosis* target promiscuous membrane-bound proteins that are involved in cell wall biosynthesis such as MmpL3 and DprE1.^11^ As there are already numerous molecules against both of these targets, at various stages of drug development, including clinical trials, additional early hits against these two targets are not considered desirable. Consequently, to identify which protein **1** was targeting within the cell wall biosynthesis pathway, it was profiled against a panel of cell wall hypomorph strains (Figure 1). In these strains, the removal of anhydrotetracycline (atc) results in silencing of the gene of interest. In many cases, chemical inhibition of the gene product during downregulation of its expression results in increased growth inhibition compared to control strains.^12^ Experiments with strains that allow reducing expression of either MmpL3 or DprE1 suggested that **1** was not inhibiting MmpL3 or DprE1, the two most promiscuous cell wall targets. In addition, **1** did not increase in potency when either InhA, FadD32, or Kas A was partially depleted. However, a very clear response was seen in a Pks13 hypomorph strain (Figure 1) with an ∼10-fold shift in IC_50_ between the ± atc conditions. The Pks13 hypomorph was previously demonstrated to be hypersusceptible to inhibitors targeting Pks13 but not more susceptible to inhibitors of other targets such as RNA polymerase.^13−15^ Thus, it appeared that **1** was blocking the growth of *M. tuberculosis* by inhibition of Pks13, the essential polyketide synthase that produces alpha-alkyl beta-ketoacid precursors of cell wall mycolic acids.^16−18^

**Table 1:**
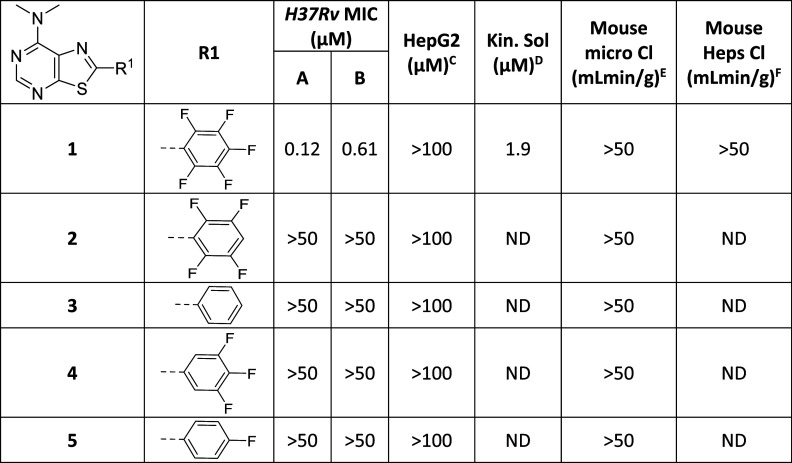
In Vitro Profile of the Initial Hit and Evaluation of Warhead SAR

MIC required to inhibit the growth of *M. tuberculosis* (H37Rv) in liquid culture (^A^7H9/DPPC/CAS/Tx; ^B^7H9/DPPC/CHOL/BSA). ^C^HepG2 inhibitory concentration (IC_50_) is the concentration required to inhibit the growth of HepG2 cells by 50%. ^D^Kinetic solubility in water; ^E^intrinsic clearance (Cli) using CD1 mouse liver microsomes; ^F^intrinsic clearance (Cli) using mouse hepatocytes. ND is not done.

**Figure 2:**
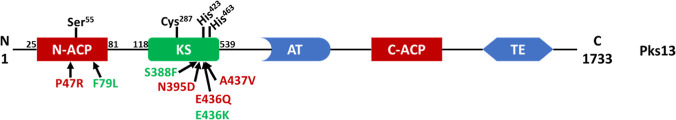
Representation of Pks13 binding domains highlighting the location of mutations in resistant strains to 1 obtained at either 5 times (red) or 10 times (green) MIC.

**Table 2:**
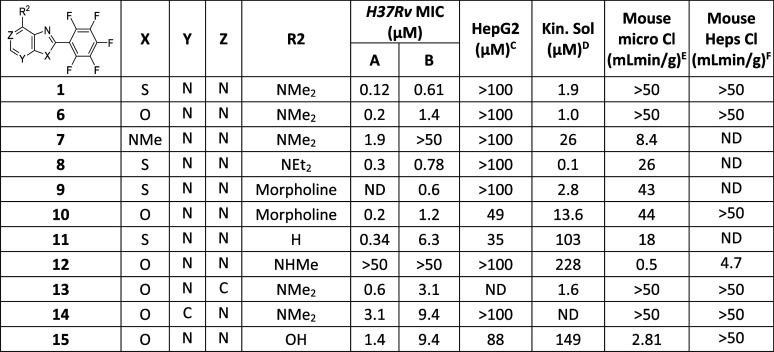
Core Modifications

MIC required to inhibit the growth of *M. tuberculosis* (H37Rv) in liquid culture (^A^7H9/DPPC/CAS/Tx; ^B^7H9/DPPC/CHOL/BSA). ^C^HepG2 inhibitory concentration (IC_50_) is the concentration required to inhibit the growth of HepG2 cells by 50%. ^D^Kinetic solubility in water; ^E^intrinsic clearance (Cli) using CD1 mouse liver microsomes; ^F^intrinsic clearance (Cli) using mouse hepatocytes. ND is not done.

**Table 3:**
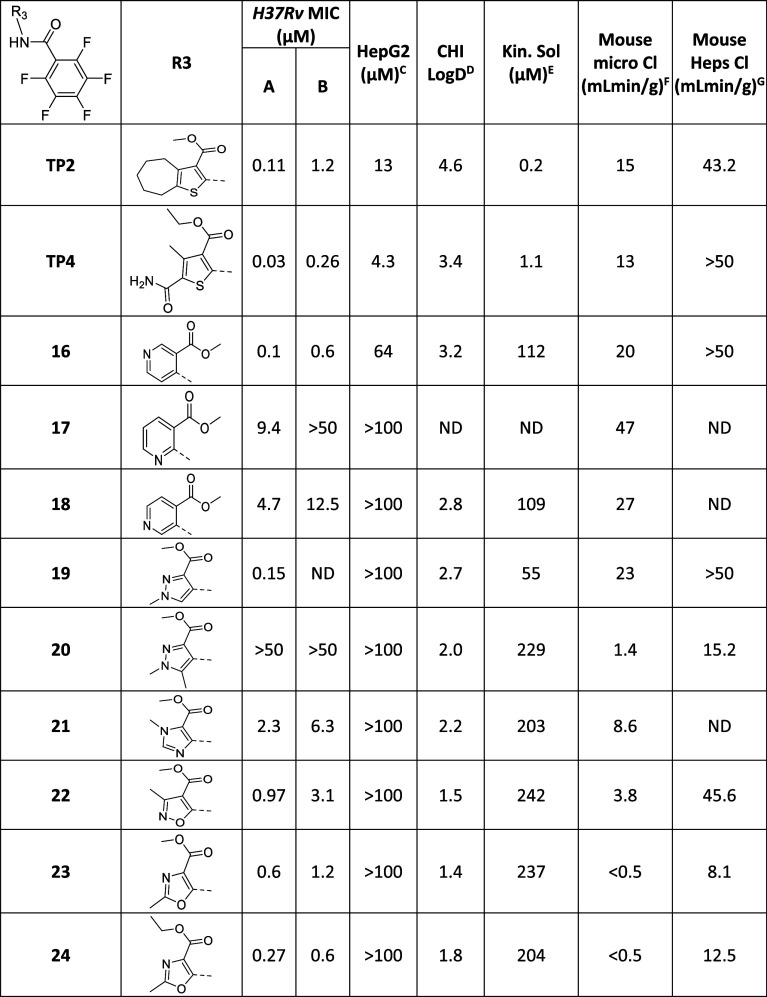
Thiophene Core Modifications

MIC required to inhibit the growth of *M. tuberculosis* (H37Rv) in liquid culture (^A^7H9/DPPC/CAS/Tx; ^B^7H9/DPPC/CHOL/BSA). ^C^HepG2 inhibitory concentration (IC_50_) is the concentration required to inhibit the growth of HepG2 cells by 50%, ^D^CHI-LogD_pH7.4_ is a measure of lipophilicity at pH7.4; ^E^Kinetic solubility in water; ^F^intrinsic clearance (Cli) using CD1 mouse liver microsomes; ^G^intrinsic clearance (Cli) using mouse hepatocytes. ND is not done.

**Table 4:**
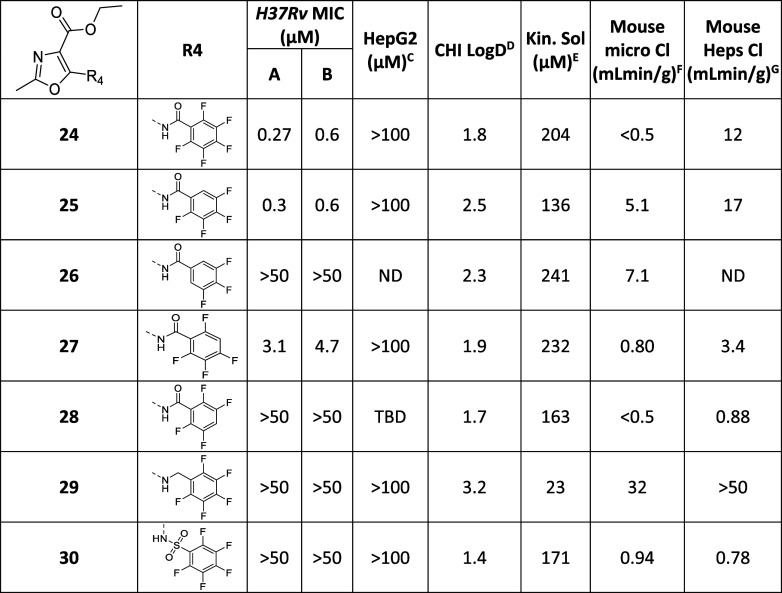
Expansion of Warhead SAR

MIC required to inhibit the growth of *M. tuberculosis* (H37Rv) in liquid culture (^A^7H9/DPPC/CAS/Tx; ^B^7H9/DPPC/CHOL/BSA). ^C^HepG2 inhibitory concentration (IC_50_) is the concentration required to inhibit growth of HepG2 cells by 50%, ^D^CHI-LogD_pH7.4_ is a measure of lipophilicity at pH 7.4; ^E^Kinetic solubility in water; ^F^intrinsic clearance (Cli) using CD1 mouse liver microsomes; ^G^intrinsic clearance (Cli) using mouse hepatocytes. ND is not done.

**Table 5:**
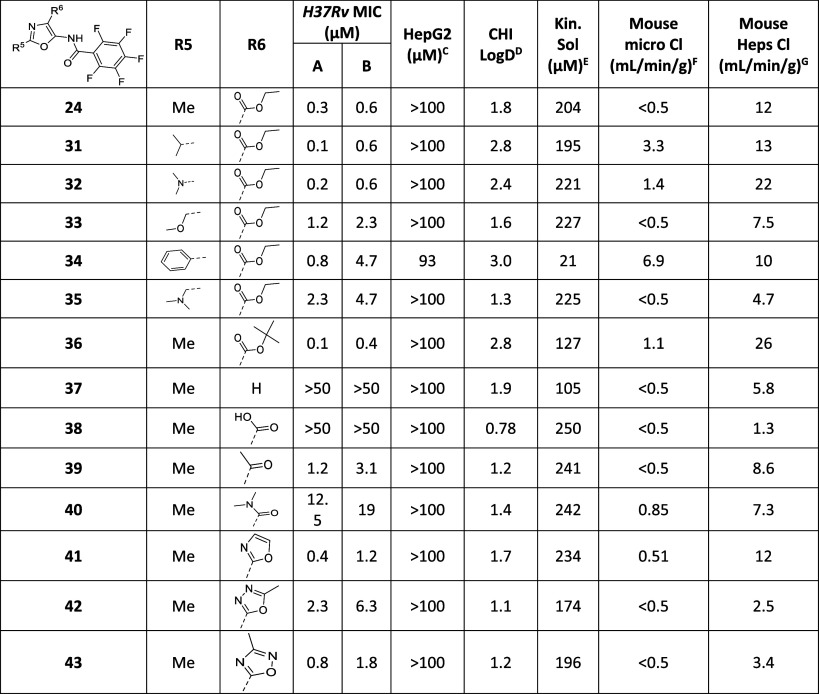
Expansion of SAR around the Oxazole Core

MIC required to inhibit the growth of *M. tuberculosis* (H37Rv) in liquid culture (^A^7H9/DPPC/CAS/Tx; ^B^7H9/DPPC/CHOL/BSA). ^C^HepG2 inhibitory concentration (IC_50_) is the concentration required to inhibit growth of HepG2 cells by 50%, ^D^CHI-LogD_pH7.4_ is a measure of lipophilicity at pH7.4; ^E^kinetic solubility in water; ^F^intrinsic clearance (Cli) using CD1 mouse liver microsomes; ^G^intrinsic clearance (Cli) using mouse hepatocytes.

Alongside the hypomorph experiments, strains resistant to **1** were raised. Resistant colonies were obtained on solid media containing **1** at a frequency of 10^–7^ at 5 times the MIC and 10^–8^ at 10 times the MIC of the compound. For the 12 resistant strains examined, each had a SNP in Pks13, and in each case, this was the only mutation identified by whole genome sequencing (Figure 2 and Table S1). Within the multidomain structure of Pks13, the mutations were found at 6 distinct residues and mapped to either the N-terminal acyl carrier protein (ACP) domain or the keto synthase (KS) domain (Figure 2). One change, F79L, was similar to the F79S mutation reported for **TP2** and **TP4**,^19^ two molecules from a thiophene series that also contain a pentafluorophenyl group and also target Pks13.

In MoA studies for the thiophene molecules, only the F79S-resistant mutation was reported. Computational modeling placed this mutation in close physical proximity to the P-pant (phosphopantetheine) attachment site (Ser55) in the N-ACP domain, leading to the suggestion that the thiophene series may interfere with the loading of the meromycolyl AMPs onto this key residue.^19^ Cellular analysis confirmed that **TP2** and **TP4** inhibited mycolic acid biosynthesis with the concomitant accumulation of mycolic acid precursors.^19^ Since resistant mutations to **1** covered a larger region of Pks13 than the one Phe79 residue, cross-resistance analysis was performed to see if these mutations also conferred resistance to **TP2** and **TP4** (Table S1). In all cases, the mutants were cross-resistant to both thiophenes (between 6- and 16-fold). The pentafluorophenyl group common to **1** and **TP2/4** has also been reported in the oncology drug T138067 (Figure S2) that progressed to Phase II clinical trials.^20,21^ The antineoplastic MoA of T138067 is through direct covalent interaction with β-tubulin at residue Cys239, thereby disrupting tubulin polymerization.^22^ The covalent reaction is via the para-position of the pentafluorophenyl moiety; its electrophilic nature is enhanced by the presence of a sulfonamide electron-withdrawing group adjacent to the warhead. Within the first 800 residues of Pks13, encompassing both the N-ACP domain and KS domain, there is one Cys residue (Cys287). This residue lies within the Cys-His-His active site motif (TA**C**SS **H**GTGT KTNVG**H**) of the KS domain and binds directly to one of the acyl chains prior to a Claisen-type condensation reaction to generate the α-alkyl β-ketoacyl chains, precursors of the mycolates.^23,24^ The four mutated residues found within the KS domain of the strains resistant to **1** all lie within the extended Cys-His-His active site (Figure 2). Thus, as well as potentially blocking P-Pant attachment at Ser55, **1** and **TP2/4** may bind directly to Cys287, thereby inhibiting the KS activity within Pks13.

Like T138067, the ability of **1** and **TP2/4** to form a direct covalent interaction is enhanced by the presence of an electron-withdrawing group (a thiazole in **1**; an amide in **TP2/4**) attached to the pentafluorophenyl warhead, making it more electrophilic. Given the potential covalent MoA, a glutathione conjugation assay was performed in human liver microsomes. As seen previously for T138067 and other compounds with the same warhead,^25−27^ there was clear evidence of a significant peak corresponding to the formation of the GSH adduct via the loss of a fluorine (Figure S3). This metabolite was seen as ± NADPH. Therefore, the electrophilic pentafluorophenyl warhead of **1** can react with thiol-based nucleophiles like glutathione and potentially cysteine residues in proteins.

Considering the reactive nature of **1**, it was not surprising to find that it had very poor metabolic stability in both mouse liver microsomes and mouse hepatocytes (Table 1). As such, the initial focus of the SAR was to try to improve metabolic stability while maintaining the antibacterial activity. Supporting the proposed covalent mechanism of action, modification of the pentafluorophenyl warhead was not tolerated (Table 1). Removal of the para-fluoro (**2**) completely eliminated activity, as did replacement with an unsubstituted phenyl (**3**). Elimination of both ortho-fluorines (**4**) along with retention of just the para-fluorine (**5**) was also inactive. Although inactive, all the modified compounds remained metabolically unstable in mouse microsomes (Table 1), thereby indicating that the thiazolo[5,4-*d*]pyrimidine core was also contributing to the molecule’s metabolic instability.

Consequently, modification of the core was assessed to try to reduce its metabolic liability. Replacement of the thiazole ring with an oxazole **6** retained activity, while changing to a *N*-methylimidazole **7** was detrimental to activity (Table 2). Substitution of the dimethylamine with either a diethylamino **8** or morpholino group (**9** and **10**) was tolerated (Table 2), but these molecules remained metabolically unstable. Removal of the dimethylamino group (**11**) led to a modest reduction in activity but was cytotoxic with human HepG2 cells. Replacement of the dimethylamino group with a NHMe (**12**) led to significant improvement in metabolic stability but unfortunately at the expense of MIC activity. Modification of the pyrimidine ring to a pyridine (**13** and **14**) showed no improvement in stability and a reduction in potency. Finally, while the 7-hydroxy analogue **15** maintained some MIC activity, it improved solubility and microsomal stability but remained very unstable in mouse liver hepatocytes (Table 2). Although the initial SAR suggested modifications could be made to **1**, the synthetic chemistry was challenging and not amenable to parallel synthesis, and none of the changes resulted in a significant improvement in the all-round properties of the molecule. As a result, the focus was expanded to include the **TP2** and **TP4** scaffolds (Table 3), which appeared to be working by the same mechanism, but the presence of an amide bond made the chemotypes more amenable to synthetic exploitation.

**TP2** and **TP4** were both very potent and had improved microsomal metabolic stability compared to **1**. However, both had poor solubility and some cytotoxicity toward HepG2 cells; both of these properties could be envisaged to be due in part to the central thiophene core which has been reported previously to represent a poorly developable core.^28,29^ Thus, the initial focus of the SAR was to explore core modifications to replace the thiophene moiety. Gratifyingly, all the thiophene replacements showed significantly improved solubility, consistent with the reduction in the lipophilicity/CHI LogD compared to **TP2** and **TP4** and improved selectivity against HepG2 cells (Table 3). Replacement of the thiophene core with a pyridyl group was tolerated, although the choice of pyridyl isomer was important. The 4-pyridyl isomer (**16**) showed similar MIC potency to **TP2/TP4**, while the 2-pyridyl (**17**) and 3-pyridyl (**18**) isomers were both significantly less active. A range of MIC potencies were also observed with alternative 5-membered heterocyclic replacements. The *N*-methylpyrazole (**19**) maintained potency, while the dimethylpyrazole (**20**) was inactive across all media. *N*-methylimidazole (**21**) displayed a 10-fold loss of potency and offered no improvement in metabolic stability. Methylisoxazole (**22**) showed activity similar to that of **21** but did have a modest improvement in microsomal metabolic stability. Methyloxazoles (**23 and 24**) both showed similar activity to **TP2 and TP4** and had improved metabolic stability in mouse liver microsomes (Table 3), although the hepatocyte metabolic stability remained above the minimum requirement for in vivo investigation (<5 mL/min/g). Before further SAR was initiated, it was confirmed that **TP2** and **24** still inhibited *M. tuberculosis* growth, by targeting Pks13, using the resistant strains and the hypomorph strain (Figure S4 and Table S1).

Further exploration of the SAR continued, based on molecules with an oxazole core. Systematic modification of each vector of **24** was performed to improve the hepatocyte metabolic stability while maintaining other favorable properties. Starting with the pentafluorophenyl warhead, removal of the ortho-fluorine to give **25** maintained potency but led to an increase in metabolic instability (Table 4). Elimination of both ortho fluorines (**26**) completely abolished the MIC activity. Deletion of *meta*-fluorine (**27**) led to an improvement in hepatocyte stability but at the expense of MIC potency. Deletion of the *para*-fluorine (**28**) led to a complete loss of growth inhibition, as would be expected if the molecule had a covalent MoA. Modifications to the benzamide linker were not tolerated with both the benzylamine (**29**) or sulfonamide (**30**) being inactive. Since a sulfonamide linker is tolerated in T138067,^22^ the latter result suggested that the sulfonamide prevents the pentafluorophenyl warhead from adopting a suitable conformation to interact with Pks13 rather than elimination of its reactivity.

As the pentafluorophenyl group could not tolerate modification, the next point of SAR evaluation was substitutions off the oxazole core. Initially, replacement of 2-methyl was explored (Table 5); introduction of either an isopropyl (**31**) or dimethylamine (**32**) maintained potency and microsomal stability but gave no benefit to hepatocyte metabolic stability. Changing the methyl to a methoxymethyl (**33**), phenyl (**34**), or dimethylamino-methyl (**35**) all resulted in a small reduction in MIC activity and did not improve hepatocyte metabolic stability significantly. The final focus of SAR was around the ethyl ester at position 4 of the oxazole (Table 5). Esters are known to be a site for potential metabolic liability. Potency was maintained when the ethyl ester was modified to either a methyl ester (**23**) or a *t*-butyl ester (**36**) but neither offered an improvement in metabolic stability. Complete removal of the ester (**37**) led to a total loss of MIC activity as did conversion into a carboxylic acid (**38**). Replacement of the ester with either a methyl ketone (**39**) or a dimethyl amide (**40**) led to a reduction in MIC activity; neither compound improved the metabolic stability (Table 5). Heterocyclic isosteres were also investigated as ester replacements; the oxazole (**41**) maintained similar properties to **24**, while oxadiazoles (**42** and **43**) showed a significant improvement in hepatocyte metabolic stability, to the extent that they met the criteria required for an early lead (<5 mL/min/g). The 1,3,4-oxadiazole (**42**) showed a 10-fold loss in MIC activity, but the 1,2,4-oxadiazole (**43**) had only a modest reduction in potency. As such, **43** was selected for further follow-up as a potential early lead compound for this series.

Although **43** had improved metabolic stability in both microsomes and hepatocytes, it was important to demonstrate that the reactivity with glutathione, seen for **1**, had also been reduced, otherwise in vivo the compound would disappear rapidly. A comparative metabolite identification time course study was performed with both **43** and **TP2** in human liver microsomes. While levels of **43** did slowly decrease, due to the appearance of defluorinated glutathione conjugates, after 90 min, 57% of the parental material remained (Figure S5). In contrast, **TP2** was eliminated completely within 6 min due to glutathione conjugation (Figure S5). Thus, the improvements in metabolic stability translated into decreased reactivity with glutathione, and therefore, **43** was considered a suitable candidate for in vivo evaluation. An initial pharmacokinetic study was performed to determine whether the exposure to **43** was suitable for an assessment of efficacy. The in vivo exposure, bioavailability, and clearance were all very good, the half-life was moderate, and the volume of distribution was limited (Figure S6); the compound was considered suitable for evaluation in an acute TB murine infection model.

**Figure 3:**
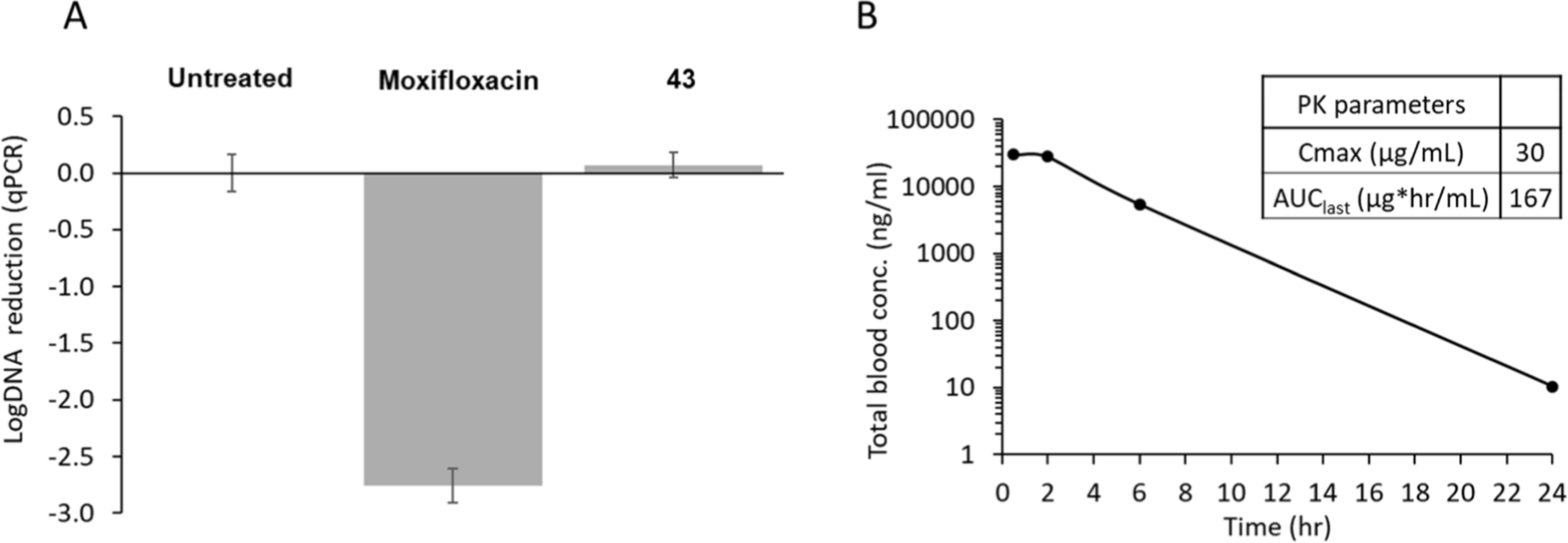
In vivo efficacy assessment of 43 in the murine model of acute TB infection. C57BL/6J mice were infected with *M. tuberculosis* H37Rv and were dosed orally for 8 days starting on the first day post-infection. Mice were sacrificed at least 24 h after the last dose. (A) Impact of treatment on bacterial load as assessed by monitoring bacterial DNA in lung homogenates using qPCR. (B) Total blood concentrations taken at four time points on the first day of dosing from individual infected mice.

In this efficacy study (Figure 3), compounds were dosed for 8 days starting 1 day post-infection. At the end of the dosing period, the lungs were harvested, and the bacterial load was assessed by monitoring the levels of bacterial DNA using qPCR. Moxifloxacin was used as a control compound and gave the expected ∼3 log reduction in DNA compared to the untreated control. Unfortunately, **43** showed no reduction in DNA levels compared to the untreated control, indicating **43** had no efficacy in this model. Originally, evaluation of cidality and intramacrophage activity had not been explored because work with two other series of Pks13 inhibitors had validated it as a good intracellular target.^13,15^ However, given the lack of in vivo efficacy, a more in-depth analysis of the bactericidal efficacy of **43** was performed (Figure S7). The compound had good bactericidal activity with a CFU reduction of ∼1 or 2 log units, relative to input bacterial counts, following treatment for 3 or 7 days, respectively (Figure S7A). Unexpectedly, although **43** did have activity against H37Rv growing inside J774 macrophages, it required 7 days of treatment at relatively high concentrations (10 μM) to exert a cidal effect (Figure S7B). This was unexpected because two earlier series had shown good intramacrophage activity, suggesting that the efficacy of **43** was limited by penetration into macrophages and/or the phagosomal environment and not by target vulnerability during host pathogenesis. As the bacteria within this acute model are mostly intracellular, this would suggest that a higher exposure to the compound than initially anticipated would be required to inhibit bacterial growth. In the efficacy study, spot PK samples were taken on the first day of dosing; although the efficacy experiment was performed at 20 times the oral PK dose, this was not reflected in the exposure (*C*_max_ was <3-fold increased in the animal efficacy study). As a result, although **43** was present for 24 h, given that it is 97% plasma protein bound, the free concentration of the compound would not have been above the levels required to kill the bacteria for suitable exposure periods.

## Conclusions

Phenotypic screening of a large library of ∼225,000 compounds identified a potent inhibitor of *M. tuberculosis* growth **1**. Mechanism-of-action studies demonstrated that **1** targeted Pks13, an essential and validated drug target involved in cell-wall biosynthesis.^15,30^ The presence of a pentafluorophenyl moiety within the structure of **1** bore a clear resemblance to the literature molecules **TP2** and **TP4** that have also been shown to target Pks13.^19^ The metabolic stability of **1** was poor, and standard medicinal chemistry optimization was unable to improve it. Synthetic progress was also impacted by the challenging chemistry required to make modifications. To address this, expansion of the program to include **TP2** and **TP4** as starting points was able to identify **43**, a compound that was potent against *M. tuberculosis* and importantly metabolically stable enough to be suitable for in vivo evaluation. Unfortunately, **43** showed no efficacy in an acute model of TB infection, which was most likely due to insufficient coverage of the compound above the levels required to kill the bacteria in this model. To obtain **43** had been a significant challenge, modulating the reactivity of the pentafluorophenyl warhead within the molecule so that it could still target Pks13 while having suitable metabolic stability to explore in vivo. There is an urgent need for novel TB drugs, and our work combined with other studies on Pks13 inhibitors has demonstrated the vulnerability of Pks13 in *M. tuberculosis* in vitro and in vivo, highlighting the potential promise of this scaffold for further development. Additional work will be required to develop this series further which will involve balancing the issues of drug-like properties, MIC potency, and the translation into a compound with improved macrophage penetration and in vivo efficacy.

## Methods

### Chemistry

#### Synthetic Routes

**Scheme 1:**
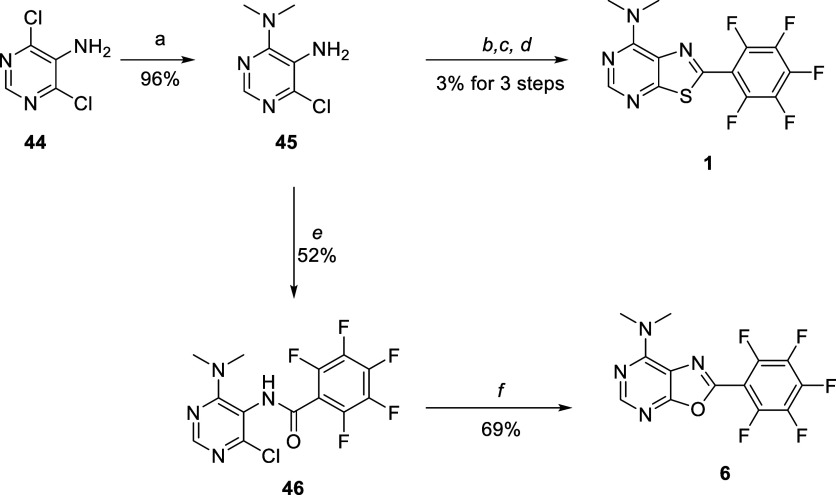


The synthesis of thiazolopyrimidine **1** is shown in Scheme 1. 5-Amino-4,6-dichloropyrimidine **44** was treated with dimethylamine to give monoamine adduct **45** which was converted in 3 steps to **1** using standard chemistry. The analogous oxazole **6** was prepared in 2 steps starting from **45**. Acylation of **45** with pentafluorobenzoyl chloride gave benzamide **46** which was cyclized to oxazole **6** upon treatment with polyphosphoric acid.

Thiazolopyrimidines (**2, 3, 4, 5, 81, 9, and 11**), imidazolopyrimidine **7**, and oxazolopyrimidines (**6, 12, 13, 14**, **and 15**) were synthesized using similar chemistry (Schemes S1?S4).

Heteroaryl fluorobenzamides (**16, 17, 18, 19, 20, 21, 22, 31, 32, 33,** and **34**) were prepared by using standard conditions (Schemes S5?S7) by acylation of the appropriate amine and pentafluorobenzoyl chloride. Amino-oxazole **47** proved to be a key building block for the synthesis of both **24** and **43** (Scheme 2) as well as fluorinated benzamides (**25, 26, 27, 28,** and **35**), benzylamine **29, s**ulfonamide **30**, and ester isosteres (**23, 36, 37, 38, 39, 40, 41, and 42**) (Schemes S8–S18).

**Scheme 2:**
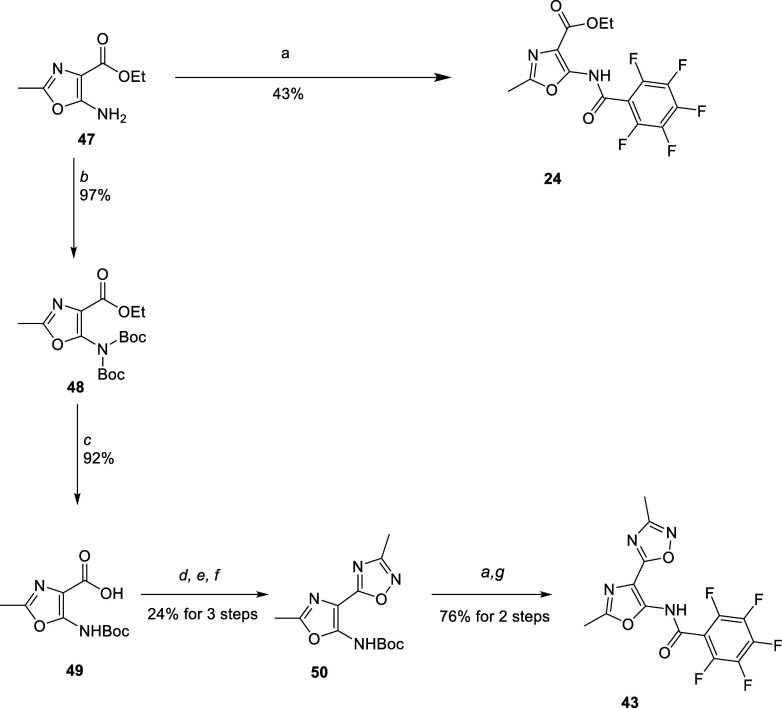


#### Compound Synthesis

Full experimental procedures can be found in the Supporting Information section.

### Non-chemistry Methods

#### Screening

*M. tuberculosis* H37Rv or derivatives thereof were used for all experiments. Single-point (20 μM) high-throughput screening was performed on an ∼225,000 compound library provided by Medicines for Malaria Venture (MMV6). HTS was performed on two liquid media 7H9/Glu/BSA/Tx (4.7 g/L Middlebrook 7H9 broth base, 4 g/L glucose, 0.8 g/L NaCl, 5 g/L BSA fraction V, and 0.05% Tyloxapol) and DPPC/Chol/BSA (4.7 g/L Middlebrook 7H9, 6 μM DPPC, 62.5 μM cholesterol, 0.8 g/L NaCl, 5 g/L BSA fraction V, and 0.05% Tyloxapol) for 3 and 4 days, respectively. The strain used was transformed with a GFP encoding vector and phenotypic growth inhibition was monitored by loss of fluorescence compared to the untreated controls.^31^ Compounds that inhibited growth >50% were reconfirmed in an equivalent assay but over a dose–response curve rather than at a single compound concentration.

#### Minimal Inhibitory Concentration

MIC measurements were performed as previously described,^32^ and multiple media were used, with this report highlighting the results from DPPC/Chol/BSA and DPPC/Cas/Tx (4.7 g/L Middlebrook 7H9, 6 μM DPPC, 0.8 g/L NaCl, 0.03% casitone, and 0.05% tyloxapol). Results for growth on two additional media 7H9/Glu/BSA/Tx and 7H9/Glu/Cas/Tx (4.7 g/L Middlebrook 7H9 broth base, 4 g/L glucose, 0.8 g/L NaCl, 0.03% casitone, and 0.05% tyloxapol) are included in the Data and PAINS summary file in the Supporting Information. The MICs were determined by visual inspection of the microtiter plates after 1 or 2 weeks of growth at 37 °C using an enlarging inverted mirror.

#### Mutant Generation

To raise resistant mutants against **1**, H37Rv cells (10^7^, 10^8^, and 10^9^) were plated on 7H11/OADC plates containing 5 times or 10 times the in vitro MIC of **1**. Drug-free plates were used to enumerate the bacterial load. The plates were incubated at 37 °C for 4–6 weeks until colonies grew to an appreciable size. To confirm resistance against **1**, colonies were established on a drug-free medium and the MICs were determined. The genomic DNA of the mutants was isolated using a CTAB method and sequenced as previously described.^33^

#### Whole Genome Sequencing and Determination of Resistance Mutations

Genomic DNA samples were prepared for sequencing using TruSeq DNA sample preparation kits (Illumina, Inc.) and sequenced on an Illumina Rapid Hiseq 2500 instrument in paired-end mode with a read length of 125 + 125 bp and a mean depth of coverage of 93.4×. Sequencing reads were mapped to the *M. tuberculosis* H37Rv reference genome using BWA.^34^ A custom script was used to extract genetic variants (SNPs and indels) by comparative analysis with the parental genome sequence, applying standard filters to exclude sites with low coverage (<10×) or heterogeneous base calls (<70% purity).

#### MIC Comparison in Cell Wall Hypomorphs

MIC measurements for the hypomorph strains were performed as described previously.^13−15^ The Pks13 hypomorph strain grew similarly ±atc, indicating that the different Pks13 expression levels had no impact on cell growth directly (Figure S4).

#### ADME/PK Analysis

Assays to establish HepG2 cytotoxicity, in vitro microsomal/hepatocyte metabolic stability, aqueous kinetic solubility, and in vivo pharmacokinetic profiles were all performed as previously described.^15,35−37^ All regulated procedures, at the University of Dundee, on living animals were carried out under the authority of a project license issued by the Home Office under the Animals (Scientific Procedures) Act 1986, as amended in 2012 (and in compliance with EU Directive EU/2010/63). License applications will have been approved by the University’s Ethical Review Committee (ERC) before submission to the Home Office. The ERC has a general remit to develop and oversee policy on all aspects of the use of animals on university premises and is a subcommittee of the University Court, its highest governing body.

#### Efficacy

All procedures were performed in accordance with protocols approved by the GSK Institutional Animal Care and Use Committee and met or exceeded the standards of the American Association for Accreditation of Laboratory Animal Care (AAALAC). All animal studies were ethically reviewed and carried out in accordance with European Directive 2010/63/EEC and the GSK Policy on the Care, Welfare, and Treatment of Animals. Acute studies were performed as described.^38^

## Supporting Information Available

Resistant mutants isolated for **1** and cross-resistance with other agents from the series; PiniB-LUX reporter data for H37Rv treated with compound **1** and controls; antineoplastic agent T138067; metabolism of **1** in human liver microsomes; validation of the pks13-TetON strain and further evaluation of 3 additional inhibitors from this series and rifampicin; time course of loss of parent molecule in liver microsomes for **TP2** and **43**; murine pharmacokinetic profile for **43**; in vitro bactericidal and intramacrophage activity for **43**; chemistry and experimental methods for all molecules and synthetic schemes; and ^1^H NMR and HRMS for compounds **1**, **6**, **24**, and **43** (PDF)
In vitro data table with PAINS alert analysis and SMILES (XLSX)

## Abbreviations

Eq: equivalents
TEA: triethylamine
HATU: 1-[bis(dimethylamino)methylene]-1H-1,2,3-triazolo[4,5-*b*]pyridinium 3-oxide hexafluorophosphate
EA: ethyl acetate
DMF: *N*,*N*-dimethylformamide
Sat: saturated
Aq: aqueous
THF: tetrahydrofuran
DCM: dichloromethane
TFA: trifluoroacetic acid
Boc: *tert*-butoxycarbonyl
DMAP: 4-dimethylaminopyridine
DIPEA: diisopropylethylamine
CDI: 1,1′-carbonyldiimidazole
NBS: N-bromosuccinimide
BINAP: 2,2′-bis(diphenylphosphino)-1,1′-binaphthyl
dba: dibenzylideneacetone
AIBN: 2,2′-azobis(2-methylpropionitrile)
EDCI: *N*-ethyl-*N*′-(3-(dimethylamino)propyl)carbodiimide hydrochloride
HOBt: *N*-hydroxybenzotriazole
DPPC: dipalmitoylphosphatidylcholine
OADC: oleic albumin dextrose catalase
TB: tuberculosis
ND: not done.
