# Supplementary material for: Identification of a Series Containing a Pentafluorophenyl Moiety That Targets Pks13 to Inhibit Growth of Mycobacterium tuberculosis
Source: ACS Infect Dis. 2025 Feb 27;11(3):715–26. doi: 10.1021/acsinfecdis.4c00808 (PMC11915372; doi:10.1021/acsinfecdis.4c00808)
Supplement: Supplementary file 1 — id4c00808_si_001.pdf [file id4c00808_si_001.pdf]

Supplemental Information

Identification of a series containing a pentafluorophenyl moiety that targets Pks13 to inhibit growth of *Mycobacterium tuberculosis*

Simon R Green[‡]*, Justin R Harrison[‡], Stephen Thompson[‡], Dinakaran Murugesan[‡], M Daben J. Libardo[ϯ], Curtis A Engelhart[¥], Jaclynn Meshanni[¥], Daniel Fletcher[‡], Paul Scullion[‡], Darren Edwards[‡], Ola Epemolu[‡], Nicole Mutter[‡], Yoko Shishikura[‡], Jennifer Riley[‡], Thomas R Ioerger[≠], Jose Juan Roca Guillén[ɋ], Laura Guijarro López[ɋ], Kevin D Read[‡], Clifton E Barry 3[rdϯ], Dirk Schnappinger[¥], Paul G Wyatt[‡], Helena I M Boshoff[ϯ], and Laura A T Cleghorn[‡]*

[‡]Drug Discovery Unit, Division of Biological Chemistry and Drug Discovery, School of Life Sciences, University of Dundee, Dundee, DD1 5EH, UK

[ϯ]Tuberculosis Research Section, Laboratory of Clinical Immunology and Microbiology, NIAID, NIH, 9000 Rockville Pike, Bethesda, Maryland, USA

[¥]Department of Microbiology and Immunology, Weill Cornell Medical College, New York, NY, 10065, USA

[≠]Department of Computer Science and Engineering, Texas A&M University, College Station, TX 77843, USA

[ɋ]Global Health Medicines R&D, GlaxoSmithKline, Severo Ochoa 2, Tres Cantos, 28760, Madrid, Spain

***Corresponding Author Information:**

S.R.Green@dundee.ac.uk

l.a.t.cleghorn@dundee.ac.uk

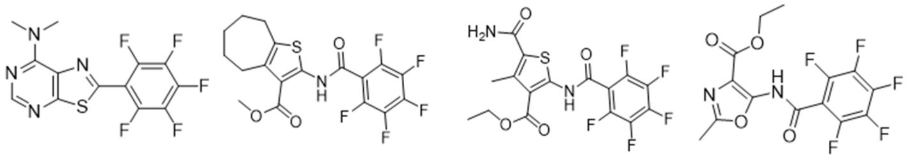

	1	TP2	TP4	24
**MIC H37Rv (µM)**	0.8	1. 6	1.2	0.8
**MIC P47R (µM)**	6.3	12.5	6.3	-
**MIC E436Q(µM)**	3.1	9.4	6.3	3.1
**MIC A437V(µM)**	3.1	6.3	6.3	-
**MIC N395D(µM)**	6.3	12.5	6.3	-
**MIC F79L(µM)**	12.5	25	6.3	25
**MIC E436K(µM)**	12.5	25	6.3	-
**MIC S388F(µM)**	12.5	25	6.3	-

Table S1: Resistant Mutant data for 1, TP2/TP4 and 24

MIC required to inhibit the growth of *M. tuberculosis* in liquid culture (7H9/GLU/GLY/BSA/Tween). The MIC is shown for wild type H37Rv and each of the seven strains resistant to 1; - indicates not done.

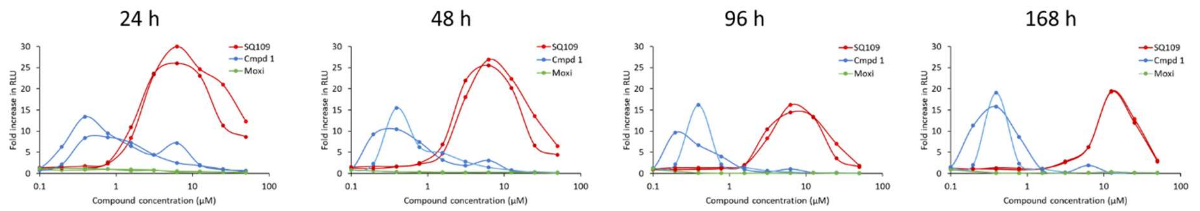

Figure S1. Compound 1 activates the PiniB-LUX reporter indicating a cell wall biosynthetic pathway mechanism of action.

H37Rv strain transfected with the Pini-LUX reporter vector was treated with compound as previously reported{Naran, 2016 #265}. Luminescence was monitored at 4 different timepoints. The fold increase in luminesce relative to a DMSO control is shown for the strain treated with **1**, SQ109 (positive control) and moxifloxacin (negative control). Experiments were performed in duplicate.

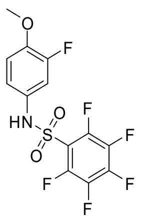

Figure S2: **The antineoplastic agent T138067**

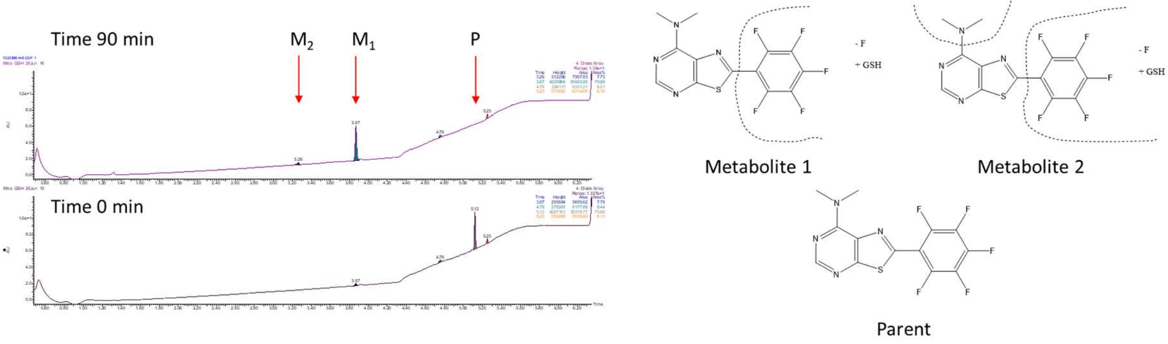

Figure S3: **Metabolism in human liver microsomes in the presence of glutathione and NADPH**

UV Chromatograms of sample at t=0 (lower chromatogram) and t=90 (upper chromatogram) – peak corresponding to parent (P) and metabolites (M_1 & M_2) shown with arrows along with the proposed structures of M_1 & M_2 as determined by msms fragment analysis.

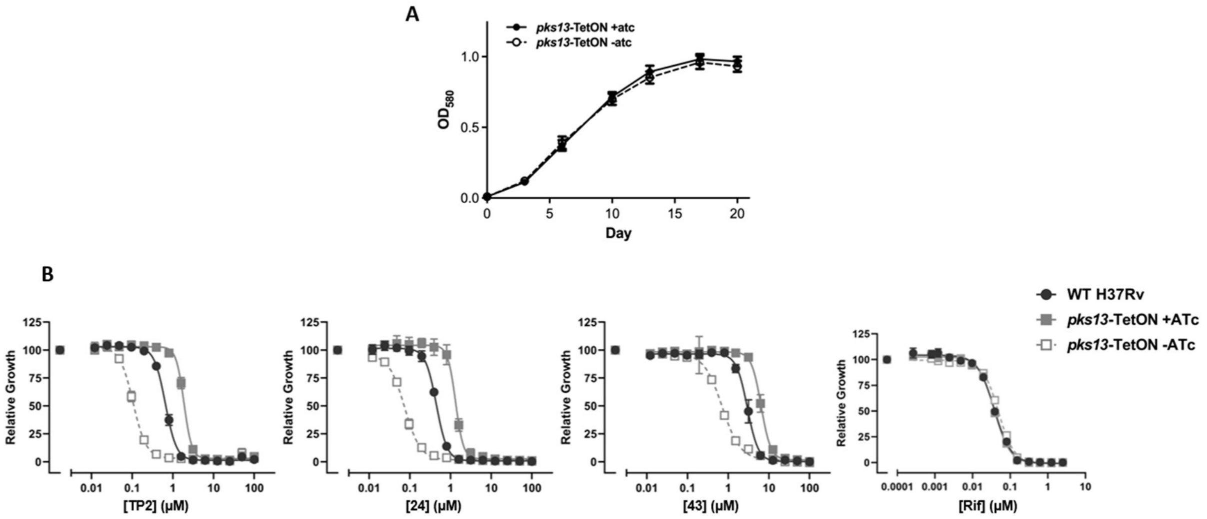

Figure S4. Validation of the pks13-TetON strain and further evaluation of 3 additional inhibitors from this series and rifampicin

A. WT H37Rv and pks13-TetON (which produces about four times the WT Pks13 level when grown with anhydrotetracycline [ATc] and one-fifth of WT Pks13 when grown without ATc) have been described previously (Wilson et al 2022, Green et al 2023 & Krieger et al 2024 references in main manuscript). Growth of the pks13 TetON strain was monitored by optical density at 580nm for 20 days ±atc. Changes in the level of Pks13 protein in the pk13 TetON strain, had no impact on growth rate. **B.** Removal of anhydrotetracycline (atc) results in transcriptional repression of Pks13 gene expression. Growth in the presence of each compound relative to a DMSO control is shown for H37Rv and each hypomorph strain ±atc. Differences in the growth of the pks13 hypomorph are evident for the Pks13 inhibitors but not for the control compound rifampicin, as reported previously (Wilson et al 2022, Green et al 2023 & Krieger et al 2024 references in main manuscript). Data are representative of two independent experiments.

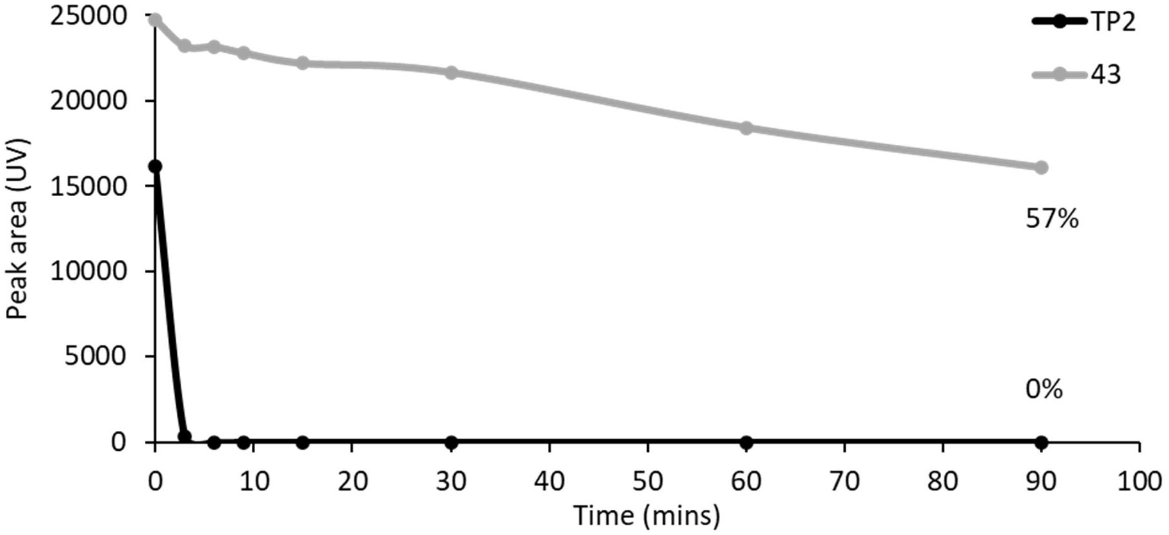

Figure S5 Metabolite analysis monitoring loss of parent with time when incubated with human microsomes and glutathione/NADPH

The change in levels of the parent compound (UV peak area) was monitored over time when either **43 or TP2** was incubated in pooled human liver microsomes in the presence of both glutathione and NADPH. Samples were taken at the indicated times and assessed by mass spectrometry. The peak area was determined from UV chromatograms and determined for both parent and potential glutathione conjugated metabolites.

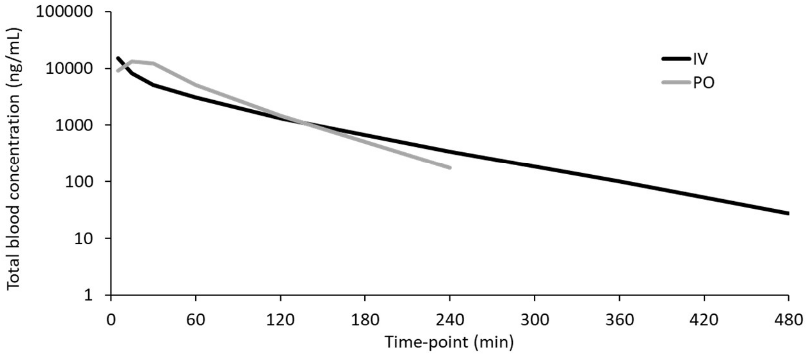

PK parameters	43	
	IV	PO
Cmax (µg/mL)		13
Tmax (min)		15
T1/2 (h)	1.1	
AUC0-480 (µg*hr/mL)	11	14.8
Clb (mL/min/kg)	4.6	
Vdss (L/kg)	0.31	
F (%)		42

Figure S6: *In vivo* pharmacokinetic profile for 43 in mice

Mean total blood concentrations of **43** following dosing with 10 mg/kg po or 3 mg/kg iv to female C57BL/6J mice (n=3/dose). Blood samples were taken from tail vein at predetermined time points post-dose, mixed with two volumes of distilled water and stored frozen until UPLC/MS/MS analysis.

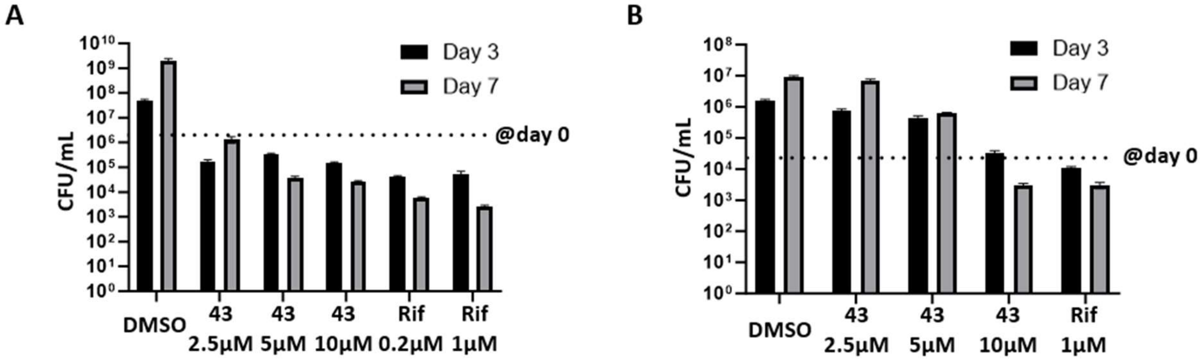

Figure S7: *In vitro* bactericidal and intramacrophage activity for 43

A. Cidality was assessed by growing H37Rv in the presence of compound for either 3 or 7 days followed by viable colony enumeration on 7H11/OADC media. **B.** Activity against H37Rv growing inside J774 macrophages was assessed by treating *M. tuberculosis*-infected J774A.1 macrophages with compound for either 3 or 7 days. The number of viable bacteria post treatment was determined by plating dilutions on 7H11/OADC media. Data are representative of three replica experiments analysed in duplicate.

General chemistry methods. Chemicals and solvents were purchased from commercial vendors and were used as received, unless otherwise stated. Dry solvents were purchased in septum-capped bottles. Analytical thin-layer chromatography (TLC) was performed on precoated TLC plates (Kieselgel 60 F254, BDH). Developed plates were air-dried and analysed under a UV lamp (UV 254/365 nm) and/or $KMnO_4$ was used for visualization. Flash chromatography was performed using Combiflash Companion Rf (Teledyne ISCO) and prepacked silica gel columns purchased from Teledyne ISCO. Mass-directed preparative HPLC separations were performed using Waters HPLC systems (2545 binary gradient pumps, 515 HPLC make-up pump, 2767 sample manager) connected to a Waters 2998 photodiode array and a Waters 3100 mass detector or a Waters Acquity QDa mass detector. Preparative HPLC separations were performed with a Gilson HPLC (321 pumps, 819 injection module, 215 liquid handler/injector) connected to a Gilson 155 UV/vis detector. On both instruments, HPLC chromatographic separations were conducted using Waters XBridge C18 columns, 19 mm × 100 mm, 5 µm particle size, using 0.1% ammonia in water (solvent A) and acetonitrile (solvent B) as mobile phase. ^{1}H NMR spectra were recorded on a Bruker AVANCE II 500 or 400, Bruker AVANCE NEO 400, AVANCE III 400 or AVANCE III 400 HD spectrometer using $CDCl_3$, DMSO-d_6, acetone-d_6 or CD_3OD solutions. Chemical shifts (δ) are expressed in ppm recorded using the residual solvent as the internal reference in all cases. Signal splitting patterns are described as singlet (s), doublet (d), triplet (t), multiplet (m), broadened (br) or a combination thereof. Coupling constants (*J*) are quoted to the nearest 0.1 Hertz (Hz). Low resolution electrospray (ES) mass spectra were recorded on a Bruker Daltonics MicrOTOF mass spectrometer run in positive or negative mode. High resolution mass spectroscopy (HRMS) was performed using a Bruker Daltonics MicroTof mass spectrometer. LC−MS analysis and chromatographic separation were conducted with either a Bruker Daltonics MicrOTOF mass spectrometer connected to an Agilent diode array detector, Thermo Dionex Ultimate 3000 RSLC system with diode array detector, the column used was a Waters XBridge column (50 mm × 2.1 mm, 3.5 µm particle size), and the compounds were eluted with a gradient of 5−95% acetonitrile/water + 0.1% ammonia, or with an Agilent Technologies 1200 series HPLC connected to an Agilent Technologies 6130 quadrupole LC/ MS, connected to an Agilent diode array detector, the column used was a Waters XBridge column (50 mm × 2.1 mm, 3.5 µm particle size) or a Waters X-select column (30 mm x 2.1mm, 2.5 µm particle size) with a gradient of 5-90% acetonitrile/water + 0.1% formic acid, or with an Advion Expression Mass Spectrometer connected to a Thermo Dionex Ultimate 3000 HPLC with diode array detector, the column used was Waters XBridge column (50 mm × 2.1 mm, 3.5 µm particle size) or a Waters X-select column (30 mm x 2.1mm, 2.5 µm particle size) with a gradient of 5-90% acetonitrile/water + 0.1% formic acid. Alternatively, compounds were analysed by WuXi AppTec using SHIMADZU *m/z*-2020, Agilent G1956A or Agilent G6110A LC-MS systems, using a Kinetex EVO column (C18 30 mm x 2.1 mm column, 5 µm particle size) with a gradient of 5-95% mobile phase A/mobile phase B (acidic conditions: mobile phase A: 0.0375% TFA in water, v/v; mobile phase B: 0.01875% TFA in acetonitrile, v/v; basic conditions: mobile phase A: 0.025% ammonia in water, v/v; mobile phase B: acetonitrile). All final compounds showed chemical purity of ≥95% as determined by the UV chromatogram (190−450 nm) obtained by LC−MS analysis. Microwave-assisted chemistry was performed using a CEM or a Biotage microwave synthesizer.

6-Chloro-N^4,N^4-dimethylpyrimidine-4,5-diamine (45)

A solution of 4,6-dichloro-5-pyrimidinamine (1.00 g, 6.10 mmol) and dimethylamine, 2 M solution in THF (4.9 mL, 9.8 mmol) in 2-propanol (1 mL) was treated with triethylamine (2.5 mL, 18 mmol). The mixture was stirred at room temperature. After 27 hours, TLC showed complete reaction. The mixture was diluted with diethyl ether (20 mL) and filtered. The filtrate was concentrated to give the *title* compound as a brown solid (1.095 g, 5.84 mmol, 96% yield). ^{1}H NMR (400 MHz, CDCl3) δ 8.12 (s, 1H), 3.81 (br.s, 2H), 2.97 (s, 6H). LRMS (ESI) (*m/z*) $[MH]^+$ = 173.1

6,6'-disulfanediylbis(N^4,N^4)-dimethylpyrimidine-4,5-diamine

To a solution of 6-chloro-N^4,N^4-dimethylpyrimidine-4,5-diamine (1 g, 5.79 mmol, 1 eq) in DMF (10 mL) was added sodium monosulfide (678 mg, 8.69 mmol) and the reaction heated at 80 °C for 3 hours. The reaction mixture was concentrated *in vacuo* to give a crude residue. Purification by column chromatography (SiO_2, PE:EA = 9:1-0:1) gave the *title* compound as a white solid (430 mg, 1.16 mmol, 20% yield) which was used without further purification. ^{1}H NMR (400MHz, DMSO-d_6): δ 7.65 - 7.55 (m, 2H), 4.56 - 4.30 (m, 4H), 2.67 (s, 12H). LRMS (ESI) (*m/z*) $[MH]^+$ = 339.1

***N,N*-dimethyl-2-(perfluorophenyl)thiazolo[5,4-d]pyrimidin-7-amine hydrochloride (1)**

To a mixture of 6,6'-disulfanediylbis(*N^4,N^4*)-dimethylpyrimidine-4,5-diamine (300 mg, 0.89mmol) *eq*) in acetic acid (3 mL) was added zinc powder (174 mg, 2.66 mmol) in one portion at 20 °C under N_2 and heated at 80 °C 2 hours. The mixture was then cooled to 10 °C and filtered to remove the solid. Pentafluorobenzoyl chloride (306 mg, 1.33 mmol) was added into the filtrate at 10 °C under N_2 and the mixture was stirred at 100 °C for 24 hours. The reaction was filtered and the filtrate was concentrated *in vacuo* to give a crude residue which was purified by prep-HPLC (column: Phenomenex Synergi C18 150*25*10 um; mobile phase: [water (0.05% HCl)-ACN]; B%: 25%-45%, 11min) to give the *title* compound as a white solid (54.2 mg, 0.154 mmol, 17% yield). ^{1}H NMR (400MHz, CDCl3): δ 8.48 (s, 1H), 3.62 (br s, 6H). HRMS (ESI): m/z calcd for $C_{13}H_8F_5N_4S$ $[M+H^+]$: 347.0384. Found 347.0390

***N*-(4-chloro-6-(dimethylamino)pyrimidin-5-yl)-2,3,4,5,6-pentafluorobenzamide (46)**

To a stirred solution of 6-Chloro-N^4,N^4-dimethylpyrimidine-4,5-diamine (496 mg, 2.82 mmol) in DCM (5 mL) and Pyridine (500 µL) at 0 °C was added pentafluorobenzoyl chloride (0.49 mL, 3.4 mmol, 1.2 eq.) and the reaction was stirred at room temperature overnight. The reaction mixture was diluted with DCM, washed (sat. aq. $NaHCO_3$, then sat. aq. NH_4Cl) and concentrated. The residue was resuspended in heptane and concentrated to remove pyridine residues. Purification by column chromatography (SiO_2, Heptane:EtOAc 1:0-2:3) gave the *title compound* as a red-brown solid (567 mg, 1.47 mmol, 52% yield). ^{1}H NMR (500 MHz, DMSO) δ 10.77 (s, 1H), 8.32 (s, 1H), 3.18 (s, 6H). LRMS (ESI) (m/z) [MH]+ =366.9

***N,N*-dimethyl-2-(pentafluorophenyl)oxazolo[5,4-*d*]pyrimidin-7-amine (6)**

N-(4-chloro-6-(dimethylamino)pyrimidin-5-yl)-2,3,4,5,6-pentafluorobenzamide (50 mg, 0.14 mmol) was treated with polyphosphoric acid (0.5 mL) and stirred at 140 °C for five hours and then at 130 °C overnight. The reaction mixture was cooled to 95 °C, diluted with water (ca. 2 mL), stirred to give a solution then cooled on ice. The solution was neutralized to pH 7 with 1 M aqueous NaOH. The resulting off-white solid was collected by filtration, washed with water and dried *iv* to give the *title compound* as a pale grey solid (32 mg, 95 µmol, 69% yield). ^{1}H NMR (500 MHz, DMSO) δ 8.41 (s, 1H), 3.66 (br. s, 3H), 3.29 (br. s, 3H). HRMS (ESI): m/z calcd for $C_{13}H_8F_5N_4O$ $[M+H^+]$: 331.0613. Found 331.0601

Ethyl 2-methyl-5-(pentafluorobenzamido)oxazole-4-carboxylate (24)

To a solution of ethyl 5-amino-2-methyloxazole-4-carboxylate (500 mg, 2.94 mmol, 1 *eq.*) and 2,3,4,5,6-pentafluorobenzoyl chloride (0.45 mL, 3.23 mmol, 1.1 *eq.*) in DCM (5 mL) was added TEA (1.23 mL, 8.81 mmol, 3 *eq.*) at 0 °C and the reaction mixture was stirred at 25 °C for 12 hours. The mixture was concentrated under vacuum. The residue was purified by column chromatography (SiO_2, SiO_2, PE: EA = 20:1 -5:1) to give the *title* compound (460 mg, 1.26 mmol, 43% yield) as white solid. ^{1}H NMR (500 MHz, DMSO) ⍰ 11.70 (s, 1H), 4.24 (q, *J*=7.1 Hz, 2H), 2.45 (s, 3H), 1.25 (t, *J*=7.1 Hz, 3H). HRMS (ESI): m/z calcd for $C_{14}H_{10}F_5N_2O_4$ $[M+H^+]$: 365.0555. Found 365.0562

ethyl 5-(bis(*tert*-butoxycarbonyl)amino)-2-methyloxazole-4-carboxylate (48)

The reaction mixture of ethyl 5-amino-2-methyloxazole-4-carboxylate (10 g, 58.8 mmol, 1 eq.), Boc_2O (38.48 g, 176 mmol, 3 eq.), DMAP (1.44 g, 11.75 mmol, 0.2 eq.) and DIPEA (51 mL, 0.29 mol, 5 eq.) in acetonitrile (100 mL) was stirred at 25 °C for 12 hrs. LCMS showed the reaction was complete. The reaction mixture was concentrated in vacuum. The residue was purified by column chromatography (SiO_2, PE: EA = 50:1 - 10:1) to give the *title* compound (21 g, 56.7 mmol, 97% yield) as an off-white solid. ^1H NMR (400 MHz, $CDCl_3$) δ 4.35 (q, *J*=7.2 Hz, 2H), 2.49 (s, 3H), 1.45 (s, 18H), 1.35 (t, *J*=7.2 Hz, 3H). LRMS (ESI) (m/z) [MH]+ = 371.0

5-((*tert*-butoxycarbonyl)amino)-2-methyloxazole-4-carboxylic acid (49)

The reaction mixture of ethyl 5-(bis(*tert*-butoxycarbonyl)amino)-2-methyloxazole-4-carboxylate (5 g, 13.5 mmol, 1 eq.) and $LiOH.H_2O$ (2.27 g, 54 mmol, 4 eq.) in H_2O (25 mL) and THF (25 mL) was stirred at 25 °C for 12 hrs. LCMS showed most of the starting material was consumed. The reaction mixture was adjusted to pH 3 with 1 M aq. HCl solution. The mixture was extracted with DCM (6 x 20 mL). The combined organic layer was dried over Na_2SO_4, filtered and the filtrate was concentrated in vacuum to give the *title* compound (3.0 g, 12.4 mmol, 92% yield) as a white solid. The crude product was used for next step directly. ^1H NMR (400 MHz, $CDCl_3$) δ 8.37 (s, 1H), 2.49 (s, 3H), 1.55 (s, 9H). LRMS (ESI) (m/z) [M + H – *t*-Bu]+ = 187.0

***tert*-butyl (4-((acetimidamidooxy)carbonyl)-2-methyloxazol-5-yl)carbamate**

To a stirring solution of 5-((*tert*-butoxycarbonyl)amino)-2-methyloxazole-4-carboxylic acid (9.3 g, 38.4 mmol, 1 eq.) in THF (90 mL) was added CDI (6.23 g, 38.4 mmol, 1 eq.). The reaction was heated to 60 °C for 1 hour and *N*-hydroxyacetamidine (3.13 g, 42.2 mmol, 1.1 eq.) was added. The reaction was stirred at 15 °C for another 12 hours. LCMS showed the starting material was consumed. The reaction mixture was concentrated under vacuum. DCM (20 mL) and water (20 mL) were added into the mixture and stirred for 0.5 hr. The mixture was filtered and the filter cake was dried in vacuum to give the *title* compound (6.7 g, 22.3 mmol, 58% yield) as a white solid. ^1H NMR (400 MHz, DMSO) δ 9.78 (s, 1H), 2.38 (s, 3H), 1.79 (s, 3H), 1.42 (s, 9H). LRMS (ESI) (m/z) [MH]+ = 299.1

***tert*-butyl (2-methyl-4-(3-methyl-1,2,4-oxadiazol-5-yl)oxazol-5-yl)carbamate (50)**

The reaction mixture of *tert*-butyl (4-((acetimidamidooxy)carbonyl)-2-methyloxazol-5-yl)carbamate (6.7 g, 22.5 mmol, 1 eq.) and K_2CO_3 (12.42 g, 89.8 mmol, 4 eq.) in dioxane (60 mL) was stirred at 110 °C for 48 hours. LCMS showed the starting material was consumed. The reaction mixture was poured into sat. aq. NH_4Cl (150 mL) and extracted with ethyl acetate (3 x 60 mL). The combined organic layer was washed with brine (150 mL), dried over Na_2SO_4, filtered and the filtrate was concentrated. The residue was purified by column chromatography (SiO_2, PE: EA = 10:1 -2:1) to give the *title* compound (3.5 g, 11.9 mmol, 53% yield) as a white solid. ^1H NMR (400 MHz, $CDCl_3$) δ 8.21 (br s, 1H), 2.53 (s, 3H), 2.45 (s, 3H), 1.57 (s, 9H). LRMS (ESI) (m/z) [MH]+ = 281.0

***tert*-butyl (2-methyl-4-(3-methyl-1,2,4-oxadiazol-5-yl)oxazol-5-yl) (pentafluorobenzoyl)carbamate**

To the stirring mixture of *tert*-butyl (2-methyl-4-(3-methyl-1,2,4-oxadiazol-5-yl)oxazol-5-yl)carbamate (4 g, 14.3 mmol, 1 eq.) and TEA (6.0 mL, 43 mmol, 3 eq.) in DCM (40 mL) was added 2,3,4,5,6-pentafluorobenzoyl chloride (2.36 mL, 17.1 mmol, 1.2 eq.) dropwise at 0 °C. Then the solution was stirred at 15 °C for 12 hours. LCMS showed the starting material was consumed. The reaction mixture was poured into sat. aq. NH_4Cl (50 mL) and extracted with DCM (2 x 20 mL). The combined organic layer was washed with brine (50 mL), dried over Na_2SO_4, filtered and the filtrate was concentrated. The residue was purified by column chromatography (SiO_2, PE: EA = 50:1 -5:1) to give the *title* compound(5.5 g, 11.2 mmol, 78% yield) as a white solid. ^1H NMR (400 MHz, $CDCl_3$) δ 2.61 (s, 3H), 2.45 (s, 3H), 1.35 (s, 9H). LRMS (ESI) (m/z) [MH]+ = 474.9.

2,3,4,5,6-pentafluoro-N-(2-methyl-4-(3-methyl-1,2,4-oxadiazol-5-yl)oxazol-5-yl)benzamide (43)

The mixture of *tert*-butyl (2-methyl-4-(3-methyl-1,2,4-oxadiazol-5-yl)oxazol-5-yl) (pentafluorobenzoyl) carbamate (5.5 g, 11.6 mmol, 1 eq.) in TFA (15 mL) and DCM (45 mL) was stirred at 40 °C for 1 hour. TLC (PE: EA=1:1) showed the starting material was consumed. The reaction mixture was concentrated under vacuum. The residue was purified by column chromatography (SiO_2, PE: EA = 10:1 -1:1) to give the *title compound* (4.25 g, 11.3 mmol, 98% yield) as a white solid. ^{1}H NMR (400 MHz, $CDCl_3$) δ 9.66 (s, 1H), 2.60 (s, 3H), 2.45 (s, 3H). HRMS (ESI): m/z calcd for $C_{13}H_8F_5N_4O$ [M+H$^+$]: 375.0511. Found 375.0524

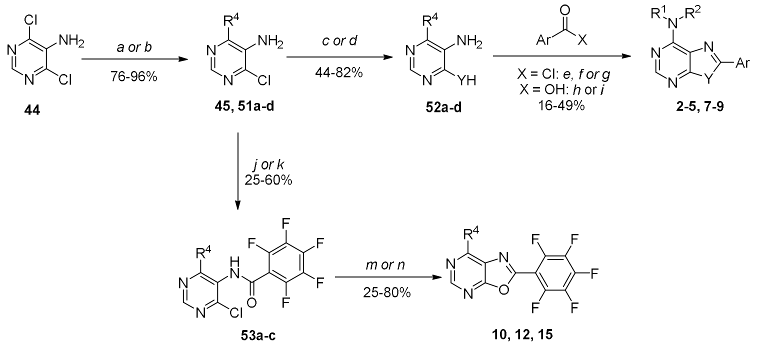

Scheme S1. *Reagents and conditions. a* amine, Et_3N, 2-propanol; *b* NaOEt, ethanol, 75 °C, 6 h; *c* Na_2S, DMF, 80-100 °C; *d* methylamine, ethanol, 140 °C, microwave*; e* acid chloride, CSA, toluene, 100 °C; *f* (i) acid chloride, Et_3N, acetonitrile, rt; (ii) HCl, water, 100 °C; *g* (i) **48d**, pentafluorobenzoyl chloride, acetonitrile, 100 °C, microwave; (ii) camphorsulfonic acid, acetonitrile, 180 °C, microwave; .*h* carboxylic acid, n-propylphosphonic anhydride, triethylamine, ethyl acetate, acetonitrile, 80 °C; *i* carboxylic acid, polyphosphoric acid, 140 °C.; *j* **45** or **47c**, pentafluorobenzoyl chloride, pyridine, dichloromethane; *k* pentafluorobenzoyl chloride, *N,N*-diethylaniline, toluene, 80 °C, 6 h*; m* **46 ,49a or 49b**, polyphosphoric acid, 140 °C; *n* **49c**, Eaton's reagent, 120 °C, 3 h.

4-Chloro-6-morpholinopyrimidin-5-amine (51a)

A solution of 4,6-dichloro-5-pyrimidinamine (1.00 g, 6.10 mmol) in 2-propanol (10 mL) at 0 °C was treated dropwise with morpholine (0.59 mL, 6.7 mmol) and triethylamine (1.02 mL, 7.3 mmol). The mixture was stirred at room temperature for six days. TLC (3:1 EtOAc-heptane) showed incomplete reaction. Further morpholine (0.20 mL, 2.3 mmol) was added and the reaction was stirred for a further 24 hours. TLC (as above) showed complete reaction. The solution was diluted with diethyl ether (50 mL) and the resulting precipitate was removed by filtration, washed with diethyl ether and the combined filtrate was concentrated to give a colourless gum. The crude residue was partitioned between EtOAc (50 mL) and saturated aqueous $NaHCO_3$ (25 mL) and the organic phase was washed with water (25 mL) and brine (25 mL), dried (Na_2SO_4) and concentrated and dried *iv* to give the *title compound* as a colourless gum which solidified slowly on standing to give an off-white solid (1.188 g, 4.98 mmol), 82% yield. ^{1}H NMR (500 MHz, $CDCl_3$) δ 8.20 (s, 1H), 3.89 - 3.85 (m, 6H), 3.38 (t, *J*=4.7 Hz, 4H); LRMS (ESI) (*m/z*) [MH]$^+$ 214.9

6-Chloro-*N*^{4},*N*^{4}-diethylpyrimidine-4,5-diamine (51b)

A solution of 4,6-dichloro-5-pyrimidinamine (1.00 g, 6.10 mmol) in 2-propanol (10 mL) at 0 °C was treated dropwise with diethylamine (0.76 mL, 7.3 mmol) and triethylamine (1.02 mL, 7.3 mmol). The mixture was stirred at room temperature for three days. TLC (5% methanol-dichloromethane) showed no reaction. The mixture was then heated at 50 °C for 24 hours. More diethylamine (0.30 mL, 2.9 mmol) was added and the mixture was heated at 60 °C for 24 hours. TLC (as above) showed mainly unreacted starting material. Further diethylamine (0.30 mL, 2.9 mmol) was added and the temperature was raised to 80 °C. After 7 hours, another portion of diethylamine (0.30 mL, 2.9 mmol) was added and the mixture was heated at 80 °C overnight. TLC (as above) showed complete reaction. The brown solution was cooled, diluted with diethyl ether (50 mL) and the resulting precipitate was removed by filtration, wshed with diethyl ether and the combined filtrate was concentrated to give a brown oil. The crude residue was partitioned between EtOAc (50 mL) and

aqueous pH 5 buffer (20 mL) and the organic phase was washed with water (20 mL), saturated aqueous $NaHCO_3$ (20 mL) and brine (20 mL), dried (Na_2SO_4) and concentrated and dried *iv* to give the *title compound* as a brown oil (1.151 g, 5.45 mmol), 89% yield. ^1H NMR (500 MHz, $CDCl_3$) δ 8.14 (s, 1H), 3.83 (s, 2H), 3.39 (q, *J*=7.0 Hz, 4H), 1.16 (t, *J*=7.0 Hz, 6H); LRMS (ESI) (*m/z*) $[MH]^+$ = 200.9

N^4-Benzyl-6-chloro-N^4-methylpyrimidine-4,5-diamine (51c)

A solution of 4,6-dichloro-5-pyrimidinamine (1.00 g, 6.10 mmol) in 2-propanol (10 mL) at 0 °C was treated dropwise with *N*-benzylmethylamine (0.95 mL, 7.3 mmol) and triethylamine (1.02 mL, 7.3 mmol). The mixture was stirred at room temperature for three days. TLC (5% methanol-dichloromethane) showed no reaction. The mixture was then heated at 50 °C for 24 hours. TLC showed incomplete reaction. The temperature was raised to 60 °C and the was overnight. The pale brown solution was cooled, diluted with diethyl ether (54 mL) and the resulting precipitate was removed by filtration, washed with diethyl ether and the combined filtrate was concentrated to give a brown oil. The crude residue was partitioned between EtOAc (50 mL) and aqueous pH 5 buffer (20 mL), agitated to dissolve the crude, and the organic phase was washed with water (20 mL), saturated aqueous $NaHCO_3$ (20 mL) and brine (20 mL), dried (Na_2SO_4) and concentrated and dried *iv* to give the *title compound* as a brown solid (1.448 g, 5.53 mmol), 91% yield. ^1H NMR (500 MHz, DMSO) δ 7.93 (s, 1H), 7.34 - 7.30 (m, 2H), 7.28 - 7.22 (m, 3H), 4.99 (s, 2H), 4.58 (s, 2H), 2.86 (s, 3H). LRMS (ESI) (*m/z*) $[MH]^+$ =248.9

4-Chloro-6-ethoxy-pyrimidin-5-amine (51d)

To a solution of 4,6-dichloro-5-pyrimidinamine (200 mg, 1.22mmol) in Ethanol (3mL) was added 21% Sodium ethoxide solution in ethanol (455 µL,1.22 mmol) and the reaction was refluxed at 75 °C for 6 hours. The reaction mixture was cooled to room temperature and absorbed onto silica gel. Purification by column chromatography (0-100%, EtOAc-heptane) gave the *title compound* (211 mg, 0.92 mmol) 76 % yield, 80% purity as a semi-solid, which was used without further purification. ^1H NMR (400 MHz, $CDCl_3$) δ 8.04 (s, 1H), 4.51 (q, J=7.1 Hz, 2H), 1.45 (t, J=7.1 Hz, 3H). LRMS (ESI) (*m/z*) $[MH]^+$ =174.1/175.6

5-Amino-6-(dimethylamino)pyrimidine-4-thiol (52a)

To a solution of 6-chloro-N^4,N^4-dimethylpyrimidine-4,5-diamine (5.00 g, 26.6 mmol) in DMF (50 mL) was added sodium monosulfide (3.12 g, 40.0 mmol). The mixture was heated at 80 °C overnight. The brown suspension was poured onto ice (300 mL) to give a dark brown solution. The mixture was acidified to pH 5 with acetic acid to give a brown precipitate which was collected by filtration and washed with water, air dried and then dried *iv* over anhydrous $CaCl_2$ to give the crude *title compound* as a brown solid (2.23 g, 11.8 mmol), 44% yield, which was used without purification. ^1H NMR (500 MHz, DMSO) δ 13.32 (br.s, 1H), 7.77 (d, *J*=3.5 Hz, 1H), 4.86 (br.s, 2H), 2.96 (s, 6H). LRMS (ESI) (*m/z*) $[M-H]^-$ =169.1

5-Amino-6-morpholinopyrimidine-4-thiol (52b)

To a solution of 6-chloro-6-morpholinopyrimidin-5-amine (480 mg, 2.00 mmol) in DMF (8 mL) was added sodium monosulfide (0.24 g, 3.0 mmol). The mixture was heated at 100 °C overnight. The brown suspension was cooled and diluted with water (100 mL) to give a brown solution. The mixture was acidified to pH 5 with acetic acid (no precipitate in this case formed). The aqueous phase was extracted with EtOAc (100 mL, then 2 x 50 mL) and the combined organic extracts were washed with 5% aqueous LiCl (2 x 50 mL) and brine, dried (Na_2SO_4) and concentrated to give the crude *title compound* as a brown solid (403 mg, 1.65 mmol), 82% yield, which was used without purification. ^1H NMR (500 MHz, DMSO) δ 13.58 (br.s, 1H), 7.82 (s, 1H), 4.94 (br.s, 2H), 3.72 (t, *J*=4.6 Hz, 4H), 3.31 (t, *J*=4.6 Hz, 4H); LRMS (ESI) (*m/z*) $[M-H]^-$211.0

5-Amino-6-(diethylamino)pyrimidine-4-thiol (52c)

To a solution of 6-chloro-*N*^{4},*N*^{4}-diethylpyrimidine-4,5-diamine (106 mg, 0.50 mmol) in DMF (2 mL) was added sodium monosulfide (59 mg, 0.76 mmol). The mixture was heated at 100 °C overnight. The brown suspension was cooled and diluted with water (10 mL) to give a brown solution. The mixture was acidified to pH 5 with acetic acid to give a brown precipitate which was collected by filtration and washed with water and then dried *iv* over anhydrous $CaCl_2$ to give the *title compound* as a brown solid (63 mg, 0.30 mmol), 60% yield. ^{1}H NMR (500 MHz, DMSO) δ 13.30 (br.s, 1H), 7.78 (d, *J*=3.5 Hz, 1H), 4.72 (br.s, 2H), 3.42 (q, *J*=7.0 Hz, 4H), 1.08 (t, *J*=7.0 Hz, 6H); LRMS (ESI) (*m/z*) [MH]$^+$ =199.0

***N*^{4},*N*^{4},*N*^{6}-trimethylpyrimidine-4,5,6-triamine (52d)**

A capped process vial containing 6-chloro-N4,N4-dimethyl-pyrimidine-4,5-diamine (111.11mg, 0.5793mmol) and Methylamine, 33% in absolute ethanol (3 mL, 31.86mmol) was heated at 140 °C under microwave irradiation (Biotage Initiator) for 2 hours. The reaction mixture was absorbed onto silica gel and purified by column chromatography (0-100% EtOAc-heptane) to give the *title compound* (80.4mg,0.41mmol, 71% yield, 85% purity), as a waxy brown solid. ^{1}H NMR (500 MHz, DMSO) δ 7.80 (s, 1H), 6.19 - 6.17 (d, J=4.1 Hz, 1H), 4.01 (s, 2H), 2.85 (d, J=4.6 Hz, 3H), 2.78 (s, 1H), 2.67 (s, 6H).

***N*-(4-chloro-6-morpholinopyrimidin-5-yl)-2,3,4,5,6-pentafluorobenzamide (53a)**

To a stirred solution of 4-chloro-6-morpholinopyrimidin-5-amine (239 mg, 1 mmol) in DCM (3 mL) and pyridine (300 µL) was added pentafluorobenzoyl chloride (173 µL, 1.2 mmol) and the reaction was stirred for 22 hours. The reaction mixture was diluted with DCM, washed (sat. NaHCO3, 10% citric acid) and concentrated to give the crude product. Purification by column chromatography (0-50% EtOAc-Heptane) gave the *title compound* as an off-white solid (112 mg, 0.25 mmol) 25% yield. ^{1}H NMR (500 MHz, DMSO) δ 10.85 (s, 1H), 8.42 (s, 1H), 3.68-3.74 (m, 8H); LRMS (ESI) (*m/z*) [MH]$^+$ =409.1

***N*-(4-(Benzyl(methyl)amino)-6-chloropyrimidin-5-yl)-2,3,4,5,6-pentafluorobenzamide (53b)**

To a stirred solution of *N*^{4}-Benzyl-6-chloro-*N*^{4}-methylpyrimidine-4,5-diamine (262 mg, 1 mmol) in DCM (3mL) and Pyridine (300 µL) was added pentafluorobenzoyl chloride (173 µL, 1.2mmol) and the reaction was stirred for 22 hours. The reaction mixture was diluted with DCM, washed (sat. NaHCO3, 10% citric acid) and concentrated to give the crude product. Purification by column chromatography (0-70% EtOAc-Heptane) gave the *title compound* as a pale yellow solid (271 mg, 0.55 mmol) 55% yield. ^{1}H NMR (500 MHz, DMSO) δ 10.82 (s, 1H), 8.34 (s, 1H), 7.36 - 7.32 (m, 2H), 7.29 - 7.25 (m, 3H), 4.99 (bd, J=15.1 Hz, 1H), 4.90 (bd, J=15.0 Hz, 1H), 3.14 (s, 3H). LRMS (ESI) (*m/z*) [M-H]$^-$ = 455.0

***N*-(4-Chloro-6-ethoxy-pyrimidin-5-yl)-2,3,4,5,6-pentafluoro-benzamide (53c)**

To a stirred solution of 4-chloro-6-ethoxy-pyrimidin-5-amine (205mg, 0.89mmol, 80% purity) in Toluene (3mL) was added N,N-diethyl aniline (282 µL, 1.77 mmol) and the reaction was stirred for 10 minutes. 2,3,4,5,6-pentafluorobenzoyl chloride (255 µL, 1.77 mmol) was added slowly and the reaction stirred at 80 °C for 6 hours. The reaction mixture was concentrated to give a crude residue and excess toluene was removed by azeotroping with DCM (2 x 3 mL). Purification by column chromatography (0-10% MeOH-DCM) gave the *title compound* (234 mg, 0.54 mmol), 60% yield, 90% purity, which was used without further purification. ^{1}H NMR (500 MHz, DMSO) δ 10.86 (s, 1H), 8.66 (s, 1H), 4.49 (q, J=7.0 Hz, 2H), 1.35 (t, J=7.1 Hz, 3H). LRMS (ESI) (*m/z*) [MH]$^+$ =367.8/369.8.

General Procedure A for T3P coupling

***N*,*N*-dimethyl-2-(2,3,5,6-tetrafluorophenyl)thiazolo[5,4-*d*]pyrimidin-7-amine (2)**

A suspension of 5-amino-6-(dimethylamino)pyrimidine-4-thiol (51.7 mg, 0.30 mmol) in acetonitrile (2 mL) at room temperature was treated with triethylamine (63.5 µL, 0.46 mmol), 2,3,5,6-tetrafluorobenzoic acid (71 mg, 0.36 mmol) and propylphosphonic anhydride solution, 50 wt% in

EtOAc (0.50 mL, 0.84 mmol) to give a red-brown solution. A precipitate formed slowly. After 0.5 hour, the mixture was heated to 80 °C overnight, then basified to pH >10 by dropwise addition of 2 M aqueous sodium hydroxide (ca. 1.2 mL), then diluted with water (5 mL) and allowed to cool to room temperature. The resulting precipitate was collected by filtration, washed with water, and diethyl ether, air-dried to give the *title compound* as a brown solid (16.9 mg, 0.049 mmol), 16% yield. ^{1}H NMR (500 MHz, DMSO) δ 8.48 - 8.46 (m, 1H), 8.21 - 8.13 (m, 1H), 3.57 (br.s, 6H). LRMS (ESI) (*m/*z) [MH]$^+$ =329.0

_N_,_N_-Dimethyl-2-(3,4,5-trifluorophenyl)thiazolo[5,4-_d_]pyrimidin-7-amine (4)

Following **General Procedure A** above, using 5-amino-6-(dimethylamino)pyrimidine-4-thiol (50 mg, 0.29 mmol), triethylamine (61.4 µL, 0.44 mmol) , 3,4,5-trifluorobenzoic acid (62 mg, 0.35 mmol) and propylphosphonic anhydride solution, 50 wt% in EtOAc (0.52 mL, 0.88 mmol) gave the *title compound* as an off-white solid (46 mg, 0.14 mmol), 48% yield. ^{1}H NMR (500 MHz, DMSO) δ 8.40 (s, 1H), 8.01 (dd, *J*=6.9, 8.2 Hz, 2H), 3.56 (br.s, 6H). LRMS (ESI) (*m/*z) [MH]$^+$ =311.1

_N,N_-Dimethyl-2-phenylthiazolo[5,4-_d_]pyrimidin-7-amine (3)

A suspension of 5-amino-6-(dimethylamino)pyrimidine-4-thiol (50 mg, 0.29 mmol) in toluene (2 mL) at 100 °C was treated with benzoyl chloride (41 µL, 0.35 mmol). The suspended solid formed a large clump. The mixture was heated for 10 minutes to give a light brown suspension and then (1*R*)-(-)-10-camphorsulphonic acid (17 mg, 0.07 mmol) was added and heating was continued at 100 °C for 1 hour. The mixture was cooled and the solid was collected by filtration, washed with toluene and air-dried to give a brown solid. This residue was treated with 2 M aqueous sodium hydroxide (2 mL) and extracted with EtOAc (3 x 2 mL). The combined organic extract was washed with 2 M aqueous sodium hydroxide (2 mL), water (2 mL) and brine (2 mL), dried (Na_2SO_4) and concentrated to give the *title compound* as a brown solid (38.6 mg, 0.14 mmol), 49% yield. ^{1}H NMR (500 MHz, DMSO) δ 8.38 (s, 1H), 8.06 - 8.02 (m, 2H), 7.60 - 7.56 (m, 3H), 3.58 (br.s, 6H). LRMS (ESI) (*m/*z) [MH]$^+$ =257.1

2-(4-Fluorophenyl)-_N_,_N_-dimethyl-thiazolo[5,4-_d_]pyrimidin-7-amine (5)

A suspension of 5-amino-6-(dimethylamino)pyrimidine-4-thiol (50 mg, 0.29 mmol) in acetonitrile (2 mL) at room temperature was treated with 4-fluorobenzoyl chloride (56 mg, 0.35 mmol) and triethylamine (61 µL, 0.44 mmol) to give a red-brown solution which was stirred at room temperature overnight to give a thick brown suspension. The mixture was treated with 1 N aqueous hydrochloric acid (2.0 mL, 2.0 mmol) and heated at 100 °C for one hour to give a brown solution. Further acetonitrile (2 mL) was added after 1 hour and the temperature was lowered to 90 °C. After 3 hours heating, the mixture was diluted with water (~10 mL) and allowed to cool. The thick precipitate was collected by filtration, washed with water, EtOAc and diethyl ether and air-dried to give the product HCl salt as a brown solid (33 mg). The solid was treated with 1 M aqueous NaOH (2 mL) and EtOAc (2 mL) and sonicated until the solid completely dissolved. The phases were separated and the aqueous phase was extracted with further EtOAc (2 x 2 mL). The combined EtOAc extracts were washed with 1 M NaOH (2 mL), water (2 mL) and brine (2 mL), and filtered through a plug of anhydrous sodium sulfate. The filtrate was concentrated to give the *title compound* as a tan coloured solid (22 mg, 0.077 mmol), 26% yield. ^{1}H NMR (500 MHz, d6-acetone) δ 8.35 (s, 1H), 8.15 (dd, *J*=5.3, 8.7 Hz, 2H), 7.35 (t, *J*=8.7 Hz, 2H), 3.63 (br.s, 6H). HRMS (ESI): m/z calcd for $C_{13}H_{12}FN_4S$ [M+H$^+$]: 275.0761. Found 275.0779

_N_,_N_,9-Trimethyl-8-(2,3,4,5,6-pentafluorophenyl)purin-6-amine, (7)

A suspension of N^4,N^4,*N*6-trimethylpyrimidine-4,5,6-triamine (80 mg,0.41 mmol) in MeCN (4 mL) at room temperature was treated with 2,3,4,5,6-pentafluorobenzoyl chloride (71 µL, 0.49 mmol) to give an orange solution and the reaction was heated at 100°C under microwave irradiation (Biotage Initiator) for 10 minutes. TLC (10% MeOH-DCM) showed starting material still present along with formation of new product. Reaction was heated at 100°C under microwave irradiation (Biotage Initiator) for a further 30 minutes. TLC still showed starting material present therefore additional

2,3,4,5,6-pentafluorobenzoyl chloride (71 µL, 0.49 mmol) was added and the reaction heated at 100°C under microwave irradiation (Biotage Initiator) for a further 30 minutes . The reaction mixture was absorbed onto silica gel and purified by column chromatography (0-10% MeOH-DCM), to give an off-white solid. LC-MS *m/z* indicated mono-acylated product therefore the solid was dissolved in MeCN (4mL), (1R)-(-)-10-camphorsulphonic acid (24mg,0.10mmol) added and the reaction heated at 180 °C under microwave irradiation (Biotage Initiator) for 1 hour to give a pale brown solution. LC-MS *m/z* indicated ca. 50% conversion to desired product. LC-MS *m/z* indicated ca. 50% conversion to desired product therefore additional (1R)-(-)-10-camphorsulphonic acid (23.736mg,0.1022mmol) was added and the reaction heated at 180 °C under microwave irradiation (Biotage Initiator) for a further hour. The reaction mixture was diluted with water (10 mL) and basified to pH ~11 by dropwise addition of 2 M aq. NaOH. EtOAc (10 mL) was added, the layers separated and the aqueous layer extracted with EtOAc (3 x 10 mL). The combined organic extracts were washed with water (10 mL) and brine (10 mL), dried (Na_2SO_4) and concentrated to give a brown solid which was was purified by column chromatography (0-5% MeOH-DCM) to give the *title compound* (68 mg, 0.18 mmol), 43%yield, as a white solid. ^{1}H NMR (500 MHz, DMSO) δ 8.35 (s, 1H), 3.66 (s, 3H), 3.50 (br.s, 6H). HRMS (ESI): m/z calcd for $C_{14}H_{11}F_5N_5O$ [M+H$^+$]: 344.0929. Found 344.0904.

_N,N_-Diethyl-2-(pentafluorophenyl)thiazolo[5,4-_d_]pyrimidin-7-amine (8)

A mixture of 5-amino-6-diethylaminopyrimidine-4-thiol (57.5 mg, 0.28 mmol) and pentafluorobenzoic acid (64 mg, 0.30 mmol) was treated with polyphosphoric acid (0.5 mL) and stirred at 140 °C overnight. The reaction mixture was cooled to 95 °C, diluted with water (ca. 2 mL), stirred to give a solution then cooled on ice. The solution was neutralized to pH 7 with 1 M aqueous NaOH. The resulting orange solid was collected by filtration, washed with water and dried *iv*. The aqueous filtrate was extracted with EtOAc (x 3). The combined organic extracts were washed with water (x 2) and brine, dried (Na_2SO_4) and concentrated to give an orange gum. TLC (3:1 EtOAc-heptane) indicated that both the precipitate and the EtOAc extract contained the same desired product. The combined precipitate and extract were dissolved in dichloromethane and purified by column chromatography (0-50% EtOAc-heptane) to give the *title compound* as a colourless solid (32.3 mg, 0.082 mmol), 30% yield. ^{1}H NMR (500 MHz, DMSO) δ 8.46 (s, 1H), 4.19 - 4.13 (m, 2H), 3.87 - 3.80 (m, 2H), 1.26 (t, *J*=6.9 Hz, 6H). HRMS (ESI): m/z calcd for $C_{15}H_{12}F_5N_4S$ [M+H$^+$]: 375.0697. Found 375.0695.

4-(2-(Pentafluorophenyl)thiazolo[5,4-d]pyrimidin-7-yl)morpholine (9)

A mixture of 5-amino-6-morpholino-pyrimidine-4-thiol (60 mg, 0.25 mmol) and pentafluorobenzoic acid (57 mg, 0.27 mmol) was treated with polyphosphoric acid (0.5 mL) and stirred at 140 °C overnight. The reaction mixture was cooled to 95 °C, diluted with water (ca. 2 mL), stirred to give a solution then cooled on ice. The solution was neutralized to pH 7 with 1 M aqueous NaOH. The resulting black-brown solid was collected by filtration, washed with water and dried *iv*. The aqueous filtrate was extracted with EtOAc (x 3). The combined organic extracts were washed with water (x 2) and brine, dried (Na_2SO_4) and concentrated to give a brown gum. TLC (3:1 EtOAc-heptane) indicated that both the precipitate and the EtOAc extract contained the same desired product. The combined precipitate and extract were dissolved in dichloromethane/methanol, absorbed onto silica and purified by column chromatography (0-50% EtOAc-heptane) to give the *title compound* as a colourless solid (9.6 mg, 0.022 mmol), 9% yield. ^{1}H NMR (500 MHz, DMSO) δ 8.52 (s, 1H), 4.34 (m, 4H), 3.78 (t, *J*=4.8 Hz, 4H). HRMS (ESI): m/z calcd for $C_{15}H_{10}F_5N_4OS$ [M+H$^+$]: 389.0490. Found 389.0504.

4-(2-(Perfluorophenyl)thiazolo[5,4-_d_]pyrimidin-7-yl)morpholine (10)

N-(4-chloro-6-morpholinopyrimidin-5-yl)-2,3,4,5,6-pentafluorobenzamide (112 mg, 0.25 mmol) was treated with polyphosphoric acid (1 mL) and stirred at 140 °C for 20 hours. The reaction mixture was cooled to 90 °C, diluted with water (4 mL), stirred to give a solution then cooled on ice. The solution was neutralized to pH 7 with 2 M aqueous NaOH. The resulting precipitate was collected by filtration, washed with water and dried *iv* to give the *title compound* as an off-white solid (41.3 mg, 102 µmol),

41% yield. ^{1}H NMR (500 MHz, DMSO) δ 8.41 (s, 1H), 3.66 (br. s, 4H), 3.29 (br. s, 4H). LRMS (ESI) (*m/z*) $[MH]^+$=373.1

N-Methyl-2-(pentafluorophenyl)oxazolo[5,4-d]pyrimidin-7-amine (12)

N-(4-(benzyl(methyl)amino)-6-chloropyrimidin-5-yl)-2,3,4,5,6-pentafluorobenzamide (122 mg, 0.25 mmol) was treated with polyphosphoric acid (1 mL) and stirred at 140 °C overnight. The reaction mixture was cooled to 95 °C, diluted with water (ca. 2 mL), stirred to give a solution then cooled on ice. The solution was neutralized to pH 7 with 1 M aqueous NaOH. The resulting brown solid was collected by filtration, washed with water and dried *iv* to give the *title compound* as a pale brown solid (70 mg, 0.20 mmol), 80% yield. ^{1}H NMR (500 MHz, DMSO) δ 8.18 (s, 1H), 3.67 (s, 3H). HRMS (ESI): m/z calcd for $C_{12}H_6F_5N_4O$ $[M+H^+]$: 317.0417. Found 317.0471.

2-(2,3,4,5,6-Pentafluorophenyl)oxazolo[5,4-d]pyrimidin-7-ol (15)

N-(4-chloro-6-ethoxy-pyrimidin-5-yl)-2,3,4,5,6-pentafluoro-benzamide (35 mg, 86 µmol) was treated with Eaton's reagent (196 µL, 0.16 mmol) and the reaction was heated at 120°C for 3 hours. The reaction was cooled to room temperature, followed by dropwise addition of water (4 mL). The mixture was neutralised (sat. $NaHCO_3$ solution), extracted with DCM (2 x 10 mL), the organic layer dried ($MgSO_4$) and concentrated. The crude product was purified by prep-HPLC (mobile phase: [water (0.1%FA)-acetonitrile]; B%: 5-95%) to give the *title compound* as a white solid (8 mg, 25 µmol), 29% yield. ^{1}H NMR (500 MHz, DMSO) δ 13.14 (br.s, 1H), 8.37 (d, *J* = 3.1 Hz, 1H). HRMS (ESI): m/z calcd for $C_{11}H_3F_5N_3O_2$ $[M+H^+]$: 304.0140. Found 304.0159.

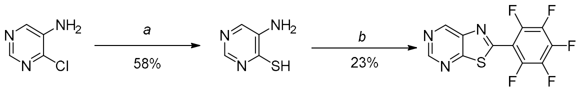

Scheme S2. *Reagents and conditions.a* Na_2S, DMF, 80-100 °C; *b* pentafluorobenzoic acid, polyphosphoric acid, 140 °C

5-Aminopyrimidine-4-thiol

To a solution of 5-amino-4-chloropyrimidine (223 mg, 1.72 mmol) in DMF (5 mL) was added sodium monohydrogensulfide n-hydrate (0.38 g, 5.2 mmol). The mixture was stirred at room temperature overnight. The green suspension was poured into water (50 mL) to give a brown solution. The mixture was acidified to pH 5 with acetic acid to give a very fine precipitate. The mixture was extracted with EtOAc (3 x 25 mL) and the combined organic extracts were washed with water (3 x 10 mL) and brine (10 mL), dried (Na_2SO_4) and concentrated to give the crude *title compound* as a brown solid (141 mg, 1.00 mmol), 58% yield, *ca.* 90% pure, which was used without purification. ^{1}H NMR (500 MHz, DMSO) δ 13.98 (br.s, 1H), 7.79 (s, 1H), 7.51 (s, 1H), 5.71 (br.s, 2H). LRMS (ESI) (*m/z*) $[MH]^+$ = 128.0

2-(Pentafluorophenyl)thiazolo[5,4-d]pyrimidine (11)

A mixture of 5-aminopyrimidine-4-thiol (51.8mg, 0.41 mmol) and pentafluorobenzoic acid (95 mg, 0.45 mmol) was treated with polyphosphoric acid (0.5 mL) and stirred at 140 °C overnight. The mixture was allowed to cool to room temperature to give a brown gum. This was dissolved in 1 M aqueous NaOH with sonication. The resulting brown solution was neutralised to pH 7 by addition of further 1 M NaOH and extracted with EtOAc (2 x 15 mL). The combined organic extracts were washed with water (x 3) and brine, dried (Na_2SO_4) and concentrated to give a brown solid, which was purified by column chromatography (0-50% EtOAc-heptane) to give the *title compound* as a pale brown solid (29.8 mg, 0.093 mmol), 23% yield. ^{1}H NMR (500 MHz, DMSO) δ 9.72 (s, 1H), 9.32 (s, 1H). HRMS (ESI): m/z calcd for $C_{11}H_3F_5N_3S$ $[M+H^+]$: 303.9962. Found 303.9983.

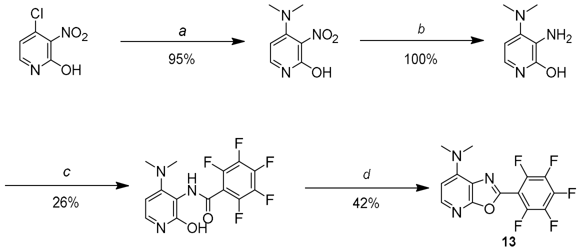

Scheme S3. *Reagents and conditions.* a $Me_2NH.HCl$, triethylamine, ethanol; *b* Pd/C, H_2, MeOH, 15 psi; *c* pentafluorobenzoyl chloride, triethylamine, DCM; *d* P_2O_5, 175°C

4-(dimethylamino)-3-nitropyridin-2-ol

The reaction mixture of 4-chloro-3-nitropyridin-2-ol (500 mg, 2.86 mmol, 1 eq.), dimethylamine hydrochloride (700 mg, 8.59 mmol, 3 eq.) and TEA (1.59 mL, 11.5 mmol, 4 eq.) in ethanol (5 mL) was stirred at 25 °C for 12 hours. LC-MS showed starting material was consumed and the desired mass was observed. The reaction mixture was concentrated under vacuum to give the *title compound* (500 mg, 2.73 mmol, 95% yield) as a light yellow solid. LRMS (ESI) (*m/*z) $[MH]^+$=184.0

3-amino-4-(dimethylamino)pyridin-2-ol

To compound 4-(dimethylamino)-3-nitropyridin-2-ol (600 mg, 3.28 mmol, 1 *eq*) in MeOH (6 mL) was added 10% Pd/C (0.1 g) under N_2. The suspension was degassed under vacuum and purged with H_2 three times. The reaction mixture was stirred under H_2 (15 psi) at 25 °C for 12 hours. LC-MS showed starting material was consumed. The reaction mixture was filtered and the filtrate was concentrated to give the *title compound* (500 mg, 3.26 mmol, 100% yield) as a grey solid. LRMS (ESI) (*m/*z) $[MH]^+$ =154.1

N-(4-(dimethylamino)-2-hydroxypyridin-3-yl)-2,3,4,5,6-pentafluorobenzamide

To the reaction mixture of compound 3-amino-4-(dimethylamino)pyridin-2-ol (500 mg, 3.26 mmol, 1 eq) and TEA (660 mg, 6.53 mmol, 2 eq) in DCM (5 mL) was added 2,3,4,5,6-pentafluorobenzoyl chloride (677 mg, 2.94 mmol, 0.9 eq) at 0 °C. Then the reaction was stirred at 25 °C for 12 hours. LC-MS showed most of the starting material was consumed and the desired mass was observed. The reaction mixture was concentrated under vacuum. The crude product was purified by reversed-phase flash chromatography (mobile phase A: 0.1% formic acid in water; mobile phase B: acetonitrile; gradient 0-100%) to give the *title compound* (300 mg, 863 µmol, 26.47% yield, 100% purity) as a white solid. ^1H NMR (400 MHz, DMSO) δ 10.93 (br s, 1H), 9.68 (s, 1H), 7.11 (br d, *J* = 7.6 Hz, 1H), 5.95 (d, *J* = 7.6 Hz, 1H), 2.94 (s, 6H). LRMS (ESI) (*m/*z) $[MH]^+$=348.2.

N,N-dimethyl-2-(perfluorophenyl)oxazolo[5,4-*b*]pyridin-7-amine (13)

The reaction mixture of *N*-(4-(dimethylamino)-2-hydroxypyridin-3-yl)-2,3,4,5,6-pentafluorobenzamide (100 mg, 288 µmol, 1 eq) and P_2O_5 (82 mg, 0.58 mmol, 35.55 µL, 2 eq.) was stirred at 175 °C for 1 hour. LC-MS showed most of the starting material was consumed and the desired mass was observed. The reaction mixture was poured into ice-water (5 mL) and extracted with EtOAc (2 mL x 3). The combined organic layer was washed with brine (5 mL), dried over Na_2SO_4, filtered and the filtrate was concentrated. The crude product was purified by Prep-HPLC (column: Phenomenex Synergi C18 150x25x10 µm; mobile phase: [water (0.225%FA)-acetonitrile]; B%: 48%-78%,10 min) to obtain the *title compound* (42.2 mg, 121 µmol, 42% yield) as a white solid. ^1H NMR (400 MHz, DMSO) δ 7.99 (d, *J* = 6.0 Hz, 1H), 6.61 (d, *J* = 6.0 Hz, 1H), 3.37 (s, 6H). LRMS (ESI) (*m/*z) $[MH]^+$=330.0

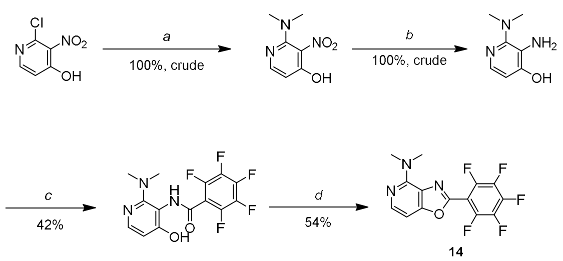

Scheme S4. *Reagents and conditions.* a $Me_2NH.HCl$, triethylamine, ethanol; *b* Pd/C, H_2, MeOH, 15 psi; *c* pentafluorobenzoyl chloride, triethylamine, DCM; *d* P_2O_5, 175°C

2-(dimethylamino)-3-nitropyridin-4-ol

The reaction mixture of 2-chloro-3-nitropyridin-4-ol (0.5 g, 2.86 mmol, 1 eq.), dimethylamine hydrochloride (699 mg, 8.58 mmol, 3 eq.) and TEA (1.59 mL, 11.4 mmol, 4 eq.) in EtOH (5 mL) was stirred at 25 °C, then heated to 90 °C for 12 hours. LC-MS showed starting material was consumed and the desired mass was observed. The mixture was concentrated under vacuum to give the *title compound* (500 mg, crude) as a yellow solid. The crude product was used for the next step without purification. LRMS (ESI) (*m/*z) $[MH]^+$=184.0

3-amino-2-(dimethylamino)pyridin-4-ol

To a solution of 2-(dimethylamino)-3-nitropyridin-4-ol (500 mg, 2.73 mmol, 1 *eq.*) in MeOH (20 mL) was added Pd/C (50 mg, 10% purity) under N_2. The suspension was degassed under vacuum and purged with H_2 several times. The mixture was stirred under H_2 (15 psi) at 25 °C for 12 hours. LC-MS showed the starting material was consumed and the desired mass was observed. The reaction mixture was filtered and the filtrate was concentrated to give the *title compound* (400 mg, crude) as a yellow solid. The crude product was used for the next step directly without purification. LRMS (ESI) (*m/*z) $[MH]^+$=154.0

N-(2-(dimethylamino)-4-hydroxypyridin-3-yl)-2,3,4,5,6-pentafluorobenzamide

To a solution of 3-amino-2-(dimethylamino)pyridin-4-ol (400 mg, 2.61 mmol, 1 *eq.*) and TEA (1.09 mL, 7.83 mmol, 3 *eq.*) in DCM (4 mL) was added 2,3,4,5,6-pentafluorobenzoyl chloride (602 mg, 2.61 mmol, 1 *eq.*) at 0 °C. The mixture was stirred at 25 °C for 12 hours. LC-MS showed the stating material was consumed and 54% of the desired mass was found. The reaction mixture was concentrated under reduced pressure. The crude product was purified by reverse-phase HPLC (mobile phase A: 0.1% formic acid in water; mobile phase B: acetonitrile; gradient 0-100%) to give the *title compound* (400 mg, 1.10 mmol, 42% yield) as a pink solid.^{1}H NMR (400 MHz, MeOD) δ 8.11 (s, 1H), 7.50 (d, *J* = 7.2 Hz, 1H), 6.31 (d, *J* = 7.2 Hz, 1H), 3.05 (s, 6H). LRMS (ESI) (*m/*z) $[MH]^+$=348.1

N,N-dimethyl-2-(perfluorophenyl)oxazolo[4,5-*c*]pyridin-4-amine (14)

The reaction mixture of *N*-(2-(dimethylamino)-4-hydroxypyridin-3-yl)-2,3,4,5,6-pentafluorobenzamide (100 mg, 0.29 mmol, 1 *eq.*) and P_2O_5 (82 mg, 0.58 mmol, 2 *eq.*) was stirred at 25 °C. The reaction was then stirred at 175 °C for 4 hours. LC-MS showed compound **4** was consumed and the desired mass was found. The reaction mixture was poured into ice-H_2O (5 mL) and extracted with EtOAc (2 mL x 3). The combined organic layer was washed with brine (5 mL), dried over Na_2SO_4, filtered and the filtrate was concentrated. The residue was diluted with MeOH and purified by prep-HPLC (column: Shim-pack C18 150x25x10 µm; mobile phase: [water (0.225% FA)-acetonitrile]; B%: 19%-55%,12 min) to obtained the *title compound* (55.9 mg, 154 µmol, 54% yield) as a yellow solid. ^{1}H NMR (400 MHz, DMSO) δ 8.11 (d, J = 5.6 Hz, 1H), 7.10 (d, J = 5.6 Hz, 1H), 3.41 (s, 6H). LRMS (ESI) (*m/*z) $[MH]^+$=330.0

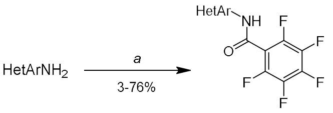

16-22, 34

Scheme S5. *Reagents and conditions. a* pentafluorobenzoyl chloride, 4-methylmorpholine, acetonitrile, 60 °C

Methyl 4-(pentafluorobenzamido)nicotinate (16) (General Procedure B)

A solution of methyl 4-aminonicotinate (38 mg, 0.25 mmol) in acetonitrile (1 mL) was treated with 4-methylmorpholine (55 µL, 0.50 mmol) and pentafluorobenzoyl chloride (40 µL, 0.28 mmol) and heated at 60 °C overnight. The reaction mixture was cooled and diluted with MeCN/water (0.5 mL). The solution was purified by mass-directed HPLC (20-95% acetonitrile-water + 0.1% formic acid) to give the *title compound* (32.6 mg, 91 µmol), 37% yield. ^{1}H NMR (500 MHz, DMSO) δ 11.47 (s, 1H), 9.03 (s, 1H), 8.77 (d, *J*=5.6 Hz, 1H), 8.19 (d, *J*=5.6 Hz, 1H), 3.89 (s, 3H). HRMS (ESI): m/z calcd for $C_{14}H_8F_5N_2O_3$ [M+H$^+$]: 347.0450. Found 347.0481.

Methyl 2-(perfluorobenzamido)nicotinate (17)

Following **General Procedure B**, methyl 2-aminonicotinate (38 mg, 0.25 mmol) gave the *title compound* (19.5 mg, 51 µmol), 20% yield. ^{1}H NMR (500 MHz, DMSO) δ 11.63 (s, 1H), 8.61 (dd, *J*=4.8 and 1.8 Hz, 1H), 8.20 (d, *J*=7.7 and 1.8 Hz, 1H), 7.45 (d, *J*=7.7 and 4.8 Hz, 1H), 3.79 (s, 3H). HRMS (ESI): m/z calcd for $C_{14}H_8F_5N_2O_3$ [M+H$^+$]: 347.0450. Found 347.0479.

Methyl 3-(pentafluorobenzamido)isonicotinate (18)

Following **General Procedure B**, methyl 3-aminoisonicotinate (38 mg, 0.25 mmol) gave the *title compound* (5.6 mg, 15 µmol), 6% yield. ^{1}H NMR (500 MHz, DMSO) δ 11.24 (s, 1H), 8.90 (s, 1H), 8.63 (d, *J*=5.0 Hz, 1H), 7.75 (d, *J*=5.0 Hz, 1H), 3.84 (s, 3H). HRMS (ESI): m/z calcd for $C_{14}H_8F_5N_2O_3$ [M+H$^+$]: 347.0450. Found 347.0475

Methyl 1-methyl-4-(pentafluorobenzamido)-1*H*-pyrazole-5-carboxylate (19)

Following **General Procedure B**, methyl 4-amino-1-methyl-1H-pyrazole-3-carboxylate (39 mg, 0.25 mmol) gave the *title compound* (28 mg, 76 µmol), 30% yield. ^{1}H NMR (500 MHz, DMSO) δ 10.28 (s, 1H), 8.42 (s, 1H), 3.95 (s, 3H), 3.83 (s, 3H). HRMS (ESI): m/z calcd for $C_{13}H_9F_5N_3O_3$ [M+H$^+$]: 350.0559. Found 350.0583.

Methyl 1,3-dimethyl-4-(pentafluorobenzamido)-1*H*-pyrazole-5-carboxylate (20)

Following **General Procedure B**, methyl 4-amino-1-methyl-1H-pyrazole-3-carboxylate (42 mg, 0.25 mmol) gave the *title compound* (74.5 mg, 191 µmol), 76% yield. ^{1}H NMR (500 MHz, DMSO) δ 10.34 (s, 1H), 3.85 (s, 3H), 3.77 (s, 3H), 2.19 (s, 3H). HRMS (ESI): m/z calcd for $C_{14}H_{11}F_5N_3O_3$ [M+H$^+$]: 364.0715. Found 364.0722.

Ethyl 1-methyl-4-(pentafluorobenzamido)-1*H*-imidazole-5-carboxylate (21)

A solution of ethyl 4-amino-1-methyl-1H-imidazole-5-carboxylate (42 mg, 0.25 mmol) in acetonitrile (1 mL) was treated with 4-methylmorpholine (55 µL, 0.50 mmol) and pentafluorobenzoyl chloride (40 µL, 0.28 mmol) and heated at 60 °C overnight. The reaction mixture was cooled and diluted with MeCN/water (0.5 mL). A suspension formed. The mixture was diluted further with water and the precipitate was collected by filtration, washed with water and diethyl ether and dried *iv* over CaCl2 to give the *title compound* (2.6 mg, 6.8 µmol), 3% yield. ^{1}H NMR (500 MHz, DMSO) δ 10.81 (s, 1H), 7.85 (s, 1H), 4.23 (q, *J*=6.5 Hz, 2H), 3.83 (s, 3H), 1.24 (t, *J*=6.5 Hz, 3H). HRMS (ESI): m/z calcd for $C_{14}H_{11}F_5N_3O_3$ [M+H$^+$]: 364.0715. Found 364.0729.

Methyl 3-methyl-5-(pentafluorobenzamido)isoxazole-4-carboxylate (22)

Following **General Procedure B**, methyl 5-amino-3-methyl-1,2-oxazole-4-carboxylate (39 mg, 0.25 mmol) gave impure product after HPLC purification. This was re-purified by column chromatography (0-60% EtOAc-heptane) to give the *title compound* as a colourless solid (27 mg, 73 µmol), 29% yield. ^{1}H NMR (500 MHz, DMSO) δ 12.11 (s, 1H), 3.78 (s, 3H), 2.39 (s, 3H). HRMS (ESI): m/z calcd for $C_{13}H_8F_5N_2O_4$ [M+H$^+$]: 351.0399. Found 351.0392.

Ethyl 5-(pentafluorobenzamido)-2-phenyloxazole-4-carboxylate (34)

Following **General Procedure B**, ethyl 5-amino-2-phenyloxazole-4-carboxylate (58 mg, 0.25 mmol) gave the *title compound* as a white solid (28 mg, 64 µmol), 26% yield. ^{1}H NMR (500 MHz, DMSO) δ 11.96 (s, 1H), 8.01 - 7.97 (m, 2H), 7.62 - 7.58 (m, 3H), 4.31 (q, *J*=7.1 Hz, 2H), 1.29 (t, *J*=7.1 Hz, 3H). HRMS (ESI): m/z calcd for $C_{19}H_{12}F_5N_2O_4$ [M+H$^+$]: 427.0712. Found 427.0712.

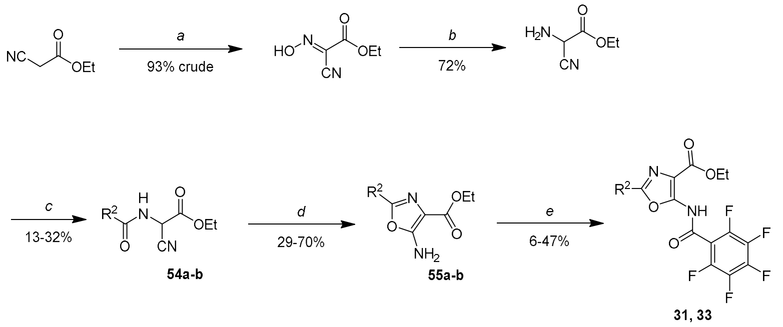

Scheme S6. *Reagents and conditions. a* $NaNO_2$, AcOH, water, 0-20 °C; *b* PtO_2, H_2, EtOH, 60 °C; *c* acid chloride, Et_3N, dichloromethane; *d* HCl/dioxane then aq. K_2CO_3; *e* pentafluorobenzoyl chloride, Et_3N, dichloromethane

Ethyl 2-cyano-2-(hydroxyimino)acetate

To a suspension of ethyl 2-cyanoacetate (47.2 mL, 0.44 mol, 1 *eq.*) and $NaNO_2$ (36.6 g, 0.53 mol, 1.2 *eq.*) in water (450 mL) was added acetic acid (33.4 mL, 0.58 mol, 1.3 *eq.*) at 0-5 °C over a period of 1 hour. The temperature was slowly raised to 20 °C and the reaction mixture was stirred for 1 hour at 20 °C. TLC (PE: EA=1:1, Rf= 0.65) showed a new spot was formed. The mixture was extracted with ethyl acetate (6 x 150 mL), The combined organic layers were washed with sat. aq. $NaHCO_3$ (3 x 100 mL), brine(3 x 100mL), dried and concentrated under vacuum to give the *title compound* (58 g, 0.41 mol, 93% crude yield) as off-white solid, which was used in the next step without purification. ^{1}H NMR (400 MHz, DMSO) δ 4.31 (q, *J* = 7.1 Hz, 2H), 1.28 (t, *J* = 7.1 Hz, 3H).

Ethyl 2-amino-2-cyanoacetate

To ethyl 2-cyano-2-(hydroxyimino)acetate (10 g, 70.4 mmol, 1 eq.) in ethanol (100 mL) was added PtO_2 (799 mg, 3.52 mmol, 0.05 eq.) under nitrogen. The suspension was degassed under vacuum and purged with hydrogen gas three times. The reaction mixture stirred under hydrogen (15 psi) at 60 °C for 12 hours. LC-MS showed the compound **S16** was. The reaction mixture was filtered, and the filtrate was concentrated to obtain the *title compound* (6.5 g, 50.7 mmol, 72% yield) as a brown oil, which was used in the next step without purification. ^{1}H NMR (400 MHz, $CDCl_3$) δ 4.79 (s, 1H), 4.20 (q, *J* = 7 Hz, 2H), 1.24 (t, *J*=7 Hz, 3H).

Ethyl 2-cyano-2-isobutyramidoacetate (54a)

To the reaction mixture of ethyl 2-amino-2-cyanoacetate (500 mg, 3.90 mmol, 1 eq.) and TEA (1.63 mL, 11.7 mmol, 3 eq.) in DCM (5 mL) was added 2-methylpropanoyl chloride (0.49 mL, 4.7 mmol, 1.2 eq) at 0 °C. Then the solution was stirred at 25 °C for 1 hour. TLC (PE: EA=1:1) showed the starting material was consumed. The reaction mixture was poured into water (5 mL) and extracted with DCM (3 x 5 mL). The combined organic layer was washed with brine (15 mL), dried over Na_2SO_4, filtered and

the filtrate was concentrated. The residue was purified by column chromatography (SiO_2, PE: EA = 50:1-1:1) to the *title compound* (250 mg, 1.26 mmol, 32% yield) as a light yellow solid. ^{1}H NMR (400 MHz, $CDCl_3$) δ 6.27 (m, 1H), 5.54 (d, *J*=8 Hz, 1H), 4.37 (q, *J*=7.2 Hz, 2H), 2.51 (m, 1H), 1.38 (t, J = 7.2 Hz, 3H), 1.22 (m, 6H).

Ethyl 2-cyano-2-(2-methoxyacetamido)acetate (54b)

To a stirring mixture of ethyl 2-amino-2-cyanoacetate (500 mg, 3.90 mmol, 1 *eq.*) and TEA (1.63 mL, 11.7 mmol, 3 *eq.*) in DCM (5 mL) was added 2-methoxyacetyl chloride (0.43 mL, 4.68 mmol, 1.2 *eq.*) at 0 °C, then the mixture was warmed to 25 °C for 12 hours. TLC (PE: EA=1:1) showed the starting material was consumed. The reaction mixture was poured into water (7 mL) and extracted with ethyl acetate (3 x 7 mL). The organic layer was washed with brine (10 mL), dried over Na_2SO_4, filtered and the filtrate was concentrated. The residue was purified by flash silica gel chromatography (0-20% EA/PE**)** to obtain the *title compound* (100 mg, 0.50 mmol, 13% yield) as a yellow oil. ^{1}H NMR (400 MHz, $CDCl_3$) δ 7.38 - 7.29 (m, 1H), 5.60 - 5.54 (m, 1H), 4.42 - 4.35 (m, 2H), 4.03 - 4.00 (m, 2H), 3.48 (s, 3H), 1.41 - 1.36 (m, 3H).

Ethyl 5-amino-2-isopropyloxazole-4-carboxylate (55a)

The reaction mixture of ethyl 2-cyano-2-isobutyramidoacetate (250 mg, 1.26 mmol, 1 eq.) in HCl/dioxane (4 M, 4 mL, 12.6 eq.) was stirred at 25 °C for 3 hours. LC-MS showed starting material was consumed. The reaction mixture was poured into aq. K_2CO_3 (1M, 5 mL) and extracted with ethyl acetate (3 x 2 mL). The combined organic layer was washed with brine (5 mL), dried over Na_2SO_4, filtered and the filtrate was concentrated. The residue was purified by column chromatography (SiO_2, PE: EA = 10:1 -1:1) to obtain the *title compound* (220 mg, 0.89 mmol, 70% yield, 80% purity) as an off-white solid. ^{1}H NMR (400 MHz, $CDCl_3$) δ 5.34 (br. s, 2H), 4.35 (q, J = 7.1 Hz, 2H), 2.98 (m, 1H), 1.38 (t, J = 7.1 Hz, 3H), 1.31 (d, J = 7.0 Hz, 6H). LRMS (ESI) (*m/*z) $[MH]^+$ =199.0

Ethyl 5-amino-2-(methoxymethyl)oxazole-4-carboxylate (55b)

A mixture of ethyl 2-cyano-2-(2-methoxyacetamido)acetate (140 mg, 0.70 mmol, 1 *eq.*) in HCl/dioxane (4 M, 1.4 mL, 8 *eq.*) was stirred at 25 °C for 2 hours. TLC (PE: EA=1:1) showed the starting material was consumed. The reaction mixture was poured into aq. K_2CO_3 (5 mL) and extracted with EA (3 x 2 mL). The combined organic layer was washed with brine (5 mL), dried over Na_2SO_4, filtered and the filtrate was concentrated. The crude product was purified by prep-TLC (PE: EA=1:1) to obtain the *title compound* (40 mg, 0.20 mmol, 29% yield) as a yellow solid. LRMS (ESI) (*m/*z) $[MH]^+$ =201

Ethyl 2-isopropyl-5-(pentafluorobenzamido)oxazole-4-carboxylate (31)

To the reaction mixture of ethyl 5-amino-2-isopropyloxazole-4-carboxylate (100 mg, 0.40 mmol, 1 eq.) and TEA (169 µL, 1.21 mmol, 3 eq) in DCM (1 mL) was added 2,3,4,5,6-pentafluorobenzoyl chloride (67 µL, 0.48 mmol, 1.2 eq) at 0 °C. Then the solution was stirred at 25 °C for 12 hours. LC-MS showed most of the starting material was consumed and the desired mass was present. The reaction mixture was concentrated under vacuum. The crude product was purified by Prep-HPLC (column: Phenomenex Synergi C18 150mmx25mmx10µm; mobile phase: [water (0.225% formic acid)-MeCN];B%: 48%-78%, 8.5min) to obtain the *title compound* (74.3 mg, 0.19 mmol, 47% yield) as an off-white solid. ^{1}H NMR (400 MHz, MeOD) δ 4.34 (q, *J*=7.2 Hz, 2H), 3.18 - 3.07 (m, 1H), 1.39 - 1.31 (m, 9H). LC-MS *m/z* 393.0 $[M+H]^+$; HRMS (ESI): m/z calcd for $C_{16}H_{14}F_5N_2O_4$ $[M+H^+]$: 393.0868. Found 393.0867.

Ethyl 2-(methoxymethyl)-5-(pentafluorobenzamido)oxazole-4-carboxylate (33)

To a mixture of ethyl 5-amino-2-(methoxymethyl)oxazole-4-carboxylate (40 mg, 0.20 mmol, 1 *eq.*) and TEA (83 µL, 0.60 mmol, 3 *eq.*) in DCM (0.5 mL) was added 2,3,4,5,6-pentafluorobenzoyl chloride (27.6 µL, 0.20 mmol, 1 *eq.*) at 0 °C, then warmed to 25 °C for 12 hours. LC-MS showed the starting material was consumed. The reaction mixture was concentrated under vacuum. The residue was purified by Prep-HPLC (column: Unisil 3-100 C18 Ultra 150 mm x 50 mm x 3 µm; mobile phase: [water(0.225%

formic acid)-MeCN]; B%: 43%-63%, 10 min) to obtain the *title compound* (32 mg, 13 µmol, 6% yield) as a brown solid. ^{1}H NMR (400 MHz, $CDCl_3$) δ 4.57 (s, 2H), 4.43 (q, *J* = 7.1 Hz, 2H), 3.48 (s, 3H), 1.42 (t, *J* = 7.2 Hz, 3H). HRMS (ESI): m/z calcd for $C_{15}H_{12}F_5N_2O_5$ [M+H$^+$]: 395.0661. Found 395.0668.

Ethyl 2-(dimethylamino)oxazole-4-carboxylate

A mixture of ethyl 2-chlorooxazole-4-carboxylate (1 g, 5.70 mmol, 1 *eq.*) in dimethylamine solution (2 M in THF, 15 mL, 5.3 *eq.*) was stirred for 2 hours at 70 °C. LC-MS showed the starting material was consumed. The residue was poured into ice-water (1:1, 10 mL) and stirred for 5 minutes. The aqueous phase was extracted with ethyl acetate (3 x 50 mL). The combined organic phase was washed with brine (20 mL), dried with anhydrous Na_2SO_4, filtered and concentrated in vacuum. The crude product was purified by silica gel chromatography (SiO_2, PE: EA=20:1 to 5:1) to obtain the *title compound* (820 mg, 4.45 mmol, 78% yield) as a yellow solid. ^{1}H NMR (400 MHz, $CDCl_3$) δ 7.75 (s, 1H), 4.34 (q, J=7.1 Hz, 2H), 3.08 (s, 6H), 1.35 (t, J=7.1 Hz, 3H). LRMS (ESI) (*m/z*) [MH]$^+$ =185.2

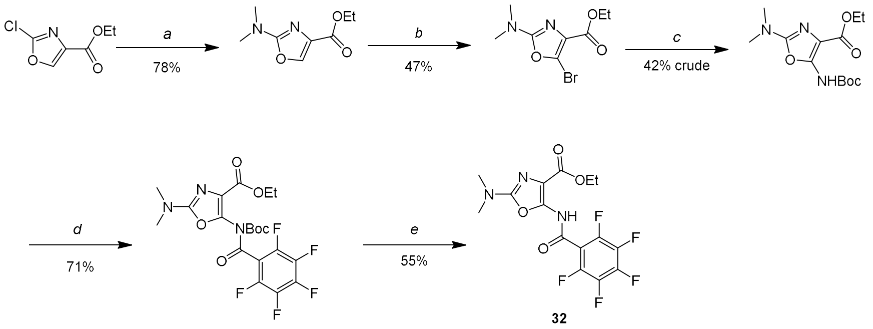

Scheme S7. *Reagents and conditions. g* dimethylamine, THF, 70 °C, 2 h ; *h N*-bromosuccinimide, chloroform, 70 °C, 10 h *i tert*-butylcarbamate, $Pd_2(dba)_3$, BINAP, Cs_2CO_3, toluene, 90 °C, 10 h; *j* pentafluorobenzoyl chloride, Et_3N, dichloromethane; *k* TFA, dichloromethane

Ethyl 5-bromo-2-(dimethylamino)oxazole-4-carboxylate

A mixture of ethyl 2-(dimethylamino)oxazole-4-carboxylate (600 mg, 3.26 mmol, 1 *eq.*) and NBS (696 mg, 3.91 mmol, 1.2 *eq.*) in chloroform (5 mL) was stirred for 10 hours at 70 °C. LC-MS showed the starting material was consumed. The residue was poured into ice-water (1:1, 10 mL) and stirred for 5 min. The aqueous phase was extracted with ethyl acetate (3 x 50 mL). The combined organic phase was washed with brine (20 mL), dried with anhydrous Na_2SO_4, filtered and concentrated in vacuum. The crude product was purified by silica gel chromatography (SiO_2, PE: EA =20:1 to 3:1) to give the *title compound* (400 mg, 1.52 mmol, 47% yield) as a yellow solid. ^{1}H NMR (400 MHz, $CDCl_3$) δ 4.38 (q, *J*=7.1 Hz, 2H), 3.08 (s, 6H), 1.39 (t, *J*=7.1 Hz, 3H). LRMS (ESI) (*m/z*) [MH]$^+$ =263.0/265.0

Ethyl 5-((*tert*-butoxycarbonyl)amino)-2-(dimethylamino)oxazole-4-carboxylate

A mixture of ethyl 5-bromo-2-(dimethylamino)oxazole-4-carboxylate(200 mg, 0.76 mmol, 1 *eq.*), *tert*-butylcarbamate (107 mg, 0.91 mmol, 1.2 *eq.*), $Pd_2(dba)_3$ (139 mg, 0.15 mmol, 0.2 *eq.*), Cs_2CO_3 (495 mg, 1.52 mmol, 2 *eq.*) and BINAP (237 mg, 0.38 mmol, 0.5 *eq.*) in toluene (10 mL) was stirred for 10 hours at 90 °C under N_2. LC-MS showed the starting material was consumed. The residue was poured into ice-water (1:1, 10 mL) and stirred for 5 minutes. The aqueous phase was extracted with ethyl acetate (3 x 50 mL). The combined organic phase was washed with brine (20 mL), dried with anhydrous Na_2SO_4, filtered and concentrated in vacuum. The crude product was added into acetonitrile (50 mL), and the mixture was stirred for 5 minutes, then filtered and the filtrate was concentrated in vacuum to give the *title compound* (95 mg, crude) as a yellow solid, which was used in the next step without purification. ^{1}H NMR (400 MHz, $CDCl_3$) δ 7.88 (br s, 1H), 4.29 (q, *J*=7.1 Hz, 2H), 2.99 (s, 6H), 1.44 (s, 9H), 1.31 (t, *J*=7.2 Hz, 3H). LRMS (ESI) (*m/z*) [MH]$^+$ =300.1

Ethyl 5-(*N*-(*tert*-butoxycarbonyl)-2,3,4,5,6-pentafluorobenzamido)-2-(dimethylamino)oxazole-4-carboxylate

To a mixture of ethyl 5-((*tert*-butoxycarbonyl)amino)-2-(dimethylamino)oxazole-4-carboxylate (70 mg, 0.23 mmol, 1 *eq.*) and TEA (61.3 µL, 0.44 mmol, 1.9 *eq.*) in DCM (1 mL) was added 2,3,4,5,6-pentafluorobenzoyl chloride (32.3 µL, 0.23 mmol, 1 *eq.*) at 20 °C, then the mixture was stirred for 30 minutes at 20 °C. LC-MS showed the starting material was consumed. The mixture was concentrated under vacuum. The residue was purified by prep-TLC (SiO_2, PE: EA=1:1) to give the *title compound* (82 mg, 0.17 mmol, 71% yield) as a yellow solid. LRMS (ESI) (*m/z*) $[MH]^+$ =494.0

Ethyl 2-(dimethylamino)-5-(pentafluorobenzamido)oxazole-4-carboxylate (32)

To a mixture of ethyl 5-(*N*-(*tert*-butoxycarbonyl)-2,3,4,5,6-pentafluorobenzamido)-2-(dimethylamino)oxazole-4-carboxylate (60 mg, 0.12 mmol, 1 *eq.*) in DCM (1 mL) was added TFA (0.5 mL, 6.8 mmol, 55 *eq.*) at 20 °C, then the mixture was stirred for 15 minutes at 20 °C. LC-MS showed the starting material was consumed. The reaction mixture was concentrated in vacuum and the residue was purified by prep-HPLC (column: Phenomenex Synergi C18 150x25x10µm; mobile phase: [water (0.225% formic acid)-MeCN]; B%: 40%-70%, 8.5 min) and prep-TLC (SiO_2 PE:EA=1:1) to give the *title compound* (26.5 mg, 66.7 umol, 55% yield) as a white solid. ^{1}H NMR (400 MHz, MeOD) δ 4.22 (q, *J* = 7.1 Hz, 2H), 3.05 - 2.90 (m, 6H), 1.22 (t, *J* = 7.1 Hz, 3H). HRMS (ESI): m/z calcd for $C_{15}H_{13}F_5N_3O_4$ $[M+H^+]$: 394.0821. Found 394.0845.

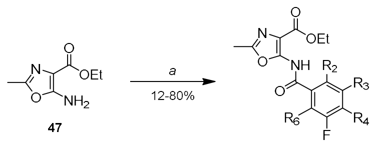

47

25-26

Scheme S8. *Reagents and conditions. a* RCOCl, Et_3N, dichloromethane, 25 °C, 12 h

Ethyl 2-methyl-5-(2,3,4,5-tetrafluorobenzamido)oxazole-4-carboxylate (25)

To a mixture of ethyl 5-amino-2-methyloxazole-4-carboxylate (50 mg, 0.29 mmol, 1 eq.) in DCM (1 mL) was added 2,3,4,5-tetrafluorobenzoyl chloride (62.5 mg, 0.29 mmol, 1 eq.) and TEA (81.8 µL, 0.59 mmol, 2 eq.) at 0 °C, then the mixture was warmed to 25 °C and stirred for 12 hours. LC-MS showed starting material was consumed. The reaction mixture was concentrated under vacuum. The residue was purified by Prep-HPLC (column: Phenomenex Synergi C18 150 x 25 mm x 10 µm; mobile phase: [water (0.225% formic acid)-MeCN]; B%: 35%-65%, 10 min) to obtain the *title compound* (81.2 mg, 235 µmol, 80% yield) as a white solid. ^{1}H NMR (400 MHz, $CDCl_3$) δ = 10.41 - 10.44 (m, 1H), 7.88 - 7.84 (m, 1H), 4.45 (q, *J* = 7.2 Hz, 2H), 2.56(s, 3H), 1.44 (t, *J* = 7.1 Hz, 3H). HRMS (ESI): m/z calcd for $C_{14}H_{11}F_4N_2O_4$ $[M+H^+]$: 347.0649. Found 347.0648.

Ethyl 2-methyl-5-(2,3,4-trifluorobenzamido)oxazole-4-carboxylate (26)

To a mixture of ethyl 5-amino-2-methyloxazole-4-carboxylate (60 mg, 0.35 mmol, 1 eq.) in DCM (2 mL) was added TEA (245 µL, 1.76 mmol, 5 eq.) and 2,3,4-trifluorobenzoyl chloride (137 mg, 0.71 mmol, 2 eq.) at 0 °C, then the mixture was warmed to 25 °C and stirred for 1 hour. LC-MS showed the starting material was consumed. The reaction mixture was concentrated under vacuum. To the mixture was added 250 mg of K_2CO_3 and the resulting mixture was stirred at 50 °C for 30 min and quenched by addition of H_2O (10 mL) at 0 **°C** and extracted with ethyl acetate (10 mL x 3). The combined organic layers were washed with brine (10 mL), dried (Na_2SO_4), filtered and concentrated under reduced pressure to give a residue. The residue was purified by Prep-HPLC (column: Shim-pack C18 150x25x10 µm; mobile phase: [water (0.225%FA)-acetonitrile];B%: 40%-60%, 10min) to give the *title compound* (13.8 mg, 41.5 µmol, 12% yield) as a white solid. ^{1}H NMR (400 MHz, $CDCl_3$) δ 7.90 (dd, *J* = 6.8, 8.4 Hz,

2H), 4.18 (q, J = 7.1 Hz, 2H), 2.44 (s, 3H), 1.16 (t, J = 7.1 Hz, 3H). LC-MS m/z 328.9 $[M+H]^+$; HRMS (ESI): m/z calcd for $C_{14}H_{12}F_3N_2O_4$ $[M+H^+]$: 329.0744. Found 329.0749.

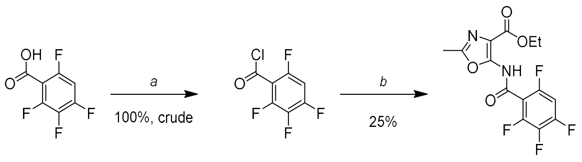

27

Scheme S9. *Reagents and conditions. a* Oxalyl chloride, DMF, DCM, 0 °C; *b* **47**, Et_3N, dichloromethane, 25 °C, 12 h

2,3,4,6-tetrafluorobenzoyl chloride

To a solution of 2,3,4,6-tetrafluorobenzoic acid (100 mg, 0.52 mmol, 1 *eq*.) and DMF (20 µL, 0.26 mmol, 0.5 *eq*.) in DCM (1 mL) was added oxalyl dichloride (68 µL, 0.77 mmol, 1.5 *eq*.) at 0 °C. The mixture was stirred for 12 hours at 12 °C. TLC (PE: EA=1:1) showed a new spot was formed. The mixture was concentrated under vacuum to give the *title compound* (110 mg, crude) as light brown oil, which was used in the next step directly.

Ethyl 2-methyl-5-(2,3,4,6-tetrafluorobenzamido)oxazole-4-carboxylate (27)

To a solution of ethyl 5-amino-2-methyloxazole-4-carboxylate (80 mg, 0.47 mmol, 1 *eq*.) and TEA (196 µL, 1.41 mmol, 3 *eq*.) in DCM (1 mL) was added 2,3,4,6-tetrafluorobenzoyl chloride (100 mg, 0.47 mmol, 1 *eq*.) at 0 °C. The mixture was stirred for 12 hours at 20 °C. LC-MS showed the desired mass was formed. The mixture was concentrated under vacuum and the residue was purified by Prep-HPLC (column: Shim-pack C18 150 mm x 25 mm x 10 µm; mobile phase: [water (0.225% formic acid)-MeCN]; B%: 36%-58%, 10 min) to give the *title compound* (41.1 mg, 118 µmol, 25% yield) as a yellow solid. ^1H NMR (400 MHz, MeOD) δ 7.35 - 7.29 (m, 1H), 4.35 (q, J = 7.1 Hz, 2H), 2.50 (s, 3H), 1.35 (t, J = 7.1 Hz, 3H). HRMS (ESI): m/z calcd for $C_{14}H_{11}F_4N_2O_4$ $[M+H^+]$: 347.0649. Found 347.0657

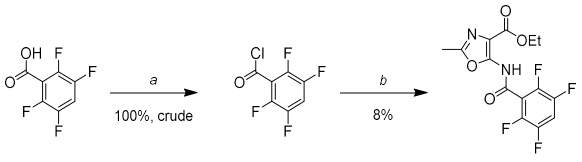

28

Scheme S10. *Reagents and conditions. a* Thionyl chloride, DMF; *b* **47**, Et_3N, dichloromethane, 25 °C, 12 h

2,3,4,5-tetrafluorobenzoyl chloride

To a solution of 2,3,4,6-tetrafluorobenzoic acid (100 mg, 0.52 mmol, 1 *eq*.) and DMF (20 µL, 52 µmol, 0.1 *eq*.) in thionyl chloride (1 mL, 13.78 mmol, 27 eq.) was stirred for 12 hours at 25 °C under N_2. LC-MS showed compound **S37** was consumed. The mixture was concentrated under vacuum to give the *title compound* (230 mg, crude) as a colourless oil, which was used to next step directly.

Step 2: Ethyl 2-methyl-5-(2,3,4,5-tetrafluorobenzamido)oxazole-4-carboxylate (28)

To a solution of ethyl 5-amino-2-methyloxazole-4-carboxylate (60 mg, 0.35 mmol, 1 *eq*.) and TEA (245 µL, 1.76 mmol, 5 *eq*.) in DCM (1 mL) was added 2,3,4,5-tetrafluorobenzoyl chloride (150 mg, 0.71 mmol, 2 *eq*.) at 0 °C. The mixture was stirred for 12 hours at 20 °C. LC-MS showed the desired mass was formed. Potassium carbonate (250 mg, 1.81 mmol, 5.1 eq.) and was stirred at 50°C for 30 minutes. The reaction mixture was cooled to 0°C, quenched with water (10 mL) and extracted with ethyl acetate (3 x 10 mL). The combined organic layers were washed with brine (10 mL), dried over $\mathbf{Na_2SO_4}$, filtered and concentrated under reduced pressure. The residue was purified by Prep-HPLC (column: Shim-pack C18 150 mm x 25 mm x 10 µm; mobile phase: [water (0.225% formic acid)-MeCN]; B%: 36%-58%, 10 min) to give the *title compound* (10.03 mg, 28.7 µmol, 8% yield) as a white solid. 1H NMR (400 MHz,

DMSO) δ 8.12 (br d, J = 17.1 Hz, 1H), 4.21 (d, J = 7.1 Hz, 2H), 2.42 (s, 3H), 1.23 (t, J = 7.1 Hz, 3H). LRMS (ESI) (*m/z*) $[MH]^+$ = 347.0.

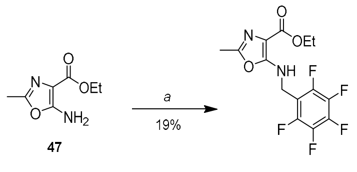

Scheme S11. *Reagents and conditions. a* 1-(bromomethyl)-2,3,4,5,6-pentafluorobenzene, K_2CO_3, DMF, 20 °C, 30 min

Ethyl 2-methyl-5-(((pentafluorophenyl)methyl)amino)oxazole-4-carboxylate (29)

To a mixture of ethyl 5-amino-2-methyloxazole-4-carboxylate (80 mg, 0.47 mmol, 1 *eq.*) in DMF (0.8 mL) was added K_2CO_3 (130 mg, 0.94 mmol, 2 *eq.*) and 1-(bromomethyl)-2,3,4,5,6-pentafluorobenzene (21.7 µL, 0.38 mmol, 0.8 *eq.*), then the mixture was stirred at 20 °C for 0.5 hours under N_2. LC-MS showed the starting material was consumed. The reaction mixture was quenched by addition of water (8 mL) and extracted with ethyl acetate (4 x 10 mL). The combined organic layers were washed with brine (15 mL), dried over Na_2SO_4, filtered and concentrated under reduced pressure. The residue was purified by prep-HPLC (column: Phenomenex Synergi C18 150 mm x 25 mm x 10 µm; mobile phase: [water (0.225% formic acid)-MeCN]; B%: 48%-78%, 8.5 min) to give the *title compound* (30.6 mg, 87.3 µmol, 19% yield) as a white solid. ^1H NMR (400 MHz, MeOD): δ 4.70 (s, 2H), 4.26 (q, *J*=7.1 Hz, 2H), 2.32 (s, 3H), 1.31 (t, *J*=7.1Hz, 3H). LRMS (ESI) (*m/z*) $[MH]^+$ = 350.9

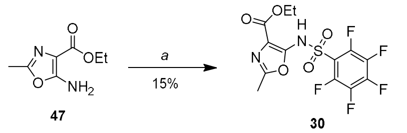

Scheme S12. *Reagents and conditions. a* Pentafluorobenzenesulfonyl chloride, Et_3N, dichloromethane, 20 °C, 14 h.

Ethyl 2-methyl-5-((pentafluorophenyl)sulfonamido)oxazole-4-carboxylate (30)

and TEA (122 µL, 0.88 mmol, 3 eq.) in DCM (2 mL) was added drop-wise 2,3,4,5,6-pentafluorobenzenesulfonyl chloride (47.6 µL, 0.32 mmol, 1.1 eq.) at 0 °C. Then the solution was stirred at 20 °C for 14 hours. LC-MS showed most of the starting material was consumed. The reaction mixture was concentrated under vacuum. The crude product was purified by Prep-HPLC (column: Phenomenex Gemini NX-C18 (75 x 30 mm x 3 µm); mobile phase: [water (10 mM NH_4HCO_3)-MeCN]; B%: 15%-35%, 8 min) to obtain the *title compound* (19.1 mg, 43 µmol, 15% yield, 90% purity) as a light yellow solid. ^1H NMR (400 MHz, MEOD) δ 4.18 (q, J = 7.2 Hz, 2H), 2.24 (s, 3H), 1.27 (t, J = 7.2 Hz, 3H). LRMS (ESI) (*m/z*) $[MH]^+$ = 400.9.

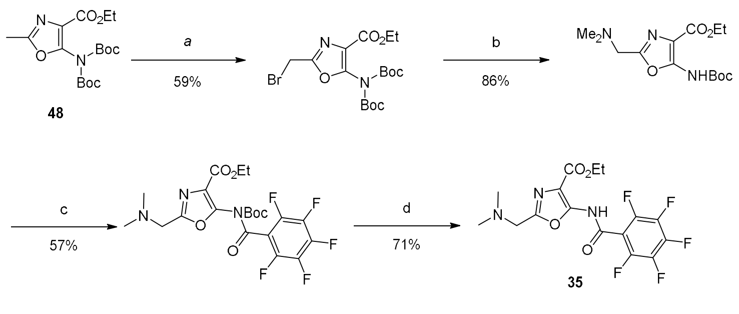

Scheme S13. *Reagents and conditions. a* NBS, AIBN, CCl_4, 70 °C, 12 h; *b* dimethylamine, THF, 70 °C, 15 min; *c* pentafluorobenzoyl chloride, Et_3N, dichloromethane, 20-25 °C; *d* trifluoroacetic acid, dichloromethane

Ethyl 5-(bis(*tert*-butoxycarbonyl)amino)-2-(bromomethyl)oxazole-4-carboxylate

To a mixture of ethyl 5-(bis(tert-butoxycarbonyl)amino)-2-methyloxazole-4-carboxylate (500 mg, 1.35 mmol, 1 *eq.*) in carbon tetrachloride (10 mL) was added NBS (360 mg, 2.02 mmol, 1.5 *eq.*) and AIBN (222 mg, 1.35 mmol, 1 *eq.*) in one portion at 20 °C under N_2. The mixture was stirred at 70 °C for 12 hours. LC-MS showed the starting material was consumed. The reaction was filtered and the filtrate was concentrated in vacuum. The residue was purified by column chromatography (SiO_2, EtOAc/Petroleum ether 1-90%)) to give the *title compound* (430 mg, 0.79 mmol, 59% yield, 83% purity) as a colourless oil. ^{1}H NMR (400 MHz, $CDCl_3$) δ 4.46 (s, 2H), 4.39 (q, *J*=7.2 Hz, 2H), 1.46 (s, 18H), 1.38 (t, *J*=7.2 Hz, 3H). LRMS (ESI) (*m/z*) $[M – 2 Boc + H]^+$ = 249 /251

Ethyl 5-((*tert*-butoxycarbonyl)amino)-2-((dimethylamino)methyl)oxazole-4-carboxylate

A mixture of ethyl 5-(bis(*tert*-butoxycarbonyl)amino)-2-(bromomethyl)oxazole-4-carboxylate (150 mg, 0.33 mmol, 1 *eq.*) in dimethylamine solution (2 M in THF, 3.75 mL, 22 *eq.*) was stirred for 15 minutes at 70 °C. LC-MS showed the starting material was consumed. The reaction mixture was concentrated in vacuum. The crude product was purified by prep-TLC (SiO_2 EtOAc/Petroleum ether 1:2) to give the *title compound* (90 mg, 0.29 mmol, 86% yield) as a yellow solid. ^{1}H NMR (400 MHz, $CDCl_3$) δ 8.35 (s, 1H), 4.32 (q, *J* = 7.1 Hz, 2H), 3.56 (s, 2H), 2.28 (s, 6H), 1.46 (s, 9H), 1.33 (t, *J* = 7.2 Hz, 3H).

Ethyl 5-(N-(*tert*-butoxycarbonyl)-2,3,4,5,6-pentafluorobenzamido)-2-((dimethylamino)methyl) oxazole-4-carboxylate

To a mixture of ethyl 5-((*tert*-butoxycarbonyl)amino)-2-((dimethylamino)methyl)oxazole-4-carboxylate (60 mg, 0.19 mmol, 1 *eq.*) and TEA (133 µL, 0.96 mmol, 5 *eq.*) in DCM (1 mL) was added 2,3,4,5,6-pentafluorobenzoyl chloride (26.4 µL, 0.19 mmol, 1 *eq.*) at 20 °C , then the mixture was stirred for 15 min at 20 °C. TLC (PE: EA=1:1) showed the starting material was consumed. The reaction mixture was concentrated in vacuum. The crude product purified by prep-TLC (SiO_2, EtOAc/Petroleum ether 1:1) to give the *title compound* (65 mg, 0.11 mmol, 57% yield, 85% purity) as a yellow solid. ^{1}H NMR (400 MHz, $CDCl_3$) δ 4.39 (q, *J* = 7.2 Hz, 2H), 3.74 (s, 2H), 2.47 - 2.29 (m, 6H), 1.35 (br t, *J* = 7.2 Hz, 12H). LRMS (ESI) (*m/z*) $[MH]^+$ = 507.9

Ethyl 2-((dimethylamino)methyl)-5-(pentafluorobenzamido)oxazole-4-carboxylate

A mixture of ethyl 5-(N-(*tert*-butoxycarbonyl)-2,3,4,5,6-pentafluorobenzamido)-2-((dimethylamino)methyl)oxazole-4-carboxylate (60 mg, 0.12 mmol, 1 *eq.*) and TFA (1.50 mL, 20 mmol, 171 *eq.*) in DCM (1 mL) was stirred for 15 min at 20 °C. LC-MS showed the starting material was consumed. The reaction mixture was concentrated in vacuum. The crude product was purified by prep-HPLC (column: Phenomenex luna C18 150 mm x 25 mm x 10 µm; mobile phase: [water (0.225% formic acid)-MeCN]; B%: 10%-40%, 9min), then the product in aq. $NaHCO_3$(1M, 5 mL) was extracted with ethyl acetate (3 x 10 mL). The combined organic phase was washed with brine (20 mL), dried with anhydrous Na_2SO_4, filtered and concentrated in vacuum to give the *title compound* (34.4 mg, 83.6 µmol, 71% yield) as a yellow solid. ^{1}H NMR (400 MHz, MeOD) δ 4.21 (q, *J* = 7.1 Hz, 2H), 3.54 (s, 2H), 2.30 - 2.21 (m, 6H), 1.23 (t, *J* = 7.2 Hz, 3H). LRMS (ESI) (*m/z*) $[MH]^+$ = 408.0

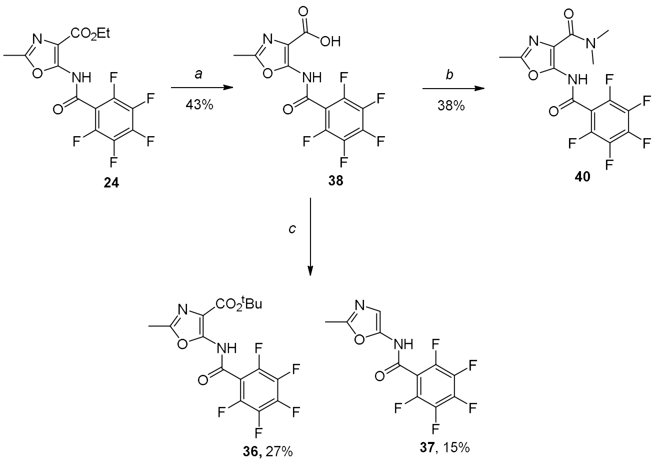

Scheme S14. *Reagents and conditions. a* NaOH, water, EtOH, 50 °C; *b* dimethylamine hydrochloride, HATU, *i*-Pr_2NEt, DMF, 25 °C; *c tert*-butyl 2,2,2-trichloroacetimidate, BF_3•OEt_2, toluene, 90 °C.

2-Methyl-5-(pentafluorobenzamido)oxazole-4-carboxylic acid (38)

The reaction mixture of ethyl 2-methyl-5-(perfluorobenzamido)oxazole-4-carboxylate **24**(450 mg, 1.24 mmol, 1 *eq.*) and aq. NaOH (1 M, 4.94 mL, 4 *eq.*) in EtOH (4 mL) was stirred at 50 °C for 2 hours. LC-MS showed desired compound. The mixture was concentrated under vacuum and the residue was purified by prep-HPLC (column: Phenomenex Synergi C18 150 x 25 mm x 10 µm; mobile phase: [water (0.225% formic acid)-MeCN]; B%: 18%-51%, 11 min) to give the *title compound* (159 mg, 0.47 mmol, 38% yield) as white solid. ^{1}H NMR (400 MHz, MeOD) δ 2.49 (s, 3H).); HRMS (ESI): m/z calcd for $C_{12}H_6F_5N_2O_4$ [M+H$^+$]: 337.0242. Found 337.0236

***tert*-Butyl 2-methyl-5-(perfluorobenzamido)oxazole-4-carboxylate (36)**

A mixture of 2-Methyl-5-(pentafluorobenzamido)oxazole-4-carboxylic acid (100 mg, 0.30 mmol, 1 *eq.*) and *tert*-butyl 2,2,2-trichloroacetimidate (107 µL, 0.59 mmol, 2 *eq.*) in toluene (1 mL) was stirred at 90 °C for 12 hours. LC-MS showed that the starting material was consumed. The reaction mixture was diluted with ethyl acetate (5 mL x 3). The organic layer was washed with brine (10 mL), dried over Na_2SO_4, filtered and the filtrate was concentrated. The crude product was purified by prep-HPLC (column: Phenomenex Synergi C18 150 x 25 mm x 10 µm; mobile phase: [water (0.225% formic acid)-MeCN]; B%: 40%-70%, 10 min) to obtain the *title compound* (31.7 mg, 80.8 µmol, 27% yield) as a white solid. ^{1}H NMR (400 MHz, MeOD) δ 2.47 (s, 3H), 1.54 (s, 9H). LRMS (ESI) (*m/z*) [M+H-*t*-Bu]$^+$ = 337.0

2,3,4,5,6-Pentafluoro-*N*-(2-methyloxazol-5-yl)benzamide (37)

To the stirring mixture of 2-Methyl-5-(pentafluorobenzamido)oxazole-4-carboxylic acid (80 mg, 0.24 mmol, 1 *eq.*) in toluene (1 mL) was added BF_3•Et_2O (1.47 µL, 12 µmol, 0.05 *eq.*) and *tert*-butyl 2,2,2-trichloroacetimidate (85.3 µL, 0.48 mmol, 2 *eq.*) at 25 °C. The reaction was stirred at 90 °C for 18 hours. LC-MS showed that the starting material was consumed and the desired mass was found. The reaction mixture was diluted with ethyl acetate (3 x 5 mL). The organic layer was washed with brine (10 mL), dried over Na_2SO_4, filtered and the filtrate was concentrated. The crude product was purified by Prep-HPLC (column: Phenomenex Synergi C18 150 x 25 mm x 10 µm; mobile phase: [water (0.225% formic acid)-MeCN]; B%: 25%-55%, 10 min) to obtain the *title compound* (11.7 mg, 36.8 umol, 15% yield) as a white solid. ^{1}H NMR (400 MHz, MeOD) δ 7.01 (s, 1H), 2.43 (s, 3H); HRMS (ESI): m/z calcd for $C_{11}H_6F_5N_2O_2$ [M+H$^+$]: 293.0344. Found 293.0327

***N,N*,2-trimethyl-5-(pentafluorobenzamido)oxazole-4-carboxamide (40)**

To the stirring solution of 2-Methyl-5-(pentafluorobenzamido)oxazole-4-carboxylic acid (100 mg, 0.30 mmol, 1 eq.), DIEA (155 µL, 0.89 mmol, 3 eq.) and HATU (136 mg, 0.36 mmol, 1.2 eq.) in DMF (1 mL) was added dimethylamine hydrochloride (24 mg, 0.30 mmol, 1 eq.). Then the mixture was stirred at

25 °C for 1 hour. LC-MS showed most of the starting material was consumed and the desired mass was formed. The reaction mixture was poured into sat. aq. NH_4Cl (5 mL) and extracted with ethyl acetate (3 x 2 mL). The combined organic layer was washed with brine (5 mL), dried over Na_2SO_4, filtered and the filtrate was concentrated. The crude product was purified by Prep-HPLC (column: Phenomenex Synergi C18 150 x 25 mm x 10 µm; mobile phase: [water (0.225% formic acid)-MeCN]; B%: 35%-65%, 10 min) to obtain the *title compound* (34.0 mg, 89.1 µmol, 30% yield) as a gray solid. ^1H NMR (400 MHz, DMSO): δ 11.64 (br.s, 1H), 3.12 (m, 3H), 2.92 (m, 3H), 2.40 (m, 3H).; HRMS (ESI): m/z calcd for $C_{14}H_{11}F_5N_3O_3$ [M+H$^+$]: 364.0715. Found 364.0723

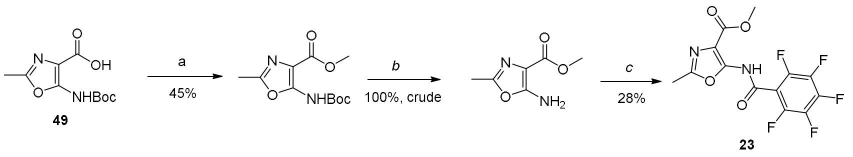

Scheme S15. *Reagents and conditions. a* TMSCHN$_2$, MeOH, Toluene, 0ºC, 1h; *b* trifluoroacetic acid, dichloromethane; *c* pentafluorobenzoyl chloride, Et$_3$N, dichloromethane, 20-25 °C

Methyl 5-((*tert*-butoxycarbonyl)amino)-2-methyloxazole-4-carboxylate

To a solution of 5-((tert-butoxycarbonyl)amino)-2-methyloxazole-4-carboxylic acid (400 mg, 1.65 mmol, 1 *eq.*) in toluene (2 mL) and MeOH (2 mL) was added trimethylsilyldiazomethane solution (2 M in n-hexane, 908 uL, 1.1 *eq*) in dropwise at 0 ° C. The mixture was stirred for 30 min at 0 °C. TLC (1:1 EtOAc/petroleum ether) showed a new spot. Acetic acid (0.2 mL) was slowly added to the mixture and the mixture was concentrated under vacuum. The residue was purified by flash silica gel chromatography (0-50% EtOAc-petroleum ether) to give the *title compound* (190 mg, 0.74 mmol, 45% yield) as a white solid. ^1H NMR (400 MHz, $CDCl_3$) δ 8.35 (br s, 1H), 3.91 (s, 3H), 2.47 (s, 3H), 1.54 (s, 9H).

Methyl 5-amino-2-methyloxazole-4-carboxylate

To a solution of methyl 5-((*tert*-butoxycarbonyl)amino)-2-methyloxazole-4-carboxylate (180 mg, 0.70 mmol, 1 *eq.*) in DCM (3 mL) was added TFA (1 mL, 13.5 mmol). The mixture was stirred for 30 min at 20 °C. TLC (1:1 EtOAc/petroleum ether) showed the starting material was consumed. Sat. aq. $NaHCO_3$ (15mL) was added to the mixture and extracted with EtOAc (3 × 20 mL). The combined organic layers were washed with brine (3 × 10 mL), dried over Na_2SO_4, filtered and concentrated under reduced pressure to give the *title compound* (113 mg, crude) as a white solid which was used in the next step without purification. ^1H NMR (400 MHz, $CDCl_3$) δ 5.31 (br s, 2H), 3.86 (s, 3H), 2.34 (s, 3H).

Methyl 2-methyl-5-(perfluorobenzamido)oxazole-4-carboxylate (23)

A mixture of methyl 5-amino-2-methyloxazole-4-carboxylate (80 mg, 0.51 mmol, 1 *eq.*), 2,3,4,5,6-pentafluorobenzoyl chloride (70.7 µL, 0.51 mmol, 1 *eq.*) and TEA (155 mg, 1.54 mmol, 3 *eq*) in DCM (2 mL) was stirred for 12 hours at 20 ° C. LC-MS showed the desired mass. The mixture was concentrated under vacuum and the residue was purified by prep-HPLC (column: Shim-pack C18 150x25x10 µm; mobile phase: [water (0.225% FA)-acetonitrile]; B%: 38%-58%, 9 min) to give the *title compound* (51.9 mg, 144 µmol, 28% yield) as a white solid. ^1H NMR (400 MHz, $CDCl_3$) δ 3.87 (s, 3H), 2.49 (s, 3H). LRMS (ESI) (*m/z*) [MH]$^+$ = 350.9

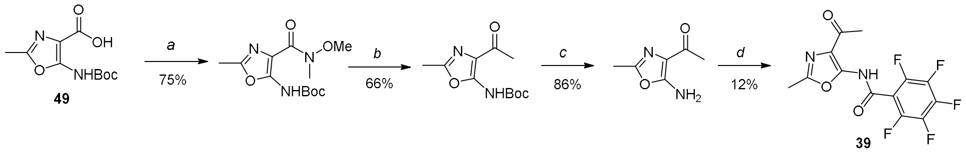

Scheme S16. *Reagents and conditions. a N,O*-dimethylhydroxylamine hydrochloride, EDCI, HOBt, Et_3N, dichloromethane, 25 °C; *b* MeMgBr, THF, 0 °C; *c* trifluoroacetic acid, dichloromethane; *d* pentafluorobenzoyl chloride, Et_3N, dichloromethane, 20-25 °C

***tert*-butyl (4-(methoxy(methyl)carbamoyl)-2-methyloxazol-5-yl)carbamate**

The reaction mixture of 5-((tert-butoxycarbonyl)amino)-2-methyloxazole-4-carboxylic acid (500 mg, 2.06 mmol, 1 eq.), N-methoxymethanamine hydrochloride (241 mg, 2.48 mmol, 1.2 eq.), EDCI (791 mg, 4.13 mmol, 2 eq.), HOBt (558 mg, 4.13 mmol, 2 eq.) and TEA (1.15 mL, 8.26 mmol, 4 eq.) in DCM (5 mL) was stirred at 25 °C for 12 hours. LC-MS showed the starting material was consumed. The reaction mixture was poured into sat. aq. NH_4Cl (5 mL) and extracted with DCM (2 x 5 mL). The combined organic layer was washed with brine (15 mL), dried over Na_2SO_4, filtered and the filtrate was concentrated. The residue was purified by column chromatography (SiO_2, PE: EA = 10:1 -2:1) to obtain the *title compound* (450 mg, 1.55 mmol, 75% yield) as a white solid. ^1H NMR (400 MHz, $CDCl_3$) δ 9.25 (s, 1H), 3.82 (s, 3H), 3.48 (s, 3H), 2.46 (s, 3H), 1.51 (s, 9H). LRMS (ESI) (*m/z*) $[M+Na]^+$ =308.1

***tert*-butyl (4-acetyl-2-methyloxazol-5-yl)carbamate**

To a suspension of *tert*-butyl (4-(methoxy(methyl)carbamoyl)-2-methyloxazol-5-yl)carbamate (200 mg, 0.70 mmol, 1 eq.) in THF (10 mL) was added MeMgBr (3 M in THF, 0.70 mL, 3 eq.) at 0 °C. The solution was stirred at 0 °C for 2 hours. TLC (PE: EA=1:1) showed most of the starting material (Rf=0.4) was consumed and a new spot (Rf=0.7) was formed. The reaction mixture was poured into sat. aq. NH_4Cl (5 mL) and extracted with ethyl acetate (3 x 2 mL). The combined organic layer was washed with brine (5 mL), dried over Na_2SO_4, filtered and the filtrate was concentrated. The residue was purified by column chromatography (SiO_2, PE: EA = 10:1 -2:1) to obtain the *title compound* (120 mg, 0.46 mmol, 66% yield, 93% purity) as a white solid. ^1H NMR (400 MHz, $CDCl_3$) δ 9.09 (br s, 1H), 2.48 (s, 3H), 2.46 (s, 3H), 1.54 (s, 9H). LRMS (ESI) (*m/z*) $[M+Na]^+$ =263.0

1-(5-amino-2-methyloxazol-4-yl)ethan-1-one

The reaction mixture of *tert*-butyl (4-acetyl-2-methyloxazol-5-yl)carbamate (100 mg, 0.42 mmol, 1 eq.) in TFA (0.4 mL) and DCM (1.2 mL) was stirred at 25 °C for 2 hours. TLC (PE: EA=1:1) showed the starting material (Rf=0.6) was consumed and a new spot (Rf=0.4) was formed. The reaction mixture was poured into aq. K_2CO_3 (10 mL) and extracted with DCM (3 x 5 mL). The combined organic layer was washed with brine (15 mL), dried over Na_2SO_4, filtered and the filtrate was concentrated to obtain the *title compound* (50 mg, 0.36 mmol, 86% yield) as a white solid. ^1H NMR (400 MHz, $CDCl_3$) δ 5.87 (br. s, 2H), 2.40 (s, 3H), 2.35 (s, 3H). LRMS (ESI) (*m/z*) $[MH]^+$= 141.0

***N*-(4-Acetyl-2-methyloxazol-5-yl)-2,3,4,5,6-pentafluorobenzamide (39)**

To the reaction mixture of 1-(5-amino-2-methyloxazol-4-yl)ethan-1-one (50 mg, 0.36 mmol, 1 eq.) and TEA (149 µL, 1.07 mmol, 3 eq.) in DCM (1 mL) was added 2,3,4,5,6-pentafluorobenzoyl chloride (54 µL, 0.39 mmol, 1.1 eq) at 0 °C. Then the solution was stirred at 25 °C for 12 hours. LC-MS showed the starting material was consumed. The reaction mixture was concentrated under vacuum. The crude product was purified by Prep-HPLC (column: Phenomenex Synergi C18 150 x 25 mm x 10 µm; mobile phase: [water (0.225% formic acid)-MeCN]; B%: 38%-68%, 8.5 min) to obtain the *title compound* (14.2 mg, 42.6 µmol, 12% yield) as a white solid. ^1H NMR (400 MHz, MeOD) δ 2.49 (s, 3H), 2.47 (s, 3H). HRMS (ESI): m/z calcd for $C_{13}H_8F_5N_2O_3$ $[M+H^+]$: 335.0450. Found 335.0456

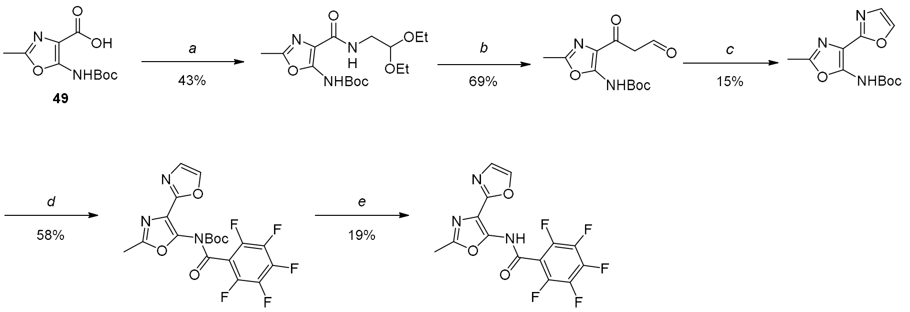

Scheme S17. *Reagents and conditions. a* 2,2-diethoxyethanamine, HATU, Et_3N, DMF; *b* aq. HCl, THF, 15 °C; *c* Burgess reagent, toluene, 70 °C; *d* pentafluorobenzoyl chloride, Et_3N, dichloromethane, 20-25 °C; *e* trifluoroacetic acid, dichloromethane

***tert*-Butyl (4-((2,2-diethoxyethyl)carbamoyl)-2-methyloxazol-5-yl)carbamate**

The reaction mixture of 5-((tert-butoxycarbonyl)amino)-2-methyloxazole-4-carboxylic acid (0.50 g, 2.06 mmol, 1 eq.), 2,2-diethoxyethanamine (600 µL, 4.13 mmol, 2 eq.), HATU (1.18 g, 3.10 mmol, 1.5 eq.) and triethylamine (0.86 mL, 6.2 mmol, 3 eq) in DMF (5 mL) was stirred at 15 °C for 14 hours. LC-MS showed the starting material was consumed and the desired mass was given. The reaction mixture was poured into sat. aq. NH_4Cl (15 mL) and extracted with ethyl acetate (3 x 10 mL). The combined organic layer was washed with brine (15 mL), dried over Na_2SO_4, filtered and the filtrate was concentrated. The residue was purified by column chromatography (SiO_2, PE: EA = 10:1 -2:1) to obtain the *title compound* (330 mg, 0.88 mmol, 43% yield, 95% purity) as a colourless oil. ^1H NMR (400 MHz, $CDCl_3$) δ 8.68 (s, 1H), 6.75 (br. t, *J*=5.5 Hz, 1H), 4.56 (t, *J*=5.5 Hz, 1H), 3.74 (qd, *J*=7.1, 9.4 Hz, 2H), 3.58 (qd, *J*=7.0, 9.4 Hz, 2H), 3.51 (t, *J*=5.8 Hz, 2H), 2.45 (s, 3H), 1.52 (s, 9H), 1.24 (t, *J*=7.0 Hz, 6H). LRMS (ESI) (*m/z*) $[MH]^+$ =358.1

***tert*-butyl (2-methyl-4-((2-oxoethyl)carbamoyl)oxazol-5-yl)carbamate**

The reaction mixture of *tert*-Butyl (4-((2,2-diethoxyethyl)carbamoyl)-2-methyloxazol-5-yl)carbamate (0.42 g, 1.18 mmol, 1 eq.) in THF (8 mL) and aq. HCl (1 M, 5.88 mL, 5 eq) was stirred at 15 °C for 14 hours. The reaction mixture was poured into sat. aq. $NaHCO_3$ (15 mL) and extracted with EA (3 x 10 mL). The combined organic layer was washed with brine (5 mL), dried over Na_2SO_4, filtered and the filtrate was concentrated. The residue was purified by column chromatography (SiO_2, PE: EA = 10:1 - 0:1) to obtain the *title compound* (230 mg, 0.81 mmol, 69% yield) as a white solid. LRMS (ESI) (*m/z*) $[MH]^+$ =*m/z* 284.1

***tert*-butyl (2'-methyl-[2,4'-bioxazol]-5'-yl)carbamate**

The reaction mixture of *tert*-butyl (2-methyl-4-((2-oxoethyl)carbamoyl)oxazol-5-yl)carbamate (230 mg, 0.81 mmol, 1 eq.) and Burgess reagent (580 mg, 2.44 mmol, 3 eq.) in toluene (4 mL) was stirred at 70 °C for 14 hrs. LC-MS showed the starting material was consumed and the desired mass was given. The reaction mixture was concentrated under vacuum. The residue was purified by column chromatography (SiO_2, PE: EA = 50:1 -10:1) to obtain the *title compound* (40 mg, 0.12 mmol, 15% yield, 80% purity) as a white solid. LRMS (ESI) (*m/z*) $[MH]^+$ =266.0

***tert*-butyl (2'-methyl-[2,4'-bioxazol]-5'-yl)(pentafluorobenzoyl)carbamate**

To the reaction mixture of *tert*-butyl (2'-methyl-[2,4'-bioxazol]-5'-yl)carbamate (30 mg, 0.11 mmol, 1 eq.) and TEA (47 µL, 39.28 umol, 47.22 uL, 3 eq.) in DCM (0.5 mL) was added 2,3,4,5,6-pentafluorobenzoyl chloride (19 µL, 0.14 mmol, 1.2 eq.) at 0 °C. Then the solution was stirred at 15 °C for 12 hours. TLC (PE: EA=1:1) showed the starting material (Rf=0.2) was consumed and a new spot (Rf=0.5) was found. The reaction mixture was concentrated under vacuum. The crude product was purified by Prep-TLC (PE: EA = 1:1) to the *title compound* (30 mg, 65 µmol, 58% yield) as a white solid. LRMS (ESI) (*m/z*) $[MH]^+$ =460.0

2,3,4,5,6-pentafluoro-*N*-(2'-methyl-[2,4'-bioxazol]-5'-yl)benzamide (41)

The reaction mixture of *tert*-butyl (2'-methyl-[2,4'-bioxazol]-5'-yl)(pentafluorobenzoyl)carbamate (30 mg, 65 µmol, 1 eq.) in TFA (0.1 mL) and DCM (0.3 mL) was stirred at 15 °C for 2 hours. LC-MS showed the starting material was consumed and the desired mass was given. The reaction mixture was concentrated under vacuum. The crude product was purified by Prep-HPLC (column: Shim-pack C18 150 mm x 25 mm x 10 µm; mobile phase: [water (0.225% formic acid)-MeCN];B%: 36%-56%,10 min) to obtain the *title compound* (4.63 mg, 12.3 µmol, 19% yield, 95% purity) as a white solid. ^{1}H NMR (400 MHz, MeOD) δ 7.98 (s, 1H), 7.31 (s, 1H), 2.53 (s, 3H). HRMS (ESI): m/z calcd for $C_{14}H_7F_5N_3O_3$ [M+H$^+$]: 360.0402. Found 360.0422

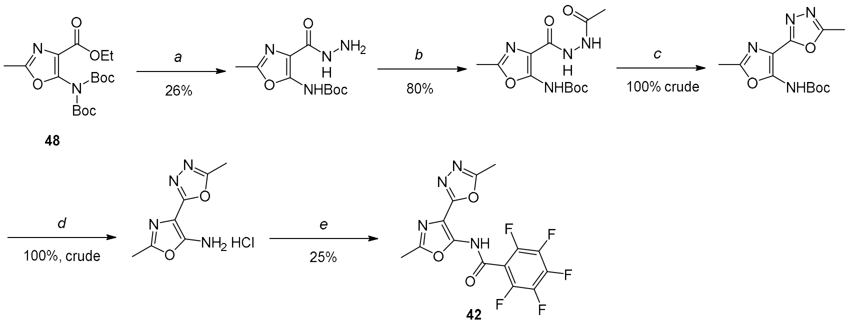

Scheme S18. *Reagents and conditions. a* hydrazine hydrate, acetonitrile, 25 °C; *b* acetic anhydride, Et_3N, methanol, 25 °C; *c* iodine, triphenylphosphine, Et_3N, dichloromethane, 25 °C; *d* HCl/dioxane; *e* pentafluorobenzoyl chloride, Et_3N, dichloromethane

***tert*-butyl (4-(hydrazinecarbonyl)-2-methyloxazol-5-yl)carbamate**

A solution of ethyl 5-(bis(tert-butoxycarbonyl)amino)-2-methyloxazole-4-carboxylate (600 mg, 1.62 mmol, 1 *eq.*) and hydrazine hydrate (3 mL, 60 mmol, 37 *eq.*) in acetonitrile (2 mL) was stirred at 25 °C for 12 h. LC-MS *m/z* and TLC (1:1 EtOAc/petroleum ether) showed the reaction was completed. The residue was poured into ice-water (20 mL) and stirred for 5 min. The aqueous phase was extracted with ethyl acetate (5 x 10 mL). The combined organic phase was washed with brine (30 mL), dried with anhydrous Na_2SO_4, filtered and concentrated in vacuum to give the crude product. The residue was purified by column chromatography (SiO_2, EtOAc/Petroleum ether 25-100%) to obtain the *title compound* (310 mg, 0.85 mmol, 26% yield, 70% purity) as a yellow solid. ^{1}H NMR (400 MHz, $CDCl_3$) δ 8.48 (br s, 1H), 7.71 (br s, 1H), 3.89 (br s, 2H), 2.37 (s, 3H), 1.45 (s, 9H). LRMS (ESI) (*m/z*) [M+Na]$^+$ = 279.0

***tert*-butyl (4-(2-acetylhydrazine-1-carbonyl)-2-methyloxazol-5-yl)carbamate**

To a solution of *tert*-butyl (4-(hydrazinecarbonyl)-2-methyloxazol-5-yl)carbamate (100 mg, 0.390 mmol, 1 *eq.*) and acetic anhydride (73 µL, 0.78 mmol, 2 *eq.*) in methanol (2 mL) was added TEA (0.27 mL, 1.95 mmol, 5 *eq.*) at 0 °C. The solution was stirred at 25 °C for 12 h. LC-MS and TLC (PE: EA=1:1) showed the reaction was completed. The residue was poured into ice-water (20 mL) and stirred for 5 min. The aqueous phase was extracted with ethyl acetate (5 x 10 mL). The combined organic phase was washed with brine (30 mL), dried with anhydrous Na_2SO_4, filtered and concentrated in vacuum to give the crude product. The residue was purified by column chromatography (SiO_2, EtOAc/Petroleum ether 25-100%)) to the *title compound* (100 mg, 0.31 mmol, 80% yield, 93% purity) as a yellow solid. LRMS (ESI) (*m/z*) [MH]$^+$ = 299.1

***tert*-butyl (2-methyl-4-(5-methyl-1,3,4-oxadiazol-2-yl)oxazol-5-yl)carbamate**

To a solution of *tert*-butyl (4-(2-acetylhydrazine-1-carbonyl)-2-methyloxazol-5-yl)carbamate (100 mg, 0.33 mmol, 1 *eq.*), triphenylphosphine (88 mg, 0.33 mmol, 1 *eq.*) and TEA (0.14 mL, 1.0 mmol, 3 *eq.*) in DCM (2 mL) was added iodine (67.5 µL, 0.33 mmol, 1 *eq.*) at 0 °C. The solution was stirred at 25 °C for 2 h. LC-MS showed the reaction was complete. The reaction mixture was poured into ice-water

(20 mL) and stirred for 5 min. The aqueous phase was extracted with ethyl acetate (5 x 10 mL). The combined organic phase was washed with brine (30 mL), dried with anhydrous Na_2SO_4, filtered and concentrated in vacuum to give the crude product. The residue was purified by column chromatography (SiO_2, EtOAc/Petroleum ether 25-100%) to give the *title compound* (70 mg, 0.25 mmol, 75% yield) as a yellow solid. LC-MS *m/z* and 1H NMR showed that the product was contaminated with triphenylphosphine oxide. This material was used in the next step without further purification. ^{1}H NMR (400 MHz, $CDCl_3$) δ 8.03 (br s, 1H), 2.53 (s, 3H), 2.45 (s, 3H), 1.47 (s, 9H). LRMS (ESI) (*m/*z) $[MH]^+$ = 281.1

2-methyl-4-(5-methyl-1,3,4-oxadiazol-2-yl)oxazol-5-amine hydrochloride

To give *tert*-butyl (2-methyl-4-(5-methyl-1,3,4-oxadiazol-2-yl)oxazol-5-yl)carbamate (70 mg, 0.25 mmol, 1 *eq.*) in dioxane (1 mL) was added HCl/dioxane (4 M, 875 µL, 14 *eq.*) at 0 °C. The solution was stirred at 25 °C for 1 h. LC-MS showed the reaction was complete. The mixture was filtered and concentrated in vacuum to give the *title compound* (55mg, crude) as a yellow solid. The crude product was used directly in the next step without further purification. LRMS (ESI) (*m/*z) $[MH]^+$ = 181.1

2,3,4,5,6-pentafluoro-*N*-(2-methyl-4-(5-methyl-1,3,4-oxadiazol-2-yl)oxazol-5-yl)benzamide (42)

To a solution of 2-methyl-4-(5-methyl-1,3,4-oxadiazol-2-yl)oxazol-5-amine hydrochloride (55 mg, 0.25 mmol, 1 *eq.*) and TEA (106 µL, 0.76 mmol, 3 *eq.*) in DCM (2 mL) was added 2,3,4,5,6-pentafluorobenzoyl chloride (35 µL, 0.25 mmol, 1 *eq.*) at 0 °C. The solution was stirred at 25 °C for 1 h. LC-MS showed the reaction was complete. The mixture was concentrated in vacuum to give the crude product. The residue was purified by prep-HPLC (column: UniSil 3-100 C18 Ultra (150 mm x 25 mm x 3 µm); mobile phase: [water (0.225% formic acid)-MeCN];B%: 28%-58%,10min). The *title compound* was obtained as yellow solid (25.2 mg, 62.7 µmol, 25% yield, 93% purity). ^{1}H NMR (400 MHz, $CDCl_3$) δ 9.67 (br.s, 1H), 2.61 (s, 3H), 2.60 (s, 3H). HRMS ES^+ 375.0502 $[M + H]^+$, $C_{14}H_8F_5N_4O_3$ requires 375.0511.

Non-Chemistry supplementary methods

GSH adduct formation

Test compound (10 µM) was incubated with 0.5 mg/mL Human Liver Microsomes (Mixed Gender, Life Technologies) in 50 mM potassium phosphate buffer, pH7.4 in the presence of either NADPH (8mg/mL), glutathione or NADPH plus glutathione. Reactions are started by addition of 5 µL of 1 mM test compound (final concentration 10 µM, 1% solvent). Immediately, an aliquot (100 µL) of the incubation mixture was removed and mixed with acetonitrile (200 µL) to stop the reaction. A further 100 uL aliquot is removed at 90 minutes. After addition of 100 µL of water, the samples were centrifuged to sediment precipitated protein and the plates then sealed prior to analysis using a Xevo QTof Quadrupole Time-of-Flight Mass Spectrometer (Waters corporation, USA). Detection of metabolites is performed by analysis with MetabolynxXS and by manual data searching. Elemental composition of the parent ion and MS/MS analysis is used to determine the nature of the GSH adduct. Incubations with Clozapine were performed to provide a positive control.

Met ID in human liver microsomes

Sample Preparation: Test compound (10 µM) was incubated with 0.5 mg/mL Human Liver Microsomes (Mixed Gender, Life Technologies) in 50 mM potassium phosphate buffer, pH7.4 in the presence of cofactor (either NADPH (8mg/mL), glutathione or NADPH plus glutathione). Reactions are started by addition of 12 µL of 1 mM test compound (final concentration 10 µM, 1% solvent) to 1188 uL of incubation mix containing human liver microsomes and cofactor. Immediately, an aliquot (75 µL) of the incubation mixture was removed and mixed with acetonitrile (150 µL) to stop the reaction. A further 75 uL aliquot was removed at 3, 6, 9, 15, 30, 60 and 90 minutes. After addition of 75 µL of water, the samples were centrifuged to sediment precipitated protein and the plates then sealed prior to analysis. Elemental composition of the parent ion and MS/MS analysis is used to determine the nature of the GSH adduct. Incubations with Clozapine were performed to provide a positive control.

Analysis was performed on a Waters Acquity H-Class UHPLC system equipped with a photodiode array detector and and Waters Xevo Q-ToF high resolution mass spectrometer. Test samples and reference standards were run on the UHPLC on a gradient elution profile using the conditions below. After the UV detector the mobile phase flow was analysed using the QToF mass spectrometer. Samples were initially run in the data dependent MSe™ mode where parent ions are collected, and then fragmented using a graded ms/ms collision energy profile. Where necessary the parent and the identified metabolites were run on a targeted ms/ms experiment where only selected ions were selected in the quadrupole and fragmented suing a graded collision energy profile.

QToF mass spectrometry:

Ionisation mode:	Positive
Capillary	0.5 or 1.5 kV
Sample cone voltage:	40 V
Source temperature:	120 °C
Desolvation temperature:	500 °C
MS/MS Collision Energies:	20, 30 & 40 V

UHPLC gradient elution:

Mobile phase component A:	Water plus 0.1% formic acid
Mobile phase component B:	Acetonitrile plus 0.1% formic acid
Flow rate:	0.5 mL/min
Gradient program:	Initial: 95% A/5% B<br>0.5 min: 95% A/5% B<br>4 min: 60% A/40% B<br>6 min: 5% A/95% B<br>Re-equilibration time: 1 min
Injection volume:	5 µL
Column:	Waters BEH C_{18}, 50 x 2.1 mm 1.7 µm particle size.
Column temperature:	40 °C
UV detection:	Spectra from 200 to 400 nm collected.

For each timepoint of the experiment, peak areas of the parent and any identified metabolites were determined using both the diode array signal and the extracted ion mass spectrometer signal for the identified mass. This was plotted against time to show how the metabolism profile changed during the course of the experiment.

Determination of bactericidal efficacy of compounds *in vitro*:

M. tuberculosis H37Rv was grown in Middlebrook 7H9 medium supplemented with 0.1 mM propionate/0.2% D-glucose/0.2% glycerol/0.5% BSA fraction V/0.05% Tween 80 to an OD 650 $_{nm}$ of 0.4. A single cell suspension was generated by filtration through a 5 µm filter and diluted to 3.4×10^6 CFU/mL in 24-well tissue culture plates (0.5 mL/well) containing DMSO or drug. Individual 24-well plates were set up for every time point. Each condition was tested in triplicate for each time point. The bacterial cell number at the start of drug treatment was enumerated by plating of appropriate cell dilutions from the DMSO control on 7H11/OADC plates. The 24-well plates were incubated at 37 ˚C at 100rpm in ziplock bags. At days 3 and 7 after initiation of drug treatment, appropriate dilutions of cells were made in Middlebrook 7H9 medium supplemented with 0.1 mM propionate/0.2% D-glucose/0.2% glycerol/0.5% BSA fraction V/0.05% Tween 80 and plated on 7H11/OADC plates. Each of the triplicate repeats were plated in duplicate for each dilution. Colonies were enumerated after 3 weeks of incubation at 37 ˚C.

Determination of intramacrophage efficacy of compounds:

M. tuberculosis H37Rv was grown in Middlebrook 7H9 medium supplemented with 0.1 mM propionate/ 0.2% D-glucose/ 0.2% glycerol/ 0.5% BSA fraction V/ 0.05% Tween 80 to an $OD650_{nm}$ of 0.4. Cells were harvested an resuspended in J774 growth medium consisting of DMEM with 4 mM glutamine/4.5 g/L glucose 0.5 mM sodium pyruvate/15 mM HEPES/10% fetal bovine serum. The cell suspension was filtered through a 5 µm filter and diluted to 3×10^5 CFU/mL in this medium. J774A.1

macrophages, plated at $\times 10^4$ cell/well in 24-well tissue culture plates (1 mL/well in J774 growth medium)(Nunclon) were infected with 0.1 mL of the diluted *M. tuberculosis* suspension overnight. Cell monolayers were washed 3 times in pre-warmed DMEM, three wells were used for bacterial burden enumeration as outlined below and to the remaining wells, 1 mL J774 growth medium containing DMSO (0.4%) or drugs at their final desired concentration added. Each condition was performed in triplicate for each time point. Medium was with fresh medium (with appropriate DMSO or drugs) every three days. On days 3 and 7 after initiation of drug treatment, the growth medium was removed and replaced with 1 mL 7H9/Glu/BSA/Tx containing 0.1% SDS. Cell homogenates were diluted in 7H9/Glu/BSA/Tx and appropriate dilutions plated in duplicate on 7H11/OADC plates.

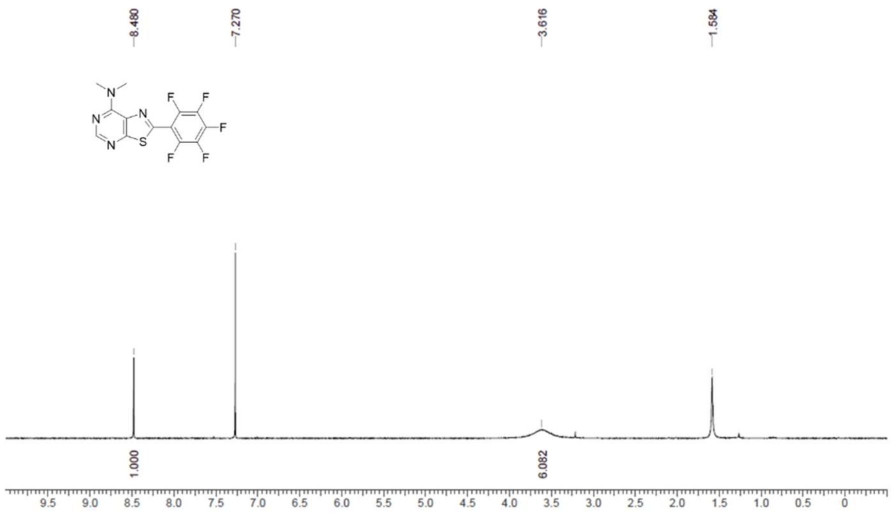

Supplementary Spectra 1: ^{1}H NMR spectra of compound **1** (rt, in $CDCl_3$)

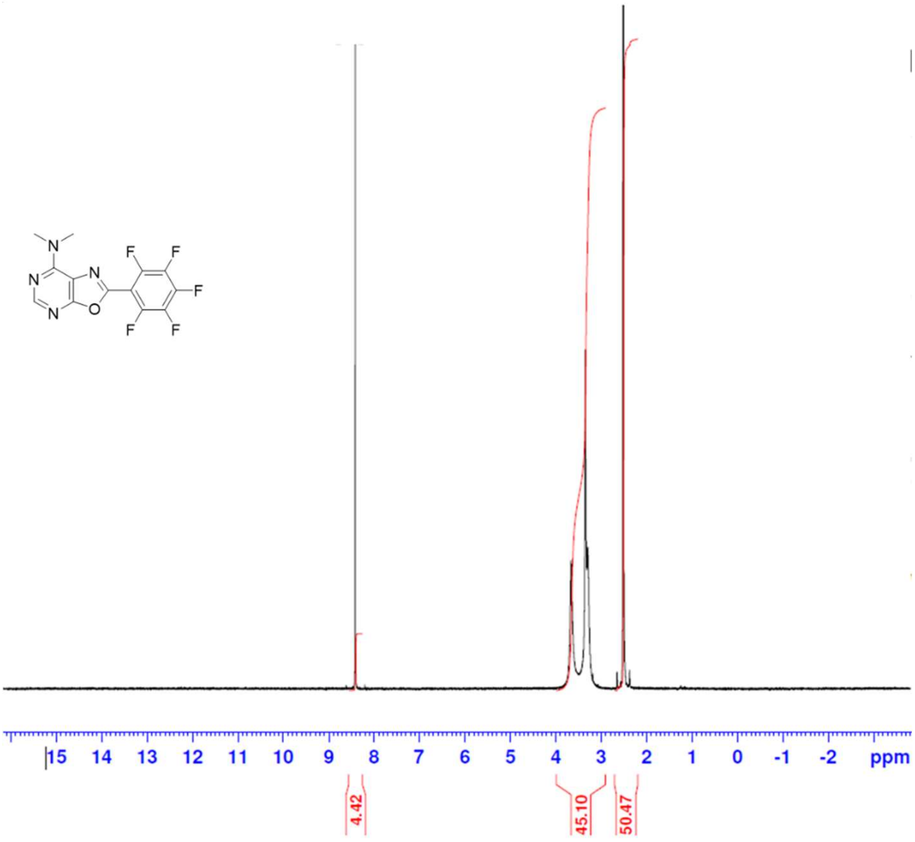

Supplementary Spectra 2: ^{1}H NMR spectra of compound **6** (rt, in DMSO-d_6)

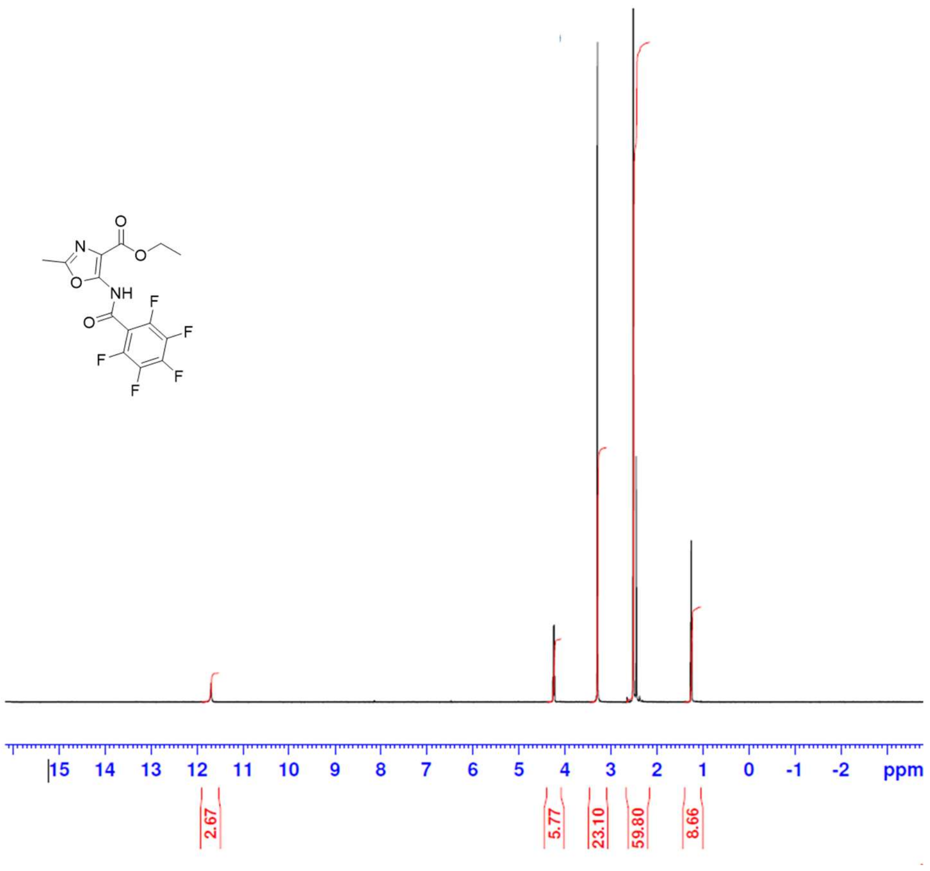

Supplementary Spectra 3: ^{1}H NMR spectra of compound **24** (rt, in DMSO-d_6)

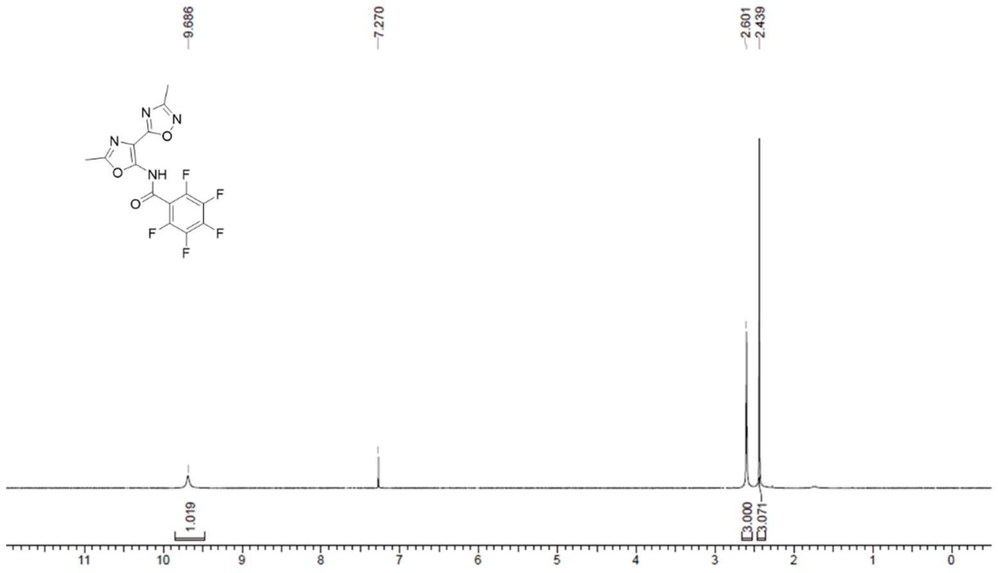

Supplementary Spectra 4: ^{1}H NMR spectra of compound **43** (rt, in DMSO-d_6)

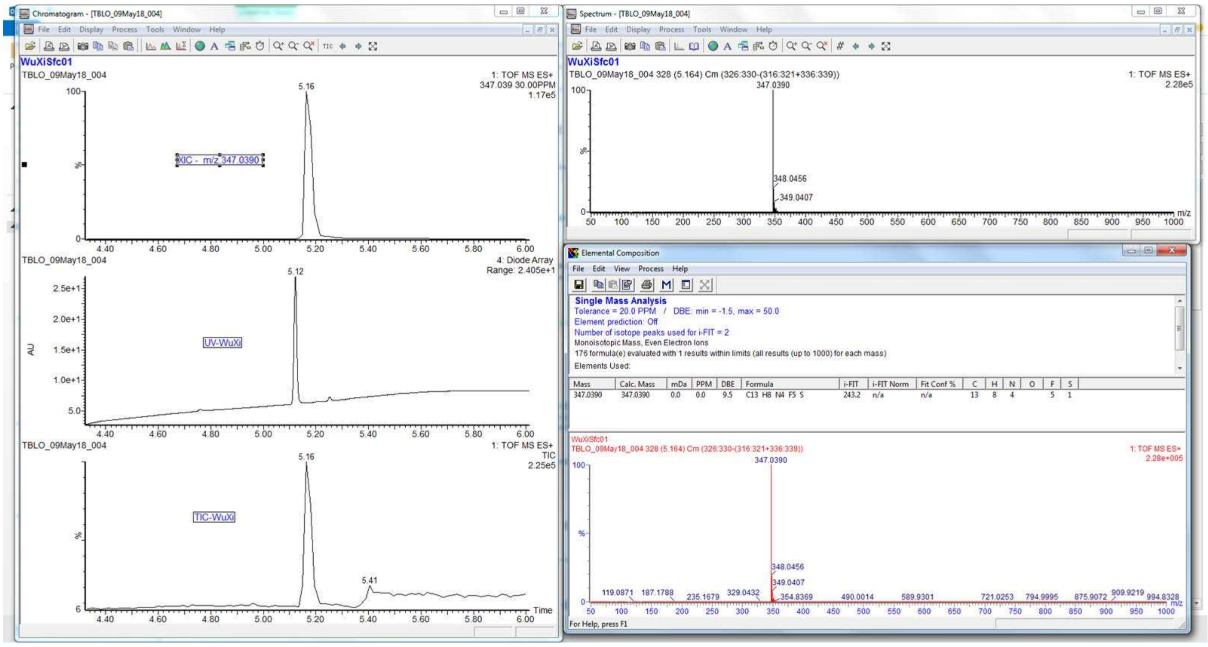

Supplementary Spectra 5: HRMS of compound **1**

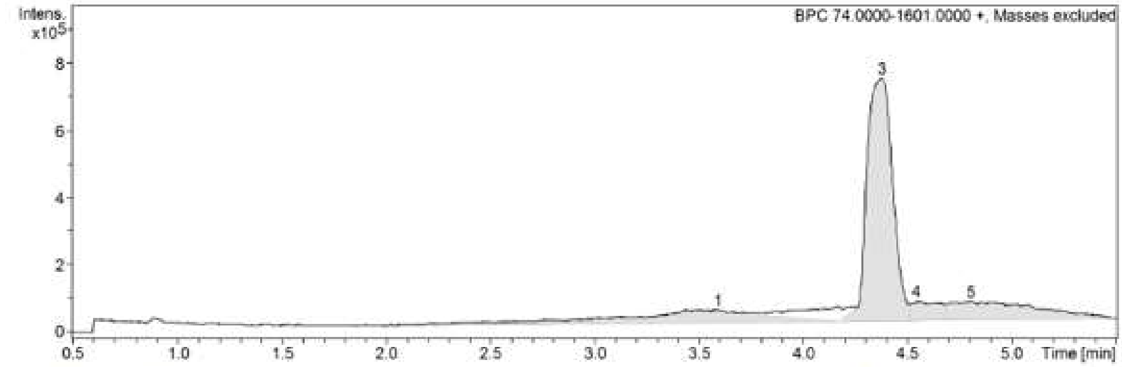

Sample Description

Method 1-microtof-2 Identify Compounds LCMS Pos 5-95_8131.m

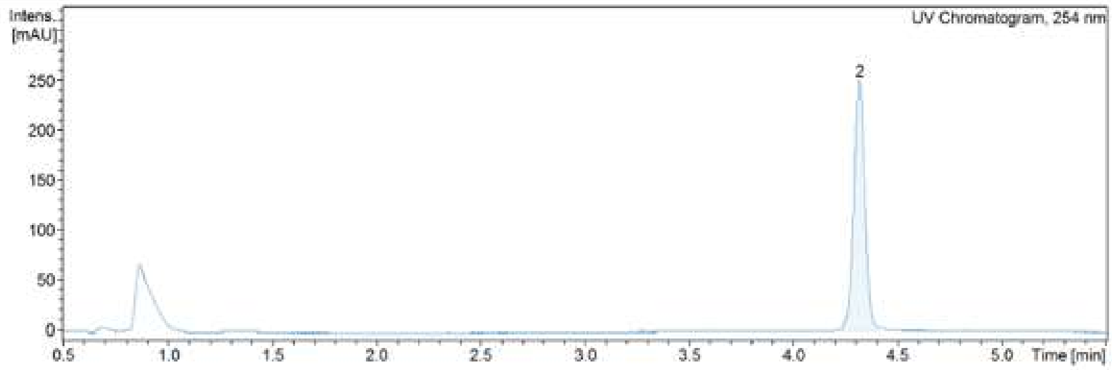

#	RT [min]	Area Frac. %	Chromatogram
1	3.6	18.98	BPC 74.0000-1601.0000 +, Masses excluded
2	4.3	100.00	UV Chromatogram, 254 nm
3	4.4	59.94	BPC 74.0000-1601.0000 +, Masses excluded
4	4.5	5.09	BPC 74.0000-1601.0000 +, Masses excluded
5	4.8	15.99	BPC 74.0000-1601.0000 +, Masses excluded

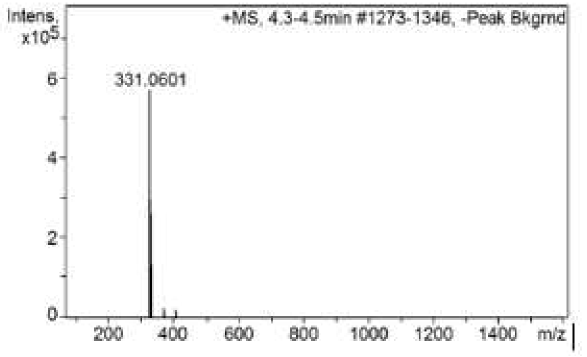

Supplementary Spectra 6: HRMS of compound **6**

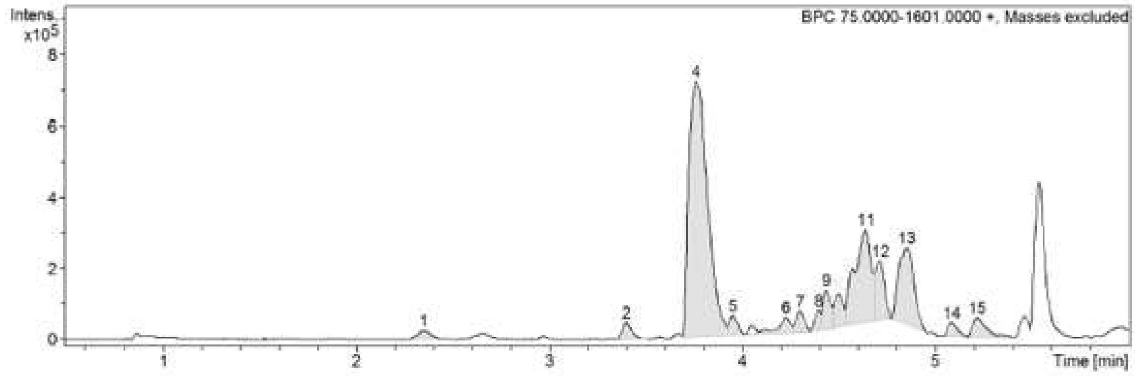

Sample Description

Method 1-microtof-2 Identify Compounds LCMS Pos 5-95_8131.m

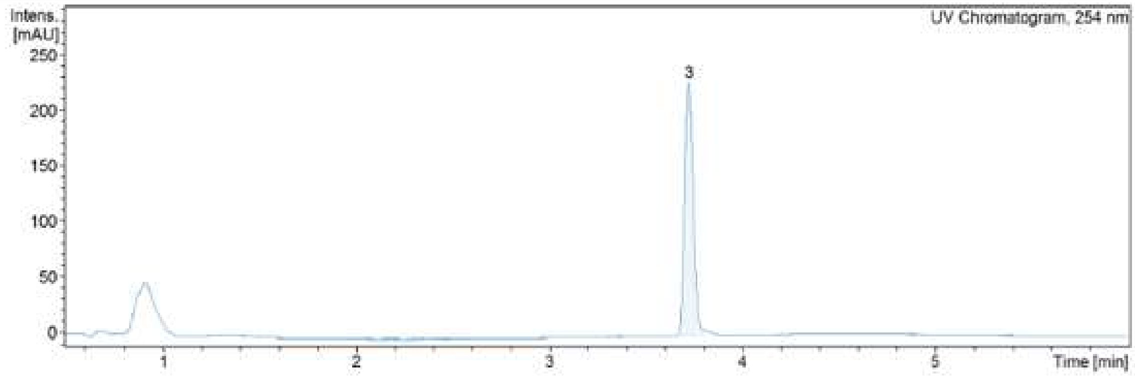

#	RT [min]	Area Frac. %	Chromatogram
1	2.4	1.20	BPC 75.0000-1601.0000 +, Masses excluded
2	3.4	1.56	BPC 75.0000-1601.0000 +, Masses excluded
3	3.7	100.00	UV Chromatogram, 254 nm
4	3.8	48.13	BPC 75.0000-1601.0000 +, Masses excluded
5	4.0	1.63	BPC 75.0000-1601.0000 +, Masses excluded
6	4.2	1.87	BPC 75.0000-1601.0000 +, Masses excluded
7	4.3	1.77	BPC 75.0000-1601.0000 +, Masses excluded
8	4.4	1.22	BPC 75.0000-1601.0000 +, Masses excluded

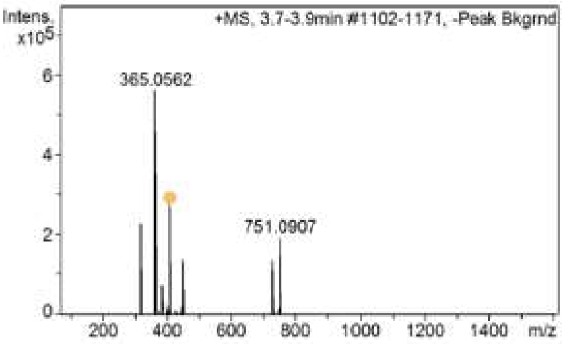

Supplementary Spectra 7: HRMS of compound **24**

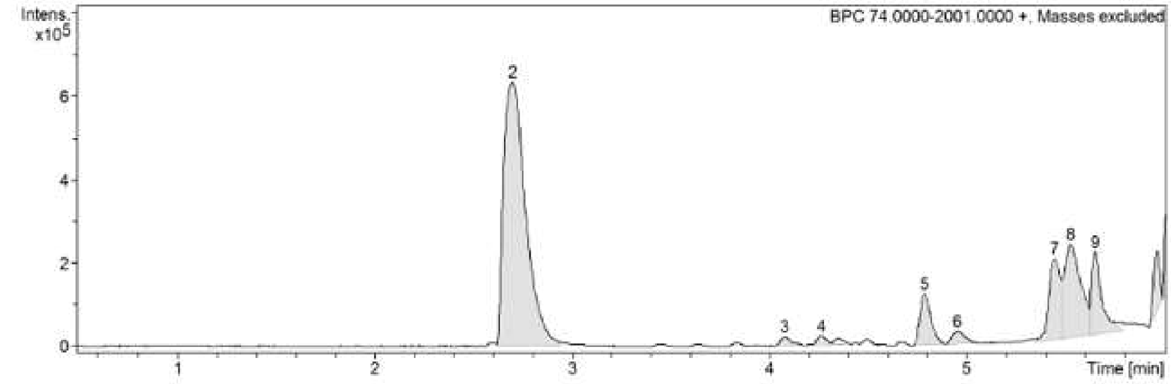

Sample Description

Method 3-microtof-2 Identify Compounds BASIC LCMS Pos 5-95_8132.m

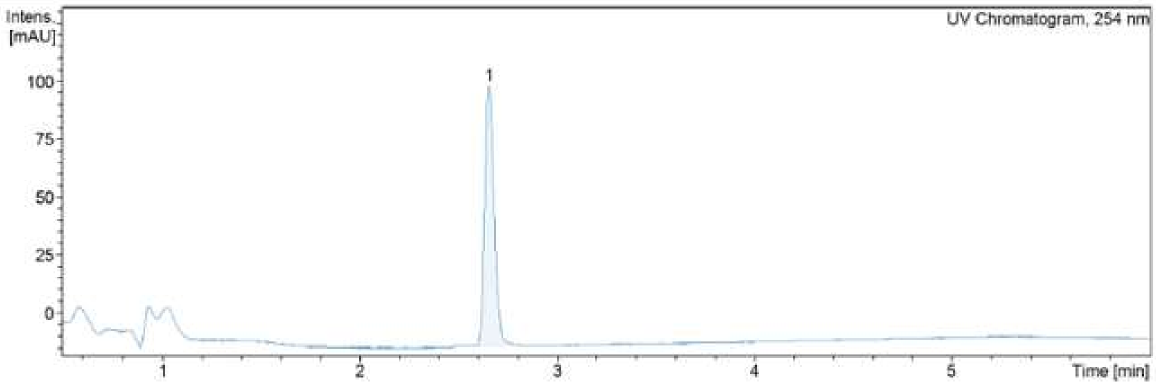

#	RT [min]	Area Frac. %	Chromatogram
1	2.7	100.000	UV Chromatogram, 254 nm
2	2.7	55.657	BPC 74.0000-2001.0000 +, Masses excluded
3	4.1	0.924	BPC 74.0000-2001.0000 +, Masses excluded
4	4.3	0.922	BPC 74.0000-2001.0000 +, Masses excluded
5	4.8	5.353	BPC 74.0000-2001.0000 +, Masses excluded
6	5.0	1.619	BPC 74.0000-2001.0000 +, Masses excluded
7	5.4	8.915	BPC 74.0000-2001.0000 +, Masses excluded
8	5.5	14.619	BPC 74.0000-2001.0000 +, Masses excluded

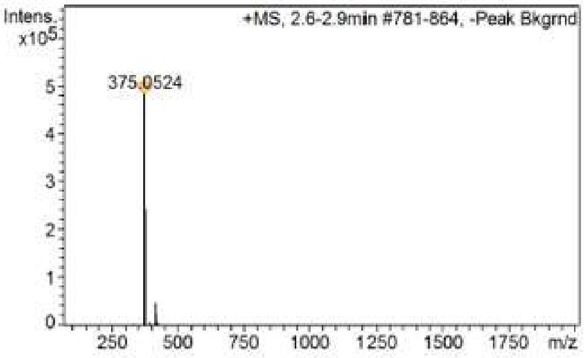

Supplementary Spectra 8: HRMS of compound **43**
